# dbCNS: A New Database for Conserved Noncoding Sequences

**DOI:** 10.1093/molbev/msaa296

**Published:** 2020-11-16

**Authors:** Jun Inoue, Naruya Saitou

**Affiliations:** 1 Population Genetics Laboratory, Department of Genomics and Evolutionary Biology, National Institute of Genetics, Mishima, Japan; 2 Center for Earth Surface System Dynamics, Atmosphere and Ocean Research Institute, University of Tokyo, Kashiwa, Japan; 3 Department of Okinawa Bioinformation Bank, Faculty of Medicine, University of the Ryukyus, Okinawa, Japan

**Keywords:** dbCNS, conserved noncoding sequences, vertebrates, single-nucleotide polymorphisms, *cis*-regulatory elements

## Abstract

We developed dbCNS (http://yamasati.nig.ac.jp/dbcns), a new database for conserved noncoding sequences (CNSs). CNSs exist in many eukaryotes and are assumed to be involved in protein expression control. Version 1 of dbCNS, introduced here, includes a powerful and precise CNS identification pipeline for multiple vertebrate genomes. Mutations in CNSs may induce morphological changes and cause genetic diseases. For this reason, many vertebrate CNSs have been identified, with special reference to primate genomes. We integrated ∼6.9 million CNSs from many vertebrate genomes into dbCNS, which allows users to extract CNSs near genes of interest using keyword searches. In addition to CNSs, dbCNS contains published genome sequences of 161 species. With purposeful taxonomic sampling of genomes, users can employ CNSs as queries to reconstruct CNS alignments and phylogenetic trees, to evaluate CNS modifications, acquisitions, and losses, and to roughly identify species with CNSs having accelerated substitution rates. dbCNS also produces links to dbSNP for searching pathogenic single-nucleotide polymorphisms in human CNSs. Thus, dbCNS connects morphological changes with genetic diseases. A test analysis using 38 gnathostome genomes was accomplished within 30 s. dbCNS results can evaluate CNSs identified by other stand-alone programs using genome-scale data.

## Introduction

It has long been speculated that protein noncoding regions are involved in protein expression control (King and Wilson 1975). Genomic sequence comparisons between humans and fugu (pufferfish) revealed that a class of noncoding genomic sequences displays an extra degree of conservation among vertebrate genomes (Aparicio et al. 1995). Although conserved sequences of noncoding regions are identified in the literature with different names, such as CNEs (conserved noncoding elements: Woolfe et al. 2005) or UCEs (ultraconserved elements: Bejerano et al. 2004), the prevailing view is that these sets of sequences are largely overlapping in their genesis and functions and that their evolutionary dynamics are largely unknown (Polychronopoulos et al. 2017; Saitou 2018). In this article, we call all such sequences “conserved noncoding sequences” or CNSs. CNSs tend to cluster in the vicinity of genes with regulatory roles in multicellular development and differentiation (Sumiyama and Saitou 2011). In fact, CNS mutations may result in vertebrate morphological changes or may cause human genetic diseases (Polychronopoulos et al. 2017).

The recent rapid growth of genome data has made it possible to identify CNSs particularly among vertebrates. For the last 10 years, we have been studying CNSs among various taxonomic groups, such as plants (Hettiarachchi et al. 2014), vertebrates (Matsunami et al. 2010; Matsunami and Saitou 2013; Hettiarachchi and Saitou 2016), mammals (Babarinde and Saitou 2013), rodents (Takahashi and Saitou 2012), and primates (Takahashi and Saitou 2012; Babarinde and Saitou 2016; Saber et al. 2016; Saber and Saitou 2017). Some of them examined the contribution of putative regulatory CNSs in defining clade-specific phenotypes (Babarinde and Saitou 2013; Matsunami and Saitou 2013; Saber and Saitou 2017). Recently, CNSs have been identified as evolutionarily conserved elements, based on genome alignments using tools such as PhastCons (Siepel et al. 2005) and GERP (Davydov et al. 2010). However, preparation of genome alignments and analyses using such tools are computationally intensive.

As far as we know, there are only four CNS-related databases (last accessed November 30, 2020). The VISTA Browser (https://enhancer.lbl.gov) distributes CNSs identified in humans and mice that have been tested in vivo for enhancer activity (Visel et al. 2007), and VISTA’s web tools (http://genome.lbl.gov/vista/index.shtml) allow inspection and comparison of sequence conservation profiles across specified genomic regions in a user-customizable manner (Brudno et al. 2007). ANCORA (http://ancora.genereg.net), developed by Engstrom et al. (2008), distributes metazoan CNSs identified by scanning pairwise genome alignments (e.g., humans vs. chickens). This web resource can be used to discover developmental regulatory genes and to distinguish their chromosomal regulatory domains by viewing CNS locations and densities in the UCSC Genome Browser (Kuhn et al. 2007). Persampieri et al. (2008) developed cneViewer (http://bioinformatics.bc.edu/chuanglab/cneViewer) for noncoding DNA elements in zebrafish. Its key feature is the ability to search for CNSs that may be relevant to tissue-specific gene regulation, based on known developmental expression patterns of nearby genes. Dimitrieva and Bucher (2013) developed UCNEbase (https://ccg.epfl.ch/UCNEbase) that identifies 4,351 CNSs shared among 18 vertebrates. UCNEbase features a consistent naming scheme to identify elements across genomes, along with descriptive statistics of element distributions and synteny maps. These databases, however, are not frequently updated and do not accommodate demands to identify CNSs using user-provided sequences as queries in specific taxonomic sampling. Moreover, no database exists to link causal single-nucleotide polymorphisms (SNPs) to morphological changes and/or genetic diseases.

## New Approaches

By integrating CNSs among vertebrates scattered among databases and journal articles, we created a new database called dbCNS (http://yamasati.nig.ac.jp/dbcns; last accessed November 30, 2020). dbCNS allows users not only to extract published CNSs as regulatory candidates of interest but also to search for CNSs in user-selected genomes. For this purpose, dbCNS also contains some invertebrate genomes. dbCNS automatically produces coordinates, multiple alignments, and phylogenetic trees. Using these outputs, users can evaluate extracted sequences as CNSs within areas of interest and can detect potential CNSs with accelerated substitution rates. Users can also count identical CNSs in a genome in dbCNS, something no other database has been able to do, because of their reliance on genome alignments to identify CNSs.

## Results and Discussion

### Interface and Two Query Search Modes

**Fig. 1.**
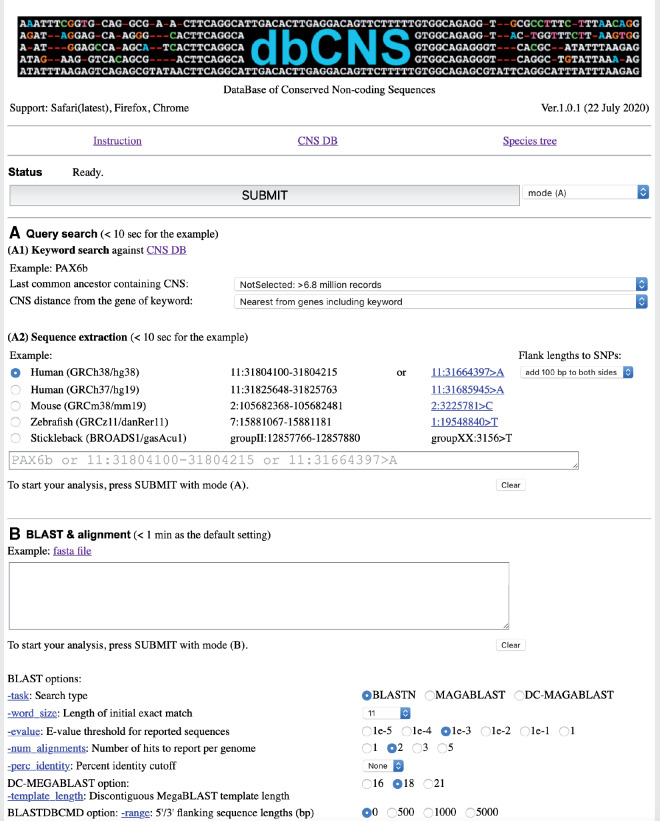
The front page of dbCNS.

**Fig. 2.**
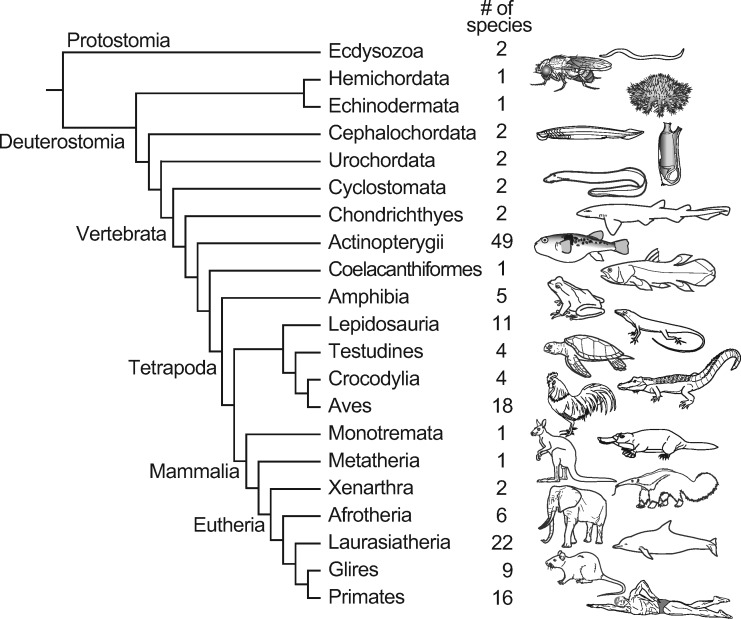
Phylogenetic relationships of 180 genomes for which sequence data are included in dbCNS.

Figure 1 shows the upper part of the top page of dbCNS version 1. dbCNS contains ∼6.9 million CNSs published in journals and in databases (see table 1), and it also contains sequences of 162 vertebrate and nine invertebrate genomes downloaded from Ensembl (http://www.ensembl.org) and NCBI (https://www.ncbi.nlm.nih.gov). Phylogenetic relationships of the genomic sequence data sets in dbCNS are shown in figure 2. dbCNS holds a list of gene coordinates for each species to identify the nearest genes (upstream and downstream) of BLAST hits. Two main functions are available in dbCNS: (A) Query search and (B) BLAST and alignment. Flowcharts are shown in supplementary figure S1*A*, Supplementary Material online. The web design of dbCNS follows that of ORTHOSCOPE, developed by Inoue and Satoh (2019) (https://www.orthoscope.jp).

**Table 1.** CNSs Stored in dbCNS.

Clade	Comparison	Sequence	No. of CNSs	Data Source
Vertebrata	18 vertebrates	Human (hg19)	4,351	https://ccg.epfl.ch//UCNEbase
Gnathostomata	19 gnathostomes	Human (hg19)	208	Matsunami et al (2010) ^a^
Bony vertebrates	Human, Zebrafish	Human (hg38)	18,852	ANCORA^b^ (70% identity over 50 columns)
Actinopterygii				
Clupeocephala	Zebrafish,	Zebrafish	200,099	ANCORA^c^
	Stickleback	(danRer10)		(70% identity over 30 columns)
	Zebrafish, Stickleback	Stickleback (BROADS1)	175,168	ANCORA^c^ (70% identity over 30 columns)
Sarcopterygii	8 tetrapods	Human ortho (GRCh37)	7,650	Matsunami and Saitou (2013)
	8 tetrapods	Human para (GRCh37)	309	Matsunami and Saitou (2013)
Amniota	Human, Chicken	Human (hg38)	12,041	ANCORA^d^ (100% identity over 50 columns)
Mammalia	20 mammals	Human (hg38)	2,752	UCSC Genome Browser^e^ (phastCons100way, <1,000 bp)
Boreoeutheria	Human, Dog	Human (hg38)	95,462	ANCORA^f^ (100% identity over 50 columns)
Laurasiatheria	Dog, Horse	Dog	5,284,452	ANCORA^g^ (80% identity over 50 columns)
	Dog, Horse	Dog	126,218	ANCORA^h^
		(canFam3)		(100% identity over 50 columns)
Euarchontoglires	Human, Mouse	Human (hg38)	946,151	ANCORA^i^ (80% identity over 50 columns)
	Human, Rat, Mouse	Human (hg19)	481	Bejerano et al (2004)
	Rodentia Mouse,	Rat Mouse (mm10)	21,128	Takahashi and Saitou (2012)
Primates				
Simiiformes	Human, Marmoset	Human (hg38)	8,198	Takahashi and Saitou (2012)
Hominoidea	5 hominoids	Human (GRCh37)	679	Saber and Saitou (2017)
Hominidae	4 hominids	Human (GRCh37)	1,658	Saber et al. (2016)

a Hox clusters only.

b http://ancora.genereg.net/downloads/hg38/vs_zebrafish/HCNE_hg38_danRer7_70pc_50col.bed.gz.

c http://ancora.genereg.net/downloads/danRer10/vs_stickleback/HCNE_danRer10_gasAcu1_70pc_30col.bed.gz.

d http://ancora.genereg.net/downloads/hg38/vs_chicken/HCNE_hg38_galGal4_100pc_50col.bed.

e https://genome.ucsc.edu/cgi-bin/hgTables.

f http://ancora.genereg.net/downloads/hg38/vs_dog/HCNE_hg38_canFam3_100pc_50col.bed.gz.

g http://ancora.genereg.net/downloads/canFam3/vs_horse/HCNE_canFam3_equCab2_80pc_50col.bed.gz.

h http://ancora.genereg.net/downloads/canFam3/vs_horse/HCNE_canFam3_equCab2_100pc_50col.bed.gz.

i http://ancora.genereg.net/downloads/hg38/vs_mouse/HCNE_hg38_mm10_80pc_50col.bed.gz.

There are two query search modes (A1 and A2) in dbCNS. When a keyword is provided by the user, dbCNS collects CNSs near the gene of interest in “Keyword search” mode. For this purpose, each record of the CNS database has a name line, including the name of the nearest gene locus (see example in supplementary fig. S1*B*, Supplementary Material online). By finding the keyword in name lines, dbCNS lists search results as output. An example output of 195 hits for the keyword “HoxA1” is shown in supplementary figure S2, Supplementary Material online. One can download a tab-separated file from the link shown after “Download tab-separated file” located at the top of this output. dbCNS also allows users to link the potential target gene and CNSs with a user-specified distance with the option “CNS distance from the gene of keyword.” When a coordinate is provided by the user in “Sequence extraction” mode, dbCNS extracts the corresponding sequence from the genome data of a selected model organism with BLASTDBCMD (Altschul et al. 1990). An example of output for “7:27097212-27097599” as the coordinates of a 388-bp sequence at chromosome 7 for the HoxA1-related CNS (Matsunami et al. 2010) from the human genome, build GRCh38/hg38, is shown in supplementary figure S3*A*, Supplementary Material online. Alternatively, when an SNP is provided with its coordinates, dbCNS generates a sequence consisting of the SNP with 100-bp fragments both 5′ upstream and 3′ downstream. Fragment lengths can be selected with the “Flank lengths to SNPs” option. Example output for “11:31664397>A” as the coordinate at chromosome 11 for the human genome, build GRCh38/hg38, is shown in supplementary figure S3*B*, Supplementary Material online. This SNP C>A at rs606231388 in dbSNP (http://www.ncbi.nlm.nih.gov/SNP) causes the human ocular disease, aniridia (Bhatia et al. 2013; see “Case Study 1” below).

### BLAST and Multiple Alignment

In the “BLAST & alignment” mode of dbCNS, a CNS should be provided in FASTA format. An example CNS (a 201-bp sequence in the human Simo enhancer region: GRCh38_11-31664297-31664497) is shown in http://yamasati.nig.ac.jp/dbcns/examples/exampleQuerySeq.html. A BLAST search (Altschul et al. 1990) is first conducted using that query sequence in dbCNS. BLAST hits are then multiply aligned using MAFFT (Katoh and Standley 2013) and TRIMAL (Capella-Gutierrez et al. 2009), and the corresponding neighbor-joining tree (Saitou and Nei 1987) for these multiply aligned sequences is generated using APE 3.0 (Popescu et al. 2012) automatically. The most parameter-rich model in the program, the TN 93 model (Tamura and Nei 1993), is applied with a gamma distributed rate for site heterogeneity (Yang 1994).

Before starting an analysis, the user needs to set parameters in “BLAST options” for the similarity search: “-tasks” sets parameters to typical values for a specific type of search. “BLASTN” finds regions of local similarity between nucleotide sequences. For much longer DNA sequences, “MEGABLAST” can be selected for intraspecific comparisons with large “word-size” (see below) and “DC-MEGABLAST” to find more distant (interspecific) sequences. “-word_size” determines the length of an initial exact match. “-evalue” is a threshold expect value for saving hits, and “-num_alignments” determines the number of BLAST hits report per genome. “perc_identity” discards alignments that do not meet a minimum % identity. In “DC-MEGABLAST option” using DC-MEGABLAST, “template_length” determines lengths of templates. In “BLASTDBCMD option,” using BLASTDBCMD, “-range” provides lengths of 5′ upstream and 3′ downstream sequences for extracting flanking sequences of BLAST hits. Taxonomic sampling is determined by selecting species in “Genome taxon sampling” or uploading a batch file (see Appendix for details of batch file description).

**Fig. 3.**
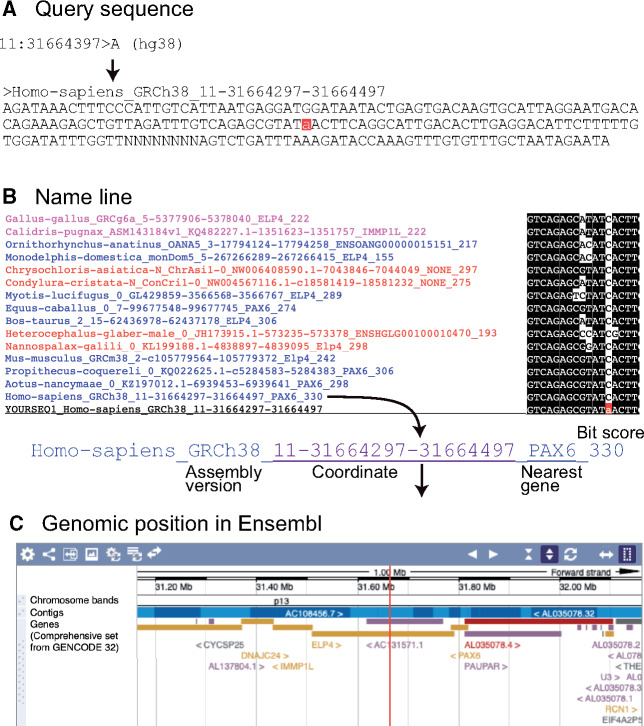
Results of dbCNS analyses for an SNP that causes aniridia. (*A*) Query sequence. The letter with a red background indicates the SNP site. (*B*) Name line of alignment. The name line includes the nearest gene of the BLAST hit identified by the transcription start site (TSS). Links of coordinates and nearest genes lead to the Ensemble genome browser. (*C*) Genomic position in Ensembl.

If we submit example file to dbCNS, the result file is created after ∼33 s of computation. Figure 3 shows the flow of information in this example. The summary output (supplementary fig. S4*A*, Supplementary Material online) can be seen by clicking the link after “Status Finished,” just above the “SUBMIT” button. This summary output shows the query sequence, numbers of BLAST hits for each selected genome sequence, multiple alignment of BLAST hits, a phylogenetic tree, and setting details. In addition to numbers of BLAST hits for each species, dbCNS provides coordinates and nearest genes in name lines. These are linked to the Ensembl genome browser to show their genomic positions. In the resultant alignment, poorly aligned sites are identified using TRIMAL with the option “-gappyout.” Such sites are marked with “0,” whereas unambiguously aligned sites are identified with “1.” One can download the output (in zip format) from the link shown after “Download,” located at the top of this summary output. This detailed output folder contains files, including an analytical summary, a multiple alignment, and a phylogenetic tree.

### Three Case Studies Related to PAX6

We demonstrate the utility of dbCNS using three case studies related to the *PAX6* gene, with taxonomic sampling relative to gnathostomes and teleosts. The multifunctional developmental regulator, PAX6, is essential to development and maintenance of the central nervous system (Osumi et al. 2008), the olfactory system (Nomura et al. 2007), and the pancreas (Hart et al. 2013). This gene is best known for its critical role in eye development (Gehring and Ikeo 1999; Cvekl and Callaerts 2017). In nearly every species that uses vision, development of the eyes is critically dependent on the presence and dosage of PAX6 (Gehring 2005). Extensive effort has gone into characterizing spatiotemporal regulation of *PAX6* expression (Kleinjan et al. 2006). A genomic regulatory block has been identified by finding long syntenic arrays of CNSs clustered around this block (Kikuta et al. 2007).

#### Case Study 1: Construction of CNS Alignment, Including an SNP That Causes Human Disease

Based on coordinates of human SNP sites, dbCNS can construct multiple sequence alignments to evaluate evolutionary conservation of genomic regions, including specified sites. Aniridia (OMIM ID 106210) is a panocular disease characterized by a variable degree of iris/foveal hypoplasia, nystagmus, and ciliary body abnormalities. In a patient with aniridia and no exonic mutations or chromosomal abnormalities, direct sequencing of *cis*-regulatory elements active in various eye tissues revealed a single-nucleotide change in a conserved ocular enhancer, SIMO, located 150 kb downstream from *PAX6* (Bhatia et al. 2013). The SNP that causes aniridia (Bhatia et al. 2013) is C>A at rs606231388 in dbSNP.

As we already showed in an example of “sequence extraction mode,” dbCNS extracted a 201-bp sequence, including this SNP site, from the reference human genome sequence (hg38) using “11:31664397>A” as a keyword (supplementary fig. S3*B*, Supplementary Material online). Using this sequence, output of the BLAST & alignment mode was generated with 38 gnathostome genomes (supplementary fig. S4*A*, Supplementary Material online). In this analysis, the “-num_alignments” option was set at two in order to count identified CNSs in each species. As a result, dbCNS identified at most one BLAST hit for each species (shown in [# of blast hits]) and automatically aligned them. The alignment showed that all BLAST hits of gnathostomes contain the PAX6 binding site and belong to the SIMO region (Bhatia et al. 2013), except for the partial sequence of *Erpetoichthys calabaricus* (reedfish). Then, we confirmed that these BLAST hits are identical to the human query sequence and form a CNS as a highly conserved part of the SIMO enhancer (Antosova et al. 2016).

**Fig. 4.**
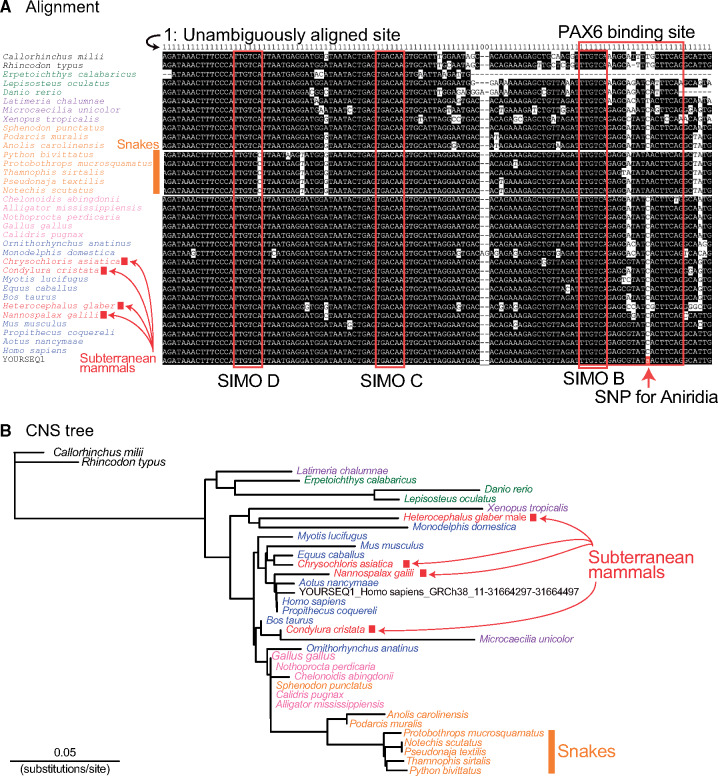
(*A*) Alignment of the main part of the SIMO region (supplementary fig. S4, Supplementary Material online). In the query sequence, YOURSEQ1, the SNP site is highlighted with a red background. (*B*) Phylogenetic tree based on sequences of the SIMO region (121 sites).

The alignment (fig. 4*A*) confirmed that in this aniridia-related site, most tetrapods share the same nucleotide C and the mutation changed the human nucleotide from C>A. In addition, the alignment showed that all five snakes share A at this site. In this case, dbCNS can be used to detect CNS candidates with accelerated substitution rates. The estimated CNS tree (fig. 4*B*) suggested that in the snake lineage, branches leading to the common ancestor of the five snakes possessed an increased number of substitutions compared with peripheral branches. These findings imply that characteristics of the snake SIMO region were fixed before divergence of the major snake lineages. This CNS diversification in snake ancestors is consistent with their possible subterranean lifestyle (Da Silva et al. 2018) and the loss of opsins in the early stage of snake evolution (Simoes et al. 2015). In contrast, four subterranean mammals (species names are shown in red) showing convergent eye degeneration shared the nucleotide C with most other tetrapods (fig. 4*A*). In subterranean mammals, several CNSs near *PAX6* loci and other transcription factors important for eye development exhibit accelerated substitution rates (Partha et al. 2017). In this analysis of the SIMO region, an accelerated substitution rate was suggested for the lineage leading to the subterranean mammal, *Heterocephalus glaber* (naked mole rat), compared with other eutherians (fig. 4*B*). The naked mole rat sequence is not placed next to related species probably due to its high sequence divergence. For more sophisticated analyses of accelerated substitution rates with user-defined tree topologies, users can employ state-of-the-art methods, such as RERconverge (Kowalczyk et al. 2019), using dbCNS outputs.

dbCNS produces a link to dbSNP (build 153) using BLAST-hit coordinates derived from the human genome (hg38). By clicking the link “11:31664297-31664497” located below “Human SNP in dbSNP:” in the output html file (supplementary fig. S4*A*, Supplementary Material online), it was confirmed that the aniridia-causing SNP site (rs606231388) is located in this human BLAST hit. Moreover, dbCNS can analyze SNPs identified in genome-wide association studies. For example, nasopharyngeal carcinoma-related SNP (Madelaine et al. 2018) can be analyzed using “3:169364845>A” (hg38) as a keyword (supplementary fig. S4*B*, Supplementary Material online).

#### Case Study 2: Detection of CNSs in Gnathostome Genomes

dbCNS can evaluate the existence or number of CNSs in genomes. In addition to the SIMO region, several CNSs were annotated as *cis*-regulatory elements that control expression of *PAX6* in various tissues, including the eye. Bhatia et al. (2014) identified CNSs in the *RCN1*–*PAX6* intergenic region by employing a strategy that analyzes gnathostome sequence conservation and tests identified CNSs of the elephant shark for enhancer activity using a combination of zebrafish and mouse transgenic studies.

**Fig. 5.**
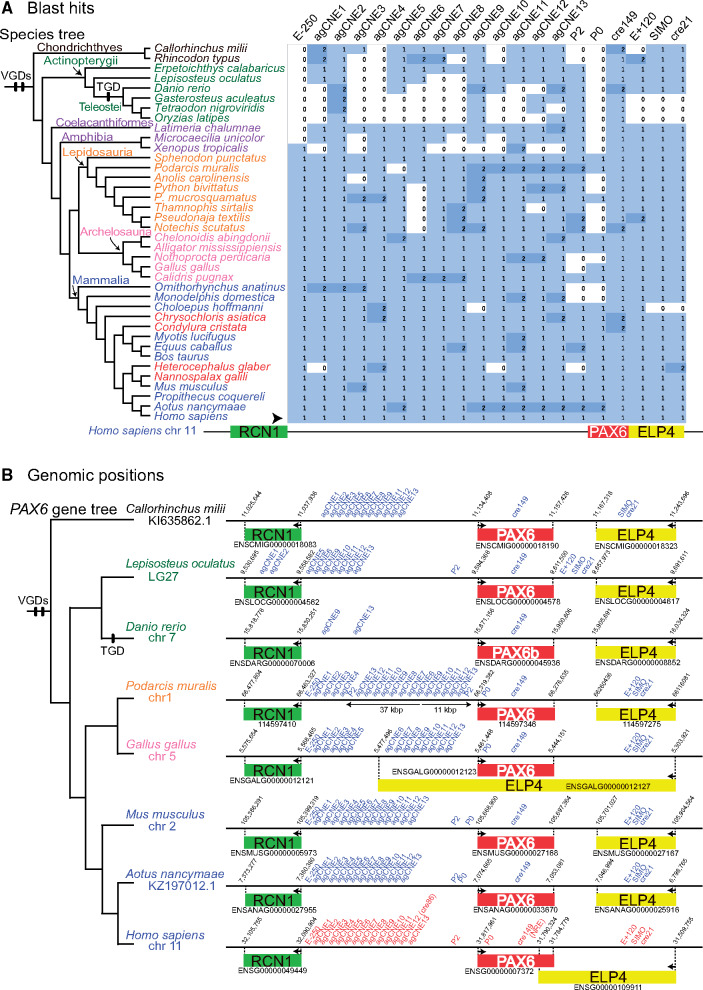
Results of gnathostome analyses. (*A*) BLAST hits for CNS queries around human *PAX6* locus. An arrowhead indicates the row of humans, sequences of which were used as queries. Phylogenetic positions of whole-genome duplications (VGD, vertebrate genome duplication; TGD, teleost genome duplication) follow Braasch and Postlethwait (2012). The heatmap was summarized by using a script available from the dbCNS instruction page. (*B*) Overview of CNS positions around *PAX6* loci. The black line represents DNA. Red letters indicate CNS queries in humans and blue letters indicate CNS BLAST hits in nonhuman gnathostomes. Rectangles indicate the *PAX6* locus (red) and adjacent *RCN1* (green) and *ELP4* loci (yellow). In *P. muralis*, thin horizontal arrows indicate putative duplicated regions. Arrows within gene loci indicate TSS.

Thus, we examined CNSs shared among other gnathostomes. Using 20 published CNS coordinates (supplementary table S1, Supplementary Material online), the dbCNS “Sequence extraction” mode reported CNSs from human genome data (supplementary table S2*A*, Supplementary Material online). The existence of identical CNSs was then evaluated for 38 gnathostome genomes using extracted CNSs as queries in the “BLAST & alignment” mode. The option “-num_alignments” was set at two to detect duplicated CNSs in each genome. A method for conducting multiple analyses and summarizing results is shown on the instruction page. BLAST hits were detected for all species analyzed only for agCNE2 and cre149, whereas all four teleost genomes lacked nine CNSs (fig. 5*A*). Although most BLAST hits were single, two hits were detected for several species, such as *Podarcis muralis* (common wall lizard), *Equus caballus* (horse), and *Aotus nancymaae* (Nancy Ma's night monkey).

These BLAST hits were mapped onto genomic regions of eight gnathostome species to determine the presence of CNSs around the *PAX6* locus (fig. 5*B*). Summary statistics from those 20 analyses were generated by using our customized command-line scripts available from the dbCNS instruction page. In addition to their genomic positions, we identified CNSs by evaluating sequence alignments and bit scores. As a result, a single CNS was identified in this region, although *P. muralis* possessed six duplicated CNSs.

The six duplicated CNSs (agCNS9–13 and P2) of *P. muralis* formed a pair of blocks: an 11-kb region consisting of the six CNSs with the same order as in the human genome and a 37-kb region, including additional three CNSs (agCNS6–8) with reversed order. The arrangement of *P. muralis* CNSs appears to show vestiges of duplication and inversion, though CNSs of other gnathostomes were aligned in the same order as in the human genome. This conserved architecture shared among gnathostomes is probably important for the PAX6 system.

To illustrate the novelty of dbCNS, identified CNSs were compared with those estimated using a pioneering web tool in this field, mVISTA (Frazer et al. 2004; http://genome.lbl.gov/vista/mvista/submit.shtml), with special reference to the vestiges of duplication and inversion in the intergenic *RCN1*–*PAX6* region. As far as we know, except for dbCNS, mVISTA is the only other web tool that can identify CNSs in response to user requests. Although mVISTA has a feature to identify novel CNSs, users must prepare sequences of interest for all species. When the intergenic *RCN1*–*PAX6* region was compared among eight species used in gnathostome analyses (fig. 5*B*), mVISTA identified almost the same CNSs sets (supplementary fig. S6*A*, Supplementary Material online) as those identified by dbCNS (supplementary fig. S6*B*, Supplementary Material online). However, despite weak detection of two CNSs (agCNE7 in *Lepisosteus* and agCNE12 in *Danio*) not detected in dbCNS analyses, mVISTA could not identify 11 CNSs located within the 37-kb block of *Podarcis*. Vestiges of duplication and inversion prevented mVISTA from identifying these duplicated CNSs using multiple sequence alignments.

#### Case Study 3: Detection of Lineage-Specific CNSs from Teleost Genomes

**Fig. 6.**
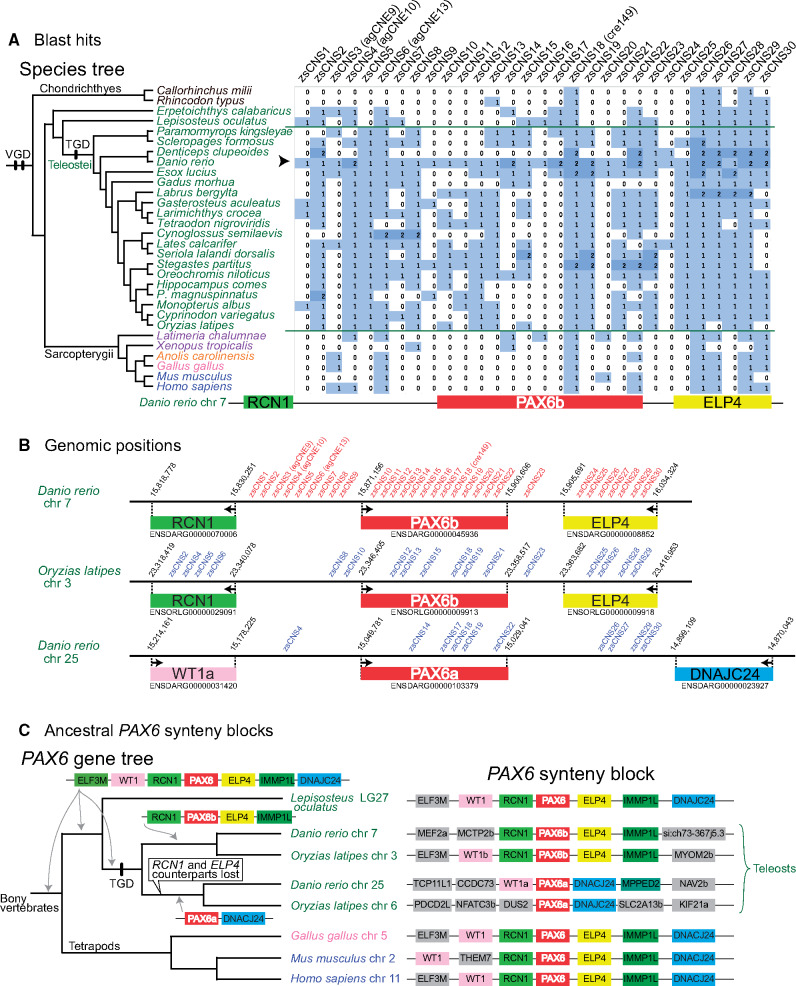
Results of teleost analyses. (*A*) BLAST hits for CNS queries around the zebrafish *PAX6b* locus. All 30 zsCNSs were identified by comparing zebrafish and stickleback genomes in the ANCORA database (Engstrom et al. 2008). An arrowhead indicates the row of *Danio rerio*, sequences of which were used as queries. (*B*) Overview of CNS positions around zebrafish and medaka *PAX6b* and zebrafish *PAX6a* loci. Red letters indicate CNSs queries around zebrafish *PAX6b* loci and blue letters indicate CNS blast hits in other regions. (*C*) Ancestral *PAX6* synteny blocks of teleosts. *PAX6* synteny blocks were compared among bony vertebrate genomes using a conserved synteny browser, Genomicus ver. 98.01 (Muffato et al., 2010). Based on the *PAX6* gene tree (Feiner et al. 2014), hypothetical ancestral states around the *PAX6* locus were reconstructed using parsimony.

dbCNS can detect lineage-specific CNSs. Due to the additional whole genome duplication in the teleost lineage (teleost genome duplication [TGD]) and its consequently increased rate of evolutionary divergence, teleost genomes lack many CNSs identifiable in other vertebrates (Lee et al. 2011). In fact, only three CNSs (agCNE9, agCNE13, and cre149 in fig. 5*B*) were identified around the *PAX6b* locus of the *Danio rerio* (zebrafish) genome in our gnathostome analysis. Using the keyword “PAX6b” for an analysis in the “Keyword search” mode, 164 CNSs conserved between zebrafish (*D. rerio*) and sticklebacks (*Gasterosteus aculeatus*) were listed. Among those, 30 zebrafish sequences (zs1–zs30 in supplementary table S2*B*, Supplementary Material online) had more than four BLAST hits when analyses were conducted in BLAST & alignment mode with our teleost taxon sampling (fig. 6*A*). Results of these 30 analyses are summarized on the right side of figure 6*A*.

Single BLAST hits were detected in many cases (fig. 6*A*). When mapping BLAST hits of *Oryzias latipes* (medaka) chromosome 3 on the region around the *PAX6b* locus, 17 of 30 query CNSs of *D. rerio* had identical CNSs (blue letters in fig. 6*B*). However, two BLAST hits were detected when some of these 30 CNS queries were used, especially for *D. rerio* (fig. 6*A*). When mapping BLAST hits of *D. rerio* around the *PAX6a* locus in chromosome 25, 10 out of 30 query CNSs (blue letters in fig. 6*B*) had TGD-derived counterparts. The teleost *PAX6a* gene is known as the counterpart of the *PAX6b* gene derived from the TGD (Feiner et al. 2014). Given the preservation of the ancestral *PAX6* synteny block around the teleost *PAX6b* locus (fig. 6*C*), and the singleton status of *PAX6b*-adjacent genes, *RCN1* and *ELP4*, in the last common ancestor of teleosts (supplementary fig. S5, Supplementary Material online), counterparts of teleost *RCN1* and *ELP4* genes from TGD are considered lost from the region around the *PAX6a* locus (fig. 6*C*). This disappearance of adjacent genes, *RCN1* and *ELP4* counterparts, supports the hypothesis that in the *D. rerio genome*, these ten CNS counterparts function as regulatory elements of the *PAX6a* gene, as suggested by Kikuta et al. (2007).

## Conclusion

dbCNS (http://yamasati.nig.ac.jp/dbcns), a dynamic web database, enables researchers in gene regulation and human diseases to identify CNSs and their genomic properties. Recently, to identify novel regulatory elements in the whole genome of a single species, high-throughput approaches based on assessing chromatin state (ChIP-seq) and accessibility (e.g., DNaseI-seq, ATAC-seq) have been applied (Martinez-Morales 2016; Roscito et al. 2018). Researchers can examine how such novel elements have changed during evolution of traits and species using dbCNS. In addition, dbCNS can evaluate CNSs identified by other CNS-identification programs using genome-wide data such as PHAST (Hubisz et al. 2011) and CNEr (Tan et al. 2019). Identified CNSs can be used to test their enhancer activity using suitable alternative model systems, such as transgenic reporter zebrafish (Bhatia et al. 2014). Moreover, dbCNS can be used not only to evaluate clade-specific CNSs but also to examine architectures of noncoding sequences. dbCNS currently has several limitations: 1) Analyses are specialized for single-molecule data, not for genome-wide data; 2) users should evaluate alignments, coordinates, and bit scores of BLAST hits to confirm the presence of CNSs in genomic regions of interest; and 3) lengths of query sequences should be <1,000 bp to avoid separation of a target sequence into several BLAST hits. In addition to current vertebrate data, dbCNS will include published CNSs and genome sequences from nonvertebrate metazoans, plants, fungi, and prokaryotes in the near future. Moreover, as our future tasks, the CNS database can be integrated with gene regulatory data from resources such as ENCODE (http://genome.ucsc.edu/ENCODE) and FANTOM (https://fantom.gsc.riken.jp). Use of dbCNS by researchers will facilitate our updates.

## Materials and Methods

The dbCNS server runs on the Linux operating system. An Apache HTTP Server provides web services. Python scripts process all data and requests from users. All these resources have been extensively used and are well supported.

## Supplementary Material

Supplementary data are available at *Molecular Biology and Evolution* online.

## Supplementary Material

msaa296_Supplementary_Data

## Appendix

Batch file example of the taxon sampling list

Callorhinchus-milii_Black

Rhincodon-typus-N_Black

Erpetoichthys-calabaricus_Green

Lepisosteus-oculatus_Green

Danio-rerio_Green

Gasterosteus-aculeatus_Green

Tetraodon-nigroviridis_Green

Oryzias-latipes_Green

Latimeria-chalumnae_Purple

Microcaecilia-unicolor-N_Purple

Xenopus-tropicalis_Purple

Sphenodon-punctatus_Orange

Podarcis-muralis-N_Orange

Anolis-carolinensis_Orange

Python-bivittatus-N_Orange

Protobothrops-mucrosquamatus-N_Orange

Thamnophis-sirtalis-N_Orange

Pseudonaja-textilis-N_Orange

Notechis-scutatus_Orange

Chelonoidis-abingdonii_Magenta

Alligator-mississippiensis-N_Magenta

Nothoprocta-perdicaria_Magenta

Gallus-gallus_Magenta

Calidris-pugnax_Magenta

Ornithorhynchus-anatinus_Blue

Monodelphis-domestica_Blue

Choloepus-hoffmanni_Blue

Chrysochloris-asiatica-N_Red

Condylura-cristata-N_Red

Myotis-lucifugus_Blue

Equus-caballus_Blue

Bos-taurus_Blue

Heterocephalus-glaber-male_Red

Nannospalax-galili_Red

Mus-musculus_Blue

Propithecus-coquereli_Blue

Aotus-nancymaae_Blue

Homo-sapiens_Blue
