# Supplementary material for: dbCNS: A New Database for Conserved Noncoding Sequences
Source: Mol Biol Evol. 2020 Nov 16;38(4):1665–76. doi: 10.1093/molbev/msaa296 (PMC8042745; doi:10.1093/molbev/msaa296)
Supplement: msaa296_Supplementary_Data [file msaa296_supplementary_data.pdf]

Supplementary Materials

Fig. S1

(A) An overview of the dbCNS analysis pipeline.

(B) The name line of each record that includes its coordinate and nearest gene. "Last common ancestor containing CNS" corresponds to the preservation degree of each CNS.

Fig. S2

Output of 195 hits using "HoxA1" as keyword.

Fig. S3

(A) An example output for "7:27097212-27097599"
(B) An example output for "11:31664397>A"

Fig. S4

(A) The output file of the SIMO region analysis.
(B) The output file of an SNP analysis using the nasopharyngeal carcinoma-related SNP.

Fig. S5

(A) *RCN1* gene tree (642 sites).
(B) *ELP4* gene tree (696 sites).
By using ORTHOSCOPE (Inoue and Satoh 2019), neighbor-joining trees were automatically estimated with data sets that comprised nucleotide sequences without 3rd codon positions. Tip names with blue or gray backgrounds indicate query sequences used for ORTHOSCOPE analyses. Numbers beside nodes indicate bootstrap probabilities (> 50%).

Fig. S6

Results of mVISTA analyses for the *RCN1–PAX6* intergenic region of the eight gnathostomes in Fig. 5B.
(A) Visualization of evolutionary sequence conservation by mVISTA. Peaks on the VISTA plot indicate fragments of significant sequence conservation when compared with the *Callorhinchus* sequence.
(B) Identified CNSs in mVISTA (A) and dbCNS (Fig. 5B) analyses.

Table S1

Human CNS coordinates (hg19) used in the gnathostome analysis

Table S2

Query sequences used
(A) Gnathostome analysis
(B) Teleost analysis

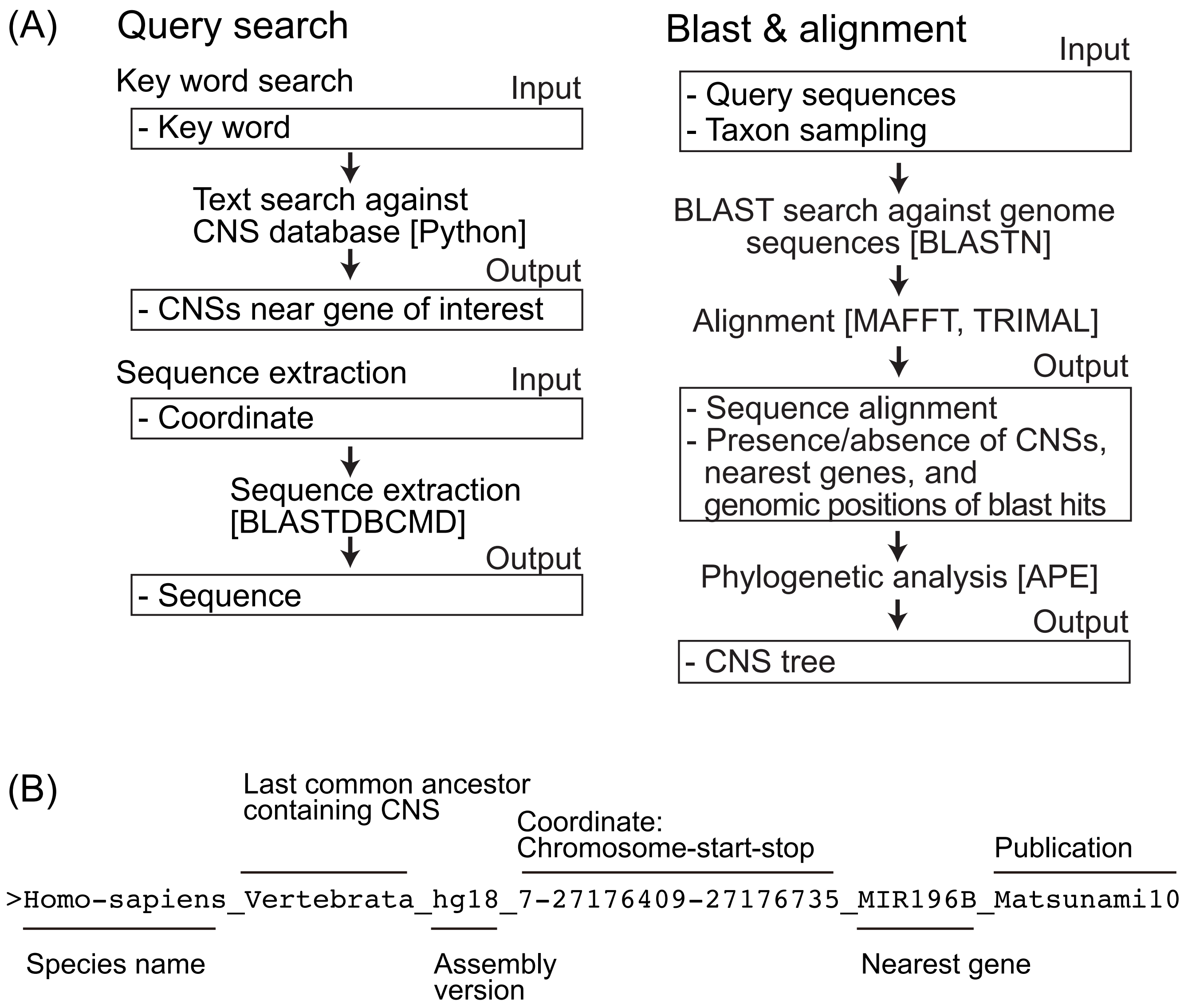
(A)
Query search
Key word search
Input
- Key word
Text search against
CNS database [Python]
Output
- CNSs near gene of interest
Sequence extraction
Input
- Coordinate
Sequence extraction
[BLASTDBCMD]
Output
- Sequence
Blast & alignment
Input
- Query sequences
- Taxon sampling
BLAST search against genome
sequences [BLASTN]
Alignment [MAFFT, TRIMAL]
Output
- Sequence alignment
- Presence/absence of CNSs,
nearest genes, and
genomic positions of blast hits
Phylogenetic analysis [APE]
Output
- CNS tree
(B)
Last common ancestor
containing CNS
Coordinate:
Chromosome-start-stop
Publication
>Homo-sapiens_Vertebrata_hg18_7-27176409-27176735_MIR196B_Matsunami10
Species name
Assembly
version
Nearest gene

Fig. S1

Your keyword: HOXA1

Last common ancestor containing CNS: NotSelected

CNS distance from the gene of keyword: Nearest from genes including keyword.

450 hits: Sorted by species names, databases, chromosomes, and starting positions.
Download tab separated file: result_queries2163.txt

>Canis-familiaris_DogHorse_canFam3_14-40347445-40347542_HOXA10_ANCORA
AAAAAAGCATGGGGGATCGTAAACTCGAACTTCGCCGGTTAATGGGCTTATTTATTGGTGCTGGCGGCTGCTTATTTTGGATGCCTTACAAAC
ATCC

>Canis-familiaris_DogHorse_canFam3_14-40347748-40347802_HOXA10_ANCORA
GGACATGAATTTTTACTGCGTCCCCACGCCCAAATATTAAAAAGCAAGTTCACA

>Canis-familiaris_DogHorse_canFam3_14-40348900-40348954_HOXA10_ANCORA
TTCTTGTGCAAAACATGCTGAATACGATTAGCAATCCCCCCGCACCGCGGCGGG

>Canis-familiaris_DogHorse_canFam3_14-40348955-40349043_HOXA10_ANCORA
GCCCGCAGCCAATCCTGAGCCAGAGTTTCCGCGCGACCACTCCCAGTTTGGTTTCGTAGGCGCGGGGCCGCTCTCCGAGGGCGCCCTC

>Canis-familiaris_DogHorse_canFam3_14-40354748-40354844_HOXA10_ANCORA
GCCCAGCCCGCTGCTAAAGAGATCTCATTTTTACATCTAAGAAATTGCTGCAAAACCCCAGCCGGGTTTATAGCGGCGCATTCCAAATATGCA
AAT

>Canis-familiaris_DogHorse_canFam3_14-40354853-40354930_HOXA10_ANCORA
CCGGACGGGTTTACGACCACATTGTCACAGCCATCGGAGGATGGGCTTTTATAGGGCTCAGAAATCAAACCCGCGCC

>Canis-familiaris_DogHorse_canFam3_14-40355330-40355399_HOXA10_ANCORA
GCCCTGCCCCTCAAGGTTATGGGTGATGTCCAAATTTAAGGCAGAAGTTCAAAGGCAGGAAACAAAGAG

>Canis-familiaris_DogHorse_canFam3_14-40358763-40358823_HOXA10_ANCORA
CTATCAAAGGCTCCCGGAGCCCTTGCAGGGGGAATTTACAGGCGCCCACCTCCGGCTCCC

>Canis-familiaris_DogHorse_canFam3_14-40358956-40359040_HOXA10_ANCORA
GCAGGCATGCCTTGGCCGGTGGGTATTTCACGGCCAATTTCAGCACTCGCCACGTGATCCCGCCTTTTATAACAAAGTTTTGTT

>Canis-familiaris_DogHorse_canFam3_14-40359041-40359119_HOXA10_ANCORA
GGGGAAACCTAAAGGCCCTTCATAAACCTTATATGCTTATAAAACAGCATATAAAAATTTAACAGCGGTGCTGCGCTA

>Canis-familiaris_DogHorse_canFam3_14-40359991-40360077_HOXA10_ANCORA
CGGATCCGTGAAGTAGAAATTTGGAGACGTAAGCTGACGTGGAAATCTATCCCCATCCTTAGCAGGGAGGTGCTGGTCATGTGACC

>Canis-familiaris_DogHorse_canFam3_14-40360249-40360368_HOXA10_ANCORA
GTAGCCACCTCAGGGGAAGCAACAGATCGTCACTCGGTGTTCTCACCGAAAGCACGTAATCGCCGGTGTAACTCATGTTGGCTGGGGGGCCTC
CCGGCGCGCGGCGAGGCTGGGGTGCG

>Canis-familiaris_DogHorse_canFam3_14-40360401-40360455_HOXA10_ANCORA
TCCTGGCCCTCAAGTGCGCCGAGGGCGGTGAGAGAGCTCAACTCCTGCCACCCA

>Canis-familiaris_DogHorse_canFam3_14-40360708-40360782_HOXA10_ANCORA
CTTGATTACACGCTCGCATTCATGGTCACTTCCGAAGCGCTTTAGTGCCTTCCGTCCCTAAACCGCCAACAACC

>Canis-familiaris_DogHorse_canFam3_14-40361629-40361700_HOXA10_ANCORA
AAGCTGGATGTCGCTGAAGGAAAGTCGAAAAATGAAAAAGGATAACCTTTTCCCTTTCCCCATCCAGGAAA

>Canis-familiaris_DogHorse_canFam3_14-40361723-40361836_HOXA10_ANCORA
TGGGGAGATTTAGAGTATAAACTCTAAGATCTTTGGTATTTAAGTGTCAACATCGATTTATTTATTTATTGCTGAGCTGACTGTAACTGACTC
AATAACAAATCTAATCATGT

>Canis-familiaris_DogHorse_canFam3_14-40362032-40362089_HOXA10_ANCORA
CTCGGACACATTGGTTTAATGAACTCTTTATTCAGGATTTGCTGCAAGAACTTTCAT

>Canis-familiaris_DogHorse_canFam3_14-40362522-40362634_HOXA10_ANCORA
ATAACAAAGGCGCCACCATTTCGGGGCTTGCCCCGCCCCTGGGTGAAGGCAAACAAATTCTTGCACTTGTATTAGGGCTTTTAAGACTATAAT
TGAACCCGGGGGCGTCTAG

>Canis-familiaris_DogHorse_canFam3_14-40362639-40362729_HOXA10_ANCORA
CCGAAAACAGTTCTAGACAGACCTGGGGTTTTATAGCAGTTTTGGCAGTCAACTTCAGCTTGTGCCTGAGCAGACCGGCTGCGGTGGCCC

>Canis-familiaris_DogHorse_canFam3_14-40363716-40363789_HOXA10_ANCORA
AGGAAGAAAAACAAAGGCAGGAAGTGGCGGCGGCCTGACGGTGCGTGTGTGTCTGCGGAGAAGGGAGGGAGCC

>Canis-familiaris_DogHorse_canFam3_14-40368773-40368823_HOXA10_ANCORA
GGGCTGCTGACACGCATTCGATCAACTTTAAAGGAATGCGCATAAATCAG

>Canis-familiaris_DogHorse_canFam3_14-40369875-40369947_HOXA10_ANCORA
GAGGCTCAGAATATTTCTTACATCTAAAGAAAAATATCCCCCGTCAACAGAAGAGTCCCCTTTGGAGCTGTT

>Canis-familiaris_DogHorse_canFam3_14-40370079-40370129_HOXA10_ANCORA
TTGATCTGAAGCATCATTTTCATTTGTTTCCACAAGTGGAGTGAAAATCC

>Canis-familiaris_DogHorse_canFam3_14-40370826-40370882_HOXA10_ANCORA
GAATTTCTGAAATGTAACTCTAGAACTCTAGAGTTAGCAAGGCATCTGAAACTTTT

>Canis-familiaris_DogHorse_canFam3_14-40371419-40371522_HOXA10_ANCORA
AGGTGGCGCCCTTGATCTACTAATCCAGCCGAGGCCAATTCATGAGCTGTCAAAAGTCAAGGTCACAAATTGTCTTTTTTCGTCAAGGTCAAG
GTTTAAGGCC

>Canis-familiaris_DogHorse_canFam3_14-40377550-40377671_HOXA10_ANCORA
GAAAAACGAAAACACAGCTCGGGGAGCCCCCACCAGCCGGCGCCTGTGCCAGCTCACCTCTGGCCATGGCGCAGCTGCTGGTGCACACAGCGG
CCAAGGCCAGCTCCACATTCCTCCCTCC

>Canis-familiaris_DogHorse_canFam3_14-40379069-40379122_HOXA10_ANCORA
TTTAATCGAGTTTGATTTTCACTAGCAAATGAAATTTACCTGCTCTCACTTTA

>Canis-familiaris_DogHorse_canFam3_14-40385603-40385655_HOXA10_ANCORA
TGCAGTTGCGACCTCTTCCCACTAGATGTCTTCCCAGTTAATCGGAATTGAA

>Canis-familiaris_DogHorse_canFam3_14-40391371-40391474_HOXA10_ANCORA
ACCCTCCCCTTAATGAACAGAGCTGTCGAGAAATAATTTCACTTTGATCTAGTTTGTGACCTGATTTTTTGGCTTAAAGAAGACTGGGCGGCT
AATTGGATTC

>Canis-familiaris_DogHorse_canFam3_14-40391475-40391558_HOXA10_ANCORA
TCAGTTGCCTTTAATCTCCACCTCAACACTTGGAACCTGACCAAGCTGAATTAAAACAAGTCTGATAAACAAGATGTAGCTGG

>Canis-familiaris_DogHorse_canFam3_14-40394100-40394161_HOXA10_ANCORA
TCAGTGGCAGCCAGAGACGTTCTCCCCAGGTTTCCGGCCCTGCTGGTGGCCCCTCCACCAG

>Canis-familiaris_DogHorse_canFam3_14-40394162-40394233_HOXA10_ANCORA
CTCCATGCAGGCAGGAACCCCCCAGAGGAGGAAAGGGGCCTGTTATAGGAGTCATCAACCTGTCACCACGG

>Canis-familiaris_DogHorse_canFam3_14-40418147-40418286_HOXA10_ANCORA
CTGTTTTCACACTCTCCTCCCCATTCGAGCGAGGCCCACACCTGGCGCATCACTGCCGAGCCATTAGCTGCGGGTCTCCTTTCATCTTCGCTG
TGGCAGACGTTTCTATTTATCCACTTGCGCTCGCCGAGTGGCGTCA

>Canis-familiaris_DogHorse_canFam3_14-40418320-40418374_HOXA10_ANCORA
ATGACAGCTTAGAAAGAAGAGGGCAATGGGGCTTCCTCCCAGAGGCGGTGCGGC

>Canis-familiaris_DogHorse_canFam3_14-40419453-40419578_HOXA10_ANCORA
GGGTGCTGCGGCCCTCCGACCAGTTTGCACGTCGGTCAGAGGTCCAAATTACCTTGTCACTTCCCGGGCTCGGTGGCGCCAGGTCGGAAATGG
TCCCAATGATCTAATTGCCTTTGGTCTCCGGT

>Canis-familiaris_DogHorse_canFam3_14-40419613-40419671_HOXA10_ANCORA
TTCCCCTTTCCTTCCCAGTCCCACTTCTCCACCCAAAGGAAAAGGAGCTGCAGGGGGC

>Canis-familiaris_DogHorse_canFam3_14-40424430-40424714_HOXA10_ANCORA
AAGATTGCTTGCGACAGGTAAATGGGCTGCGGGTTGCCGTAATGCCAGGCGTATTTTCAGTTAATAGGGAGGGAAAATGAGGTGTCTGCAGCG
ATATTGGAAAGTACAAGATCGATGATCCGGCCTCGTGTCCAATCAGCAGCCTTTAATTGACTGTATTTTCTGGGTAATTGAGCCTCTCTTCCT
CCAAATTGAAGTGGAAGATATAACAATACATCTTGCCAGGAGGAATTACTCATTACTTCATAATGAAATTTCCCTTTTGCCAAGGTTTGCGGT
TTGGC

>Canis-familiaris_DogHorse_canFam3_14-40425307-40425436_HOXA10_ANCORA
GTGATTGATTGCCTTATTAAAGCGTGTTCTTGTAAGTGTGACCAAACTGATTGCATTGCATATGTTTGGGATAATGCTCATTTTAAACAACAG
GATAAAGAGGATGAGCTCGGCAGAGCCTCCGAAGAC

>Canis-familiaris_DogHorse_canFam3_14-40426707-40426833_HOXA10_ANCORA
GCATTAAGTAATTATTTAAAACAAGGCTGTTTAGAAAAATATTTGTATTTATTGCAGCTGCTCAATTGGCCTCGTTAAAGTCGGCAAGCATTT
AAATTGTGTTACAATCTCATTTAAATCCCGCTC

>Canis-familiaris_DogHorse_canFam3_14-40426858-40426966_HOXA10_ANCORA
ATAGACCAATCACTTCATAATGATGATGAATGAGAAATTAATTCAGATACAAGCAAGACAATTTAGGCCTTCATCTGTTTAAATAGCCTTCCA
ATATTATTCGCGATC

>Canis-familiaris_DogHorse_canFam3_14-40427001-40427095_HOXA10_ANCORA
GAAAGTCATATCCTTGCATCTTCATCGAGATTATTTATAGCGCAGTGAGGGCTGCTGAAAGGTATACGCTGTTAACAGGGACAATTACTTAAA
G

>Canis-familiaris_DogHorse_canFam3_14-40428402-40428473_HOXA10_ANCORA
CTCTGAGGACCCACTCCCGGGAGCGACTTTGATGTATTGCTGTCCAATTAGTGCCTTTAATTGGTGTCCTC

>Canis-familiaris_DogHorse_canFam3_14-40428806-40428877_HOXA10_ANCORA
CCCATGTGGTCTCTCATCTCGTTAATAATGGCTGTTTGTTAACAAGGATGCGGAGGTTAAATAATCCGGGT

>Canis-familiaris_DogHorse_canFam3_14-40468120-40468176_HOXA10_ANCORA
CCCAAAGAATAGAGGCCAGTGTTTTATGTTTCAAGTGTACCATATGGCATGATATG

>Canis-familiaris_DogHorse_canFam3_14-40472579-40472630_HOXA10_ANCORA
CTATTCTGGGGCTCATAAACCTTTCATCAGAATCAGAGGTGAAATATTTGG

>Canis-familiaris_DogHorse_canFam3_14-40478152-40478209_HOXA10_ANCORA
AAGAAGTTGCAGCAGGCAAAAAGAACAGCTGGATCATGCATCTTTCAAATGGGAAAA

>Canis-familiaris_DogHorse_canFam3_14-40517223-40517333_HOXA10_ANCORA
GAGGAGAAGTTTATAATAGGATCAGTTTTGTTTGCAATTTGTTTCTAATAGATGCCAATAAGCCAGTTAACTAGGCTCCAGTTAATGGACATT
TTATTGTTTCTCCCCCC

>Canis-familiaris_DogHorse_canFam3_14-40517466-40517723_HOXA10_ANCORA
CTTTGATCATCCTGTCAGTGGGAAATTTACAGCAACATTCGGGCTTTTGTTCCATGTGTATTTCTCCAAATATAATATCTCCCTCAAGAAATC
AATGATAAGCTGTTAAACTAAAGAGCATGGTAACATCCACAGAGCAGTAAATTAATGGTCTTTTGTGGATAGATGGTTACAGATTTCATCATA
AAAAATGGATAAAAGATTACATATCACGCAGAGCGAAACTTTTAATAACTCAACATGACTTTTCTTAATGG

>Canis-familiaris_DogHorse_canFam3_14-40517724-40517825_HOXA10_ANCORA
TTTTATTCATTTTAATCATTATAGTGTCAATGATGATTATAGCCTTATTAGATTGTAGTGATTGAGGGGCCTGTTTATGAGATATTGAAAATC
TCAGGCTG

>Canis-familiaris_DogHorse_canFam3_14-40517876-40517967_HOXA10_ANCORA
GGAGTAGGGCTTGTCAGGAGAGTTTCAATTAATACTTTTACTAAATTGCTCCTGTCTTTAGTGACCTAGGTCACAGATGACTTAAGGATTC

>Canis-familiaris_DogHorse_canFam3_14-40521546-40521610_HOXA10_ANCORA
TCTTATAGCCTTTTACATGTTCCATTTTTCAATTTTATCTATTGGAATGTAAAATTTCAGCCCT

>Canis-familiaris_DogHorse_canFam3_14-40525858-40525909_HOXA10_ANCORA
GTCCAGGAGATAATTGATTCTCCAAATTAGTAACTCAATTGGTCAAAATTA

>Canis-familiaris_DogHorse_canFam3_14-40525910-40525961_HOXA10_ANCORA
AATACCTGTTGAAGTTACTTTAAATTATGCTCTAATAGTAATTAGAGGCTT

>Canis-familiaris_DogHorse_canFam3_14-40526205-40526264_HOXA10_ANCORA
CTTGATAGGTTCATAGACCCATCTTTTTCTTTTACTATGGCAATAAGTAATTGTGTCT

>Canis-familiaris_DogHorse_canFam3_14-40527308-40527363_HOXA10_ANCORA
CCTGTCTGGGGCCTGTCTAGACCTCAGAATTGGCCTGGTTTGTTTTAAGGTTTCA

>Canis-familiaris_DogHorse_canFam3_14-40534686-40534780_HOXA10_ANCORA
CCCAAGTCTATTTTAAAGCACTTTCCATTTTAAATATAGCAATAAAATATCAGCCTCGAAATGTCTGATAAGGATAAAAGCAATAGATCCAAA
T

>Canis-familiaris_DogHorse_canFam3_14-40534837-40534959_HOXA10_ANCORA
TTTATGGGGCCATAAATGGGTGGGCCAGCTATAATTTGGGTATTAAACTTGTAAAATATATTGATGGTATGTAAGAGCTAAATAAATAATTTA
CTACAAGAAAATTGGAAATCTTTACATGG

>Canis-familiaris_DogHorse_canFam3_14-40550722-40550824_HOXA10_ANCORA
TGTGAATGCCTTGGGTAAAATGCTCATTAAACTGTCAGCTAGCAGAGAGGTGTTGCTCCTGGGAAGAGGGTCACAGCATCCTGGGGTGGAAAG
AAGCCTCCC

>Canis-familiaris_DogHorse_canFam3_14-40557758-40557830_HOXA10_ANCORA
AAGAAAGTTCAATCAATGGAAGGGTCAAATGTTTTCTTTTTCAAACGTCCATTTTGCATGTAATGGCTGCTC

>Canis-familiaris_DogHorse_canFam3_14-40561707-40561807_HOXA10_ANCORA
GCTGCTCTTAATAAATGTCTAGTTAAGGTATAGTTAATATAGTTAAATGTGAAAGTGTATAAATGATATATTTTAATAGCATTCAGAGCACAT
TTCAGCA

>Canis-familiaris_DogHorse_canFam3_14-40561866-40561923_HOXA10_ANCORA
AGCAAATGATAAATTACTAAAGAAAAATGTTATTCTGCAATGTTTTGAAGATTAAGT

>Canis-familiaris_DogHorse_canFam3_14-40562024-40562090_HOXA10_ANCORA
CACAGAAATGGAAGTAGTTTTCATTTTAATTGTTTTTCTTTAAATGGATTGTTTCTGTAATATACT

>Canis-familiaris_DogHorse_canFam3_14-40570544-40570599_HOXA10_ANCORA
GGGGCAATAATCGTCAAAGTAATATTTACCCCTTAATAACTGCCTATTTTTATAC

```
>Canis-familiaris_DogHorse_canFam3_14-40570621-40570738_HOXA10_ANCORA
TGTTATTTACTCCTCTGCCAAAAAGAACTGAAGGATGTGAAATGTAGCAGTCAATAAAGCTGAACAGTCAGATAAATAACATTCATATTATTT
CCTGACAAATCCTTCTGCAACCCA

>Canis-familiaris_DogHorse_canFam3_14-40570753-40570810_HOXA10_ANCORA
CTAAGAAAGCAGTTCCTGCATTACAAGTTTACTGATGTGTAAAATGTAATTTTAAAA

>Canis-familiaris_DogHorse_canFam3_14-40576979-40577050_HOXA10_ANCORA
ATCTTGTGGGAGAGGTTGAGAGGATTCTGTTTAAAAAGGCTGCCCTAATAAATGATTTACCAGGGCAGCCA

>Canis-familiaris_DogHorse_canFam3_14-40577114-40577174_HOXA10_ANCORA
CCCCAGAACCCCAAACCTTTTATGACTTTTATATTAGCTAACCACTAAATTTTCAAAGCA

>Canis-familiaris_DogHorse_canFam3_14-40586807-40586881_HOXA10_ANCORA
TCTTATAAATAGAAGGTAATTTCTAAAATGTCAAGGGTTTCTCGGCTCAATTAAAATTATTTGTAGCACAAATG

>Canis-familiaris_DogHorse_canFam3_14-40591172-40591273_HOXA10_ANCORA
TCTCATCTTTGAAATATAGATTAGCCAGGAGAGTATGAGTTCAGCATTTCTATGTAATGTTGTTTCATTGATCCTGCAGGCCAACTGGACATG
GCTTCAGA

>Canis-familiaris_DogHorse_canFam3_14-40591314-40591364_HOXA10_ANCORA
ATATTTTATAACCTGTCAGAAAATCAATTATGGACTCTGCCCCCTTTCAA

>Canis-familiaris_DogHorse_canFam3_14-40591436-40591510_HOXA10_ANCORA
AAGTTCAGAGTGAATGATGACTTCAGGGAAATTCTTGGATTTGTAAAGATTTAGAGATGAATTGAATAGCTTTG

>Canis-familiaris_DogHorse_canFam3_14-40594517-40594668_HOXA10_ANCORA
CATCTCGAGGCCTACCCTGAGCCAATCACTGGAGAATGGTTTAATTGGTTAATCATATAAATTACAATCACTTAATCATTTTAATCACCATCA
GCCAATTAGCTCAGAGTTATGGGCTCTGAGGTAACAGTTATGCAAATGTTTGTTCCAC

>Canis-familiaris_DogHorse_canFam3_14-40606287-40606369_HOXA10_ANCORA
TATCTGGTTCTCAATAGCAATTTGGGTTTTTGGAAGTTATTTTACAGTTGTAATTACAAAGGGAAATGAAGTCTGCTTTTGC

>Canis-familiaris_DogHorse_canFam3_14-40607470-40607555_HOXA10_ANCORA
TCCCAAGTATTTCAGAAACTCTACACCTCTGGTGGCCTGAGCAAGAAACCACTTGCAAAGAGTTGGGTTGGTGTGCTAAGAGCTA

>Canis-familiaris_DogHorse_canFam3_14-40607569-40607625_HOXA10_ANCORA
GCAATAAACAAATAGTAAAGTTGTCTTTTGCTAGTCAGTACAAAACTCCCTCTCGC

>Canis-familiaris_DogHorse_canFam3_14-40607626-40607708_HOXA10_ANCORA
GCTCTAAAAACCATTTGGAATTGGTCCAGCTTACATATCTGTTGGATGTTTAAGAAATAGATTAAGAGGTCAGCAGGGAAAA

>Canis-familiaris_DogHorse_canFam3_14-40618798-40618856_HOXA10_ANCORA
TAAGATTTTAAAATCTACCCCTTCCCCATCAATCCCATAATGCTGAATACAATGCTCA

>Canis-familiaris_DogHorse_canFam3_14-40618990-40619042_HOXA10_ANCORA
GGACTTGGCTTTCAACTTCTTTCATTTAAGCCTTAACAGAAACATGAATCTA

>Canis-familiaris_DogHorse_canFam3_14-40623583-40623637_HOXA10_ANCORA
TTTTTATGTCCAAGTTTTAGTAACAGGATGTTTTCAAATAGTTTACATTTCTCT

>Canis-familiaris_DogHorse_canFam3_14-40633238-40633295_HOXA10_ANCORA
AAGAGTAACAGCAGGAATCATGACTGCAAGGAAACAGTTACCATCCTATAAAATTAT

>Canis-familiaris_DogHorse_canFam3_14-40633603-40633653_HOXA10_ANCORA
AGTAAAATATCATTGCACCTACCTCACTGATCAATAAAACAAAATAAAAA

>Canis-familiaris_DogHorse_canFam3_14-40633660-40633730_HOXA10_ANCORA
TCTTCCATTTGGATGAGAGGCAATTTTCTGTGGCTTCTCACATTTGCTAGAACTTTACAAATATGTATCC

>Canis-familiaris_DogHorse_canFam3_14-40646555-40646620_HOXA10_ANCORA
CTTTGCAATCCCTTTAATTAACAGTTTGTTTTTATTTTTCAATATTTGGTGGTCGATTTAGTTGA

>Canis-familiaris_DogHorse_canFam3_14-40663658-40663714_HOXA10_ANCORA
GAGCAAGCAAAACAAGTCTTAGGTCATCATTTTCCCATTAGCTGAGGACAGCAAGG

>Canis-familiaris_DogHorse_canFam3_14-40663719-40663789_HOXA10_ANCORA
CTTTATATGAATATTCTTTCCCTTTCCCACAAAGTAATTACCTAAGATTTCTAGCTAGAAAAAAAAAAAA

>Canis-familiaris_DogHorse_canFam3_14-40663858-40663929_HOXA10_ANCORA
CCTATGCAAATCAGTCCTGATGAGCCCTTAACTAATCTGTTTACAAACCAGACCTGCTAGACAGGCCTCAG

>Canis-familiaris_DogHorse_canFam3_14-40664038-40664147_HOXA10_ANCORA
```

CTCCTAAATGAGTGCATTATTTCCACTTACTACCTTCTTGTTTGCAATTTGTGTTTATTAACAGCACTGTCTCAGGTTTACTTTGTGAATTAC
TAGTTTCTGAAAACAG

>Canis-familiaris_DogHorse_canFam3_14-40664174-40664230_HOXA10_ANCORA
CATTTGTGGAGATTATGTAGCATATAATACATAGAATGGCCCATCAGATAGGACAT

>Canis-familiaris_DogHorse_canFam3_14-40666640-40666690_HOXA10_ANCORA
GCTCATTCTAGATGAGCTCTCTTGCCATCTCTTTACCAGGCATGACGGAC

>Canis-familiaris_DogHorse_canFam3_14-40678497-40678548_HOXA10_ANCORA
GAGCTTAAGAGAAGTTATCATTATAAAAGAAGACATTTCTTGCTGCAAACT

>Canis-familiaris_DogHorse_canFam3_14-40678609-40678719_HOXA10_ANCORA
TGTGACATATGCTATCTATTCAGAGACATGCATTATAGTTCTGGGTCAACTGCTGATAAGGTTACTGATCCTGATAAAACAAAGCTTGCCCAA
GCGGAAACATCATTTGT

>Canis-familiaris_DogHorse_canFam3_14-40680346-40680397_HOXA10_ANCORA
ACTCCCCACCCTACAGCAAAGTGTATCATTAAAAAGAGCAAGTTAGAAGTG

>Canis-familiaris_DogHorse_canFam3_14-40680510-40680767_HOXA10_ANCORA
AAGGCTTGTAATGGTCTGAAACCAGCTAGAATACACAGTAATTGTCATTCAGAGAGTCTATCCTAGAACGTCATGCTTCACTGGAGAAGACTG
AGTTTCCATTTGAAATCAACAAATTAATCCAAGACAGGTATCTGAAAGGGCAAGTTTAATAGGAAGCAGCTACTGGGCTCTCAACCATGATAA
AATGTTATTGCTGTTTCCCTGTAAATTGGCTGGGAACATATGATACTTTCTGCATAAGAGCCCCATTGCTG

>Canis-familiaris_DogHorse_canFam3_14-40680768-40680858_HOXA10_ANCORA
TCAGGAGATATACAGCTCAAGCCATCAAAAGGATGACAGTCTGTCAGTCCCCATAATAACTTGCCACTGGATGTGGTTCGAGAGTGTGCG

>Canis-familiaris_DogHorse_canFam3_14-40682976-40683141_HOXA10_ANCORA
GTACTACTTTGTGGGAATTTACTACTTGGGAATTTATGCTTGCTGATGCCAATTTTCTCTTTCACCTTGCAAAGCTCAATAAATCGTGGCCAC
TCTCTATACTCTATTAGAGAACAATCCTGTCATTTTAAACCATGTCTGTAAACCATGGAGAAAAGACAATAG

>Canis-familiaris_DogHorse_canFam3_14-40684300-40684393_HOXA10_ANCORA
CAGACATTCAATTTTAAAATGTAAATGGTACAGGATATTGATAAACCAATTAGCACTGATGCAAAGCAAATTCCTCTTTTATCTCAATTACAA

>Canis-familiaris_DogHorse_canFam3_14-40684394-40684448_HOXA10_ANCORA
ATGACAACTTAATAGGTCAAGATAGTTTAACATCTGAGACAAGGGCAATTCAAT

>Canis-familiaris_DogHorse_canFam3_14-40684448-40684506_HOXA10_ANCORA
AAACAACAGCATCTTGATATGGTAATTAAGAGGAAATGTGAATCCATTACTGTGTTTT

>Canis-familiaris_DogHorse_canFam3_14-40689684-40689763_HOXA10_ANCORA
TAGACGATTTAAGTCAAAGCTTACAAACAAATCCAGCACTATCCATCATAGGTCAATAGCTTTGTATCTAGATTACAGA

>Canis-familiaris_DogHorse_canFam3_14-40689808-40689871_HOXA10_ANCORA
CTGACAATGGTTCTCAGCTGGTTAATCTTTTGACAGGTGTACTTTCTCACTTGTTCAAACACA

>Canis-familiaris_DogHorse_canFam3_14-40696393-40696488_HOXA10_ANCORA
AGTAAATCACTTAATTTACTCAAAAGAACTTAAATGTTTTAACCAGTCTGCCATCAAAAGAGATCAAGAAGAGGACCAATGGGACTTGGGCTG
CA

>Canis-familiaris_DogHorse_canFam3_14-40696489-40696620_HOXA10_ANCORA
TGTGAGCCGTCTTTAAAATGCAATTGTTCTAATCATTTTCTACAGATGCAGCCTCACAAGGGGCTGCTGTCACAGGGACCATTTACGACTTGT
TATGGTGTCTCTATTGTTTACCAGCGACATTCGCTGAA

>Canis-familiaris_DogHorse_canFam3_14-40696621-40696881_HOXA10_ANCORA
AAGGCGACAGCTGGGACAGAAAGCTGCTACTCAACGAACAATATAATCCCGTGAAAGTAATCAACAAATTTAAGGGTGTTAATTAACTTTACA
CTTTATCATGTTCAATTAGTTGTCCTTTCTAGAAAAGATTGCCGTGCACAATCAGTTTAGAGCGTGCCATGTTTGATTTGTAAGTGATCACTG
TTTCTGTTTCAGAGTCCATGTCAGATGTTCTCTTGGGTTTTGGTGACACAGATGAATGGCTCTGCACATGTTAG

>Canis-familiaris_DogHorse_canFam3_14-40696882-40696950_HOXA10_ANCORA
GGGTGATCTGGTTTAACATAAGCCAGCATAACATAACCCCTTGTTAACACTATTTCTGGCATTATCCA

>Canis-familiaris_DogHorse_canFam3_14-40697075-40697160_HOXA10_ANCORA
ATTATTTTTCAAAGTAAGACTTGTCTTTCAGGGGCATGTTTGGGATCATTTCCTCCCAGCAAGATCTCGAAGCTCTGCTGCATTA

>Canis-familiaris_DogHorse_canFam3_14-40697959-40698136_HOXA10_ANCORA
TCTATCCCAAGTTGGCAGCTGCTACATTTGCAGACCTGGTTCATTATTTACCCATAATGACCATCAAGCTGCAGTCAAAGTCAGATCACAACA
ACTGTCAGCAGCTCTTTTAATTAGCCTTGTTTGAATTCAGATAAATATCACAATAGACCTGCTTATGCTGAAAGAGCAACAGAA

>Canis-familiaris_DogHorse_canFam3_14-40712921-40712980_HOXA10_ANCORA
ATGCCAGGATATCTTTAACTCTTTTCTGATAATAAAACAGGTTTCATCTTTACCATTAG

>Canis-familiaris_DogHorse_canFam3_14-40717996-40718055_HOXA10_ANCORA
CACAGTTAACTTGTCATTGAATGATTAAATATGAATTCATAATTAGAAAAATCCACACT

>Canis-familiaris_DogHorse_canFam3_14-40718065-40718128_HOXA10_ANCORA
GGCTTTCCTGACTGTTCTGGTTCATTAAAAGTTGATTACTAAACAGTGACCTCTAGTGGCCCA

>Canis-familiaris_DogHorse_canFam3_14-40719205-40719279_HOXA10_ANCORA
AAGAACCGAATCACCATTTAATGCTATTATCAACAGTTCATGACCCACCAGCAAGAAATGTCAACCTGATTAAT

>Canis-familiaris_DogHorse_canFam3_14-40728351-40728424_HOXA10_ANCORA
TGCAGTCGCCAGGGTCCAGCTGCCAGTCTGGGCGGTGCTGTCCATGCTGAGCAACGTGCCACCCAGCTGCTGC

>Canis-familiaris_DogHorse_canFam3_14-40728425-40728489_HOXA10_ANCORA
TGGTCACGTCCATCTGGAGCTCTCTAGCTAGACAAATCTCTATGCCTGTCAGATCTCTTCAGCA

>Canis-familiaris_DogHorse_canFam3_14-40728490-40728575_HOXA10_ANCORA
GCCTTGTTATGCAAATTCAATTACTCAAGTGTGCCCTATTACTGCTGGCTAACTGCTCCTAGAACCAATCTCACACTGACAGTCA

>Canis-familiaris_DogHorse_canFam3_14-40728576-40728643_HOXA10_ANCORA
TTCACATTTTTTGCTCACCAAAAAGTACCTCATTTATTCTTGCTTTGCTATTCCCTTGTATCATTAA

>Canis-familiaris_DogHorse_canFam3_14-40728782-40728836_HOXA10_ANCORA
CACTTGACAGAGATGATTTTACTACCAGAAATAGGAAATTTAAATCTTAATGAC

>Canis-familiaris_DogHorse_canFam3_14-40739464-40739647_HOXA10_ANCORA
AGGACAACTCCATCTGCTACAACAAAACAGAGAAAGATAAAGGTCTCCAAACACTTAATAGCACTTTCCAAAAAAAGGGCCATAAACCTAACA
CCACTGAAAGAGAAATGCAATCTGCACTTTGCAGACCTAGACAGGAATTGCTTCAATACTATCTTTTTCCCTCTTCCTCACCATAAAAAC

>Canis-familiaris_DogHorse_canFam3_14-40745771-40745930_HOXA10_ANCORA
GCTCACAAAAACCCTGGTGAGAAATTCTAACAAAACAAGCCAAGAAGCAATTCATGGACAGCAGGAGGCAATTCCAGAACACAGGCACAATCT
ATAAAGAGAAACCACTACAGCTTTTTGATGTTAAATAAGTAGAAATATCAGCATACAACAAAGCTA

>Canis-familiaris_DogHorse_canFam3_14-40747557-40748030_HOXA10_ANCORA
CTGCTTTGTAAAATTACACTGTATAATTAATTCCATGGTAAGAATGTTTTTATGGTGTGTTTTGGTGACCTTTTATTACCATCAAGTTCACAA
TGCTGGTGGGCTGCCTTTTAATCCCTCCGCAGACAAAGGCCCTAAATAAACACAGATCCCTTCCTGGGAAAAAGTCCACTCTGGTTAAATGGT
AAGGAAATGAGTAAATCATTAGTAATCTTTAAAGCCAAAGCTATAAACTCAAGTTACCTTAAGTTGAAATGAAAGTGTCCTGCAAGAGTTTCA
AAGTTCTGTCACAGATAGAAATCTCCAGCTCTTGACTCCAGACTAGGCGCCAGCTGATAAACAAACAATTTAAGTCAAGAGCTGGAGAATTCC
CAGCTTTTTCATTTAATGGCTTTGGCCAAGAGTATTTATTGCCTCCAGTTTTATTCTCACTTAGCTCTAAGAGGAAATTTAAACTCATCATTA
TCTTAAAG

>Canis-familiaris_DogHorse_canFam3_14-40758518-40758574_HOXA10_ANCORA
TCAACCTTCCCTGAAATCTAAACATTTATCTTATCAGTTTTCTGCCTAATATCCAA

>Canis-familiaris_DogHorse_canFam3_14-40792876-40792926_HOXA10_ANCORA
GATCCAGGCCTTTCTGCTGTGCCAGGATGCAAACTGAGAGGGCCACTGGG

>Canis-familiaris_DogHorse_canFam3_14-40813124-40813174_HOXA10_ANCORA
TCTTCCTACCATTGGCAAAGTAACACTTGTTAACAGTTCCCCTTGTGACA

>Canis-familiaris_DogHorse_canFam3_14-40813209-40813276_HOXA10_ANCORA
TTTAGCACTTACGGATATTTTGAAATCTCTAATGAATTTCTTTTTATAGACATTTTTACGTTTTACT

>Canis-familiaris_DogHorse_canFam3_14-40923094-40923151_HOXA10_ANCORA
CATGACCTGTTTCTCATTTAATCTAGTTTGCTGCATTTAATTGCTATTAACCTTTGC

>Canis-familiaris_DogHorse_canFam3_14-40923407-40923459_HOXA10_ANCORA
CTCTTCTGTAGTCATCTGCTTCAGTTTAATTAGCAGAATGTTCTTTCTGATC

>Canis-familiaris_DogHorse_canFam3_14-40939657-40939751_HOXA10_ANCORA
CTAATAAAAATAAAGAGCCATGTTTATTTTGGGTCAGAGGCAGTTTATTTTAAATGTGATTTCATTTACATTTATAAGCAGCTTTTCTTGACA
G

>Canis-familiaris_DogHorse_canFam3_14-40939762-40939856_HOXA10_ANCORA
AAGTTGCCTTCAGTTCTAAAACAAACCTCTGTTGCTTTCAAACTTAATGTGCTCTGTCCCCAACAAAACATATGAAAATATAATTTAAAGCAC
A

>Canis-familiaris_DogHorse_canFam3_14-40939857-40939963_HOXA10_ANCORA
TTTTCACAGACACAGTACAATACATTCACTATACACGGTATAACAAAATATTCCCATCCTTACCTGTTTAGTAATACTGTCACAATGAATAAG
TAAATAGAAATTG

>Canis-familiaris_DogHorse_canFam3_14-40940558-40940613_HOXA10_ANCORA
AGCACTTGGGAGAAAAGAACACTCAAGGCATGATGGTATTGTTGGTGAGAAAAAC

>Canis-familiaris_DogHorse_canFam3_14-40950632-40950685_HOXA10_ANCORA
TTTTTTAATGAGGCCCAAAGCCATTTATTTTGAGCTTTTGCACAATACAACCC

>Canis-familiaris_DogHorse_canFam3_14-40950686-40950740_HOXA10_ANCORA
GAAGGACATAATTCTACAGATCATAAACAGTGTAAAATAATATGTTGACAAGTC

>Canis-familiaris_DogHorse_canFam3_14-40950781-40950881_HOXA10_ANCORA
GCACAACATTAATAAGAATTATATGGTAGCAATTACAGTTGTGAAATTCATCCCCCAGGTGTGAGGCTAAATTGCACCAGGGGCCTGCCTGGG
TGCTCCA

>Canis-familiaris_DogHorse_canFam3_14-40956737-40956796_HOXA10_ANCORA
CTGCAGTGACAGCAAATTAAAACTCTCCTGGGGTGTCAAATTCTGGCAAATACATTAGC

>Canis-familiaris_DogHorse_canFam3_14-40962606-40962681_HOXA10_ANCORA
AGTTTTATGAGAGTACTTCTGATTTTTAAGAAGGCTATTTGGCTTCATAACAGGGCAGCTTCCTGAAAGCACCAG

>Canis-familiaris_DogHorse_canFam3_14-40967425-40967513_HOXA10_ANCORA
CAGGGATGTTAATTGAACTTTGGCAAACGTTTAAAGATTTCTCTCTCCATGTTCAAGAAAGAATTAAAAACAGTTGAGCTGGGGAAAA

>Canis-familiaris_DogHorse_canFam3_14-40967539-40967606_HOXA10_ANCORA
ACAACCACAATGAAAAACATTCATAATTAAAGCGATTTTGCTTCCCTGCTAGTTAGATCTGTAATCT

>Canis-familiaris_DogHorse_canFam3_14-40967710-40967764_HOXA10_ANCORA
TGGAATAACAATATCAGGAAACAGATTTGCGTAGAAAGATAGGGGATGACAACA

>Canis-familiaris_DogHorse_canFam3_14-40981529-40981579_HOXA10_ANCORA
CCCAGAGCAGGTCATTAGCAAATACCACCTGTAATGCACATTCCTGGAAC

>Canis-familiaris_DogHorse_canFam3_14-40987132-40987192_HOXA10_ANCORA
AAACCACATTAGTCAGGCAAAATAAGCTGATCTCTCTAACTACAGGTAAAATAACATTTC

>Canis-familiaris_DogHorse_canFam3_14-40987399-40987488_HOXA10_ANCORA
GATTTCTTTATGTCTGAAGGAAAGGAAATGATTCCATTTCTTCCAAACATTCTTACATGAAAAATAATAAACTCTCACTTTTAAACTGT

>Canis-familiaris_DogHorse_canFam3_14-40996605-40996746_HOXA10_ANCORA
AGTTACTTTAATTTAATATGGTCATTTGTAGTAATATTTGTTATTGCTTGGGGAAGTACATATTCCTTAAGTAAATTTTCAAGACTCCATTAT
AAATCTTTTAAATCCTTAGACCACAATGTTCATTCAGTCTGACATGTT

>Canis-familiaris_DogHorse_canFam3_14-40996750-40996801_HOXA10_ANCORA
GTGGAAACATGAATATAACACCACAGGGGGAAAATGTCAAACTGCTGGTCC

>Canis-familiaris_DogHorse_canFam3_14-41007437-41007503_HOXA10_ANCORA
TTTCAGTTGCCTGGGGGATTCTTCATGAAGTTATCTGATCCTGTATTTCTTACTGTGTAACCAAAG

>Canis-familiaris_DogHorse_canFam3_14-41010128-41010226_HOXA10_ANCORA
ATCCCTTCAAGCAGGCCCTATTAAAAAAATAATCACCATCTGCTAGACTCCCTTCTTTTTATTAAACTGGTCATTAATTACTGGTAACAACT
AATTT

>Canis-familiaris_DogHorse_canFam3_14-41010669-41010719_HOXA10_ANCORA
TTTTACTAATGTTTTTCAACTACAACATTTAGGTTAAAACATTTGAAACA

>Canis-familiaris_DogHorse_canFam3_14-41010830-41010887_HOXA10_ANCORA
AAATATGTTGACTTTATTATTAATACTCAATTTTGGTTAATAAGGCAGCTATTAAAT

>Canis-familiaris_DogHorse_canFam3_14-41018872-41019013_HOXA10_ANCORA
GTTTTAAATTTGTGACATGAAATCATGCCACTTAATTAAGTCTTAATGCACAAAAATATATTTTATTTACTTTTTATGGAATATCAGCATTTA
AAGCTTTCTATTTATGTGTGTAAACGCTTTATAAATTACTTGAAATAC

>Canis-familiaris_DogHorse_canFam3_14-41025585-41025680_HOXA10_ANCORA
CTATCTGGTTTCAGTTAGGCAACAGTCTTTGGCCAGCATTTTCTGGTACATTTGAAACTGTTGAACAATGAAAAGTAAAAACATGTAATTGCC
AG

>Canis-familiaris_DogHorse_canFam3_14-41025718-41025769_HOXA10_ANCORA
AGCACTTTATTTAGGTTCTGCTTTATTATTCTTAACTTTCATAGTAATTTT

>Canis-familiaris_DogHorse_canFam3_14-41026492-41026547_HOXA10_ANCORA
CAGCAGGAAGTGAGGGTCATTCTGGAATCTTCAATGAAAACACTCACTACAGGGT

>Canis-familiaris_DogHorse_canFam3_14-41032548-41032645_HOXA10_ANCORA
CTTCATTTTACAACCAACATCTTGTCCACATCAGGGGAGCTTTCTCACACAGACACGCATACCATGTGGGCTACCTGCTGAGTTCTGGCAAGC
TTGG

>Canis-familiaris_DogHorse_canFam3_14-41032646-41032728_HOXA10_ANCORA
AGACTGCAGAACATAATGGGAAGGCAGTTGGAGTGGAAAAAAAACCTTCCTGGGAATTTTTTAAGTGGTTAAAAAAAAAAAA

>Canis-familiaris_DogHorse_canFam3_14-41032748-41032803_HOXA10_ANCORA
ATGTCCCACACTGTGAGTTGGCACAGGGAAGATAATCCAGGAAATGAGATGTAAA

>Canis-familiaris_DogHorse_canFam3_14-41039758-41039855_HOXA10_ANCORA
TCTGACCTTTTACACTGGTTTTGTCAACAAAATTTACTGTATTCATAAATACTAATGTTCTCATTTGGTTCCCTATCTTTAGTATACTGTAAA
TTAT

>Canis-familiaris_DogHorse_canFam3_14-41039876-41039951_HOXA10_ANCORA
GGCCTGGTAATATAGTGGCCTGAAATGACTTCCTTTTTCCTTTAAAGATGTTGCATGTTTTAATGGTAGGTGTTT

>Canis-familiaris_DogHorse_canFam3_14-41040028-41040078_HOXA10_ANCORA
ATCTGACTAATGTTAACAAATTAGGCATGCCTTCAAGATGCACAATTTCT

>Canis-familiaris_DogHorse_canFam3_14-41040285-41040349_HOXA10_ANCORA
CCAAATTAGCACTGTAAATATTGAAATCAAATGTTACTACTTGGCCTAATTTTCTTTCAGCCTG

>Canis-familiaris_DogHorse_canFam3_14-41040350-41040454_HOXA10_ANCORA
AGTTAATCTTTATTCAGCCTTAATGAGTATTTATTGTGTGACCCTGACAGCTTGCTACTGCTGTGATAAATTATCTGACTCAAGTCCGATTAA
AAATGCAACCC

>Canis-familiaris_DogHorse_canFam3_14-41040455-41040546_HOXA10_ANCORA
GAGTGCTAATTATCTCAACAGACCTGCTTAATAATAGCATTTCTTGTCAATTATGGAATCTCAGCATGGGTGTCAATAGCAACAAATGCAC

>Canis-familiaris_DogHorse_canFam3_14-41040608-41040682_HOXA10_ANCORA
GCTTCTGTACCTGTGGTGACCTTTTATTAGCAAACTGTCAAAATGATAAAACTAGCCATAGCAAAACCCCCACC

>Canis-familiaris_DogHorse_canFam3_14-41040687-41040803_HOXA10_ANCORA
ACAATTAGGGGCTACAGCTTAGGACAACAGGAGGTCAATCAGACATAGCTAATTATGATGCATCTTACTTATGGCCTGTCCAGCAGGAAGAAT
TAGCTGTTTTTGAAATTTATGCC

>Canis-familiaris_DogHorse_canFam3_14-41043381-41043436_HOXA10_ANCORA
AAACTGTGACTTAAGTGGTAGACTATAATTTGTTTTGTATGTGGATGCCAGATTT

>Canis-familiaris_DogHorse_canFam3_14-41051588-41051638_HOXA10_ANCORA
ATATAAAAAGTCTTACCTATTTGATCCCTTAAATGTTACCAAATGGAACC

>Canis-familiaris_DogHorse_canFam3_14-41060857-41060913_HOXA10_ANCORA
TAAAATCACTTTTAATCAGCAAGAAAGGCAGTTGTTTTTAAAACAGTAACATGCTG

>Canis-familiaris_DogHorse_canFam3_14-41063037-41063090_HOXA10_ANCORA
TCAAAGGTTAAAATATGTACATATTGTTCTTCAGCATGAAATCTAAGCAAAGA

>Canis-familiaris_DogHorse_canFam3_14-41070152-41070218_HOXA10_ANCORA
GTGAGAAAATTAAGTAATGTTTCAACTTGAGTCCCAAATCAACAGTCTCAGCAATATGAGAGTATC

>Canis-familiaris_DogHorse_canFam3_14-41084334-41084397_HOXA10_ANCORA
TGCACAATCCCCCTCATAAATATTGGTTTTCACTGCTTACAGTTACAATGTTGGCATGGTTTT

>Canis-familiaris_DogHorse_canFam3_14-41090289-41090419_HOXA10_ANCORA
TTTCAAAAAAAAAAAAAAATCCTACAATTGCACATAACCAGCCTTTGAATGGATCAACTTTTCCTTAGTTTATAGACACGTCATTTGAAACTT
TTCCCAAGCCATAATTTCCATTACAGGGCAGTCAGTG

>Canis-familiaris_DogHorse_canFam3_14-41094633-41094702_HOXA10_ANCORA
CTCCAGAATTGGTTATTAGTGGGCAATAATGAAGCAAATTGCACATGCATATTACTTCTGAGTTATGAA

>Canis-familiaris_DogHorse_canFam3_14-41094714-41094774_HOXA10_ANCORA
CAGTAACCTTTGAAGCTGTCAGGATCTTTTCAGGCAAGCAATTAAAGTGAAAAATGAGAG

>Canis-familiaris_DogHorse_canFam3_14-41100488-41100555_HOXA10_ANCORA
CATCACCTGGCAGGGTGTTTTCTGCTTATTATGCTAATTAGATTTTACTTGAAAGATGTAATTATTG

>Canis-familiaris_DogHorse_canFam3_14-41101060-41101115_HOXA10_ANCORA
AGAGGACATTTTAAGTTCTTCTAACACCTTGGTAATCAGAGCCCAGGGAAATATT

>Canis-familiaris_DogHorse_canFam3_14-41101581-41101644_HOXA10_ANCORA
AAGCATTTTTCATTCAGAGTAATGATCTCCAGGAGTAAAAATAATATAATTATAACTGAACTA

>Canis-familiaris_DogHorse_canFam3_14-41101677-41101789_HOXA10_ANCORA
GGTATGTATTTCATTCACTGTGTCAAAAAATCTTCATTCTGGACCATCAACATAGCACTAAATTTTGATCTGTCATTGAAAACAGGTCGCTTC
TATTTCTGGCTTACAGCAT

>Canis-familiaris_DogHorse_canFam3_14-41103398-41103456_HOXA10_ANCORA
GTGGCAGACTTAGGCCAGAAAACAAACCCACGGCTGCTTGTTAAATCAACTCAAGCAC

>Canis-familiaris_DogHorse_canFam3_14-41103464-41103539_HOXA10_ANCORA
ACAGCTGTAAAAATACAGTGGTTTAAAATCCAAGCTTCTAATAGCCACCCAAATAGATGCTGTACTAGAATTACT

>Canis-familiaris_DogHorse_canFam3_14-41116132-41116186_HOXA10_ANCORA
GCCAGTGTGTCTTTTTCCATTACCCATGTTTAAAAACAGACTCTTTAATTTATG

>Canis-familiaris_DogHorse_canFam3_14-41130652-41130705_HOXA10_ANCORA
GAAACATTAATTAACTGGAGCTTTTTTGCATTTAATTTTCTCTGGCATTTTGG

>Canis-familiaris_DogHorse_canFam3_14-41130802-41130871_HOXA10_ANCORA
TGCTGAGAGTCCAGATAATGCAAATCTAAAGTATTATTAAAGGGTATTCTTTACACATGAAAGAAAAAC

>Canis-familiaris_DogHorse_canFam3_14-41132674-41132724_HOXA10_ANCORA
CAGCACAGAATTCCATGACCACATCTACTTCCCAAGCCTCAGCTCTCCAG

>Canis-familiaris_DogHorse_canFam3_14-41142333-41142434_HOXA10_ANCORA
AATGTTTAATTTGCTCTGATGTTTCTGCTTTCTGTCATTTTTGTATAGTTAACAGGATCATAATTTAAGTAAGTCACACATTGTAAATAACCT
GGCTATAT

>Canis-familiaris_DogHorse_canFam3_14-41142468-41142549_HOXA10_ANCORA
GTTACAAAGAATCCTATAAACCATTTAACCATGAGTGGTTGTAAACTGCTCGTATTAAAAATTGGGTGCTCCCCCTCCTAA

>Canis-familiaris_DogHorse_canFam3_14-41145876-41145942_HOXA10_ANCORA
GGTAGACACAGTCTGCCTTTCTTCCTGCTGTCTGGTTAATGTAGCAACTGGCTGCTCAGGAAGGAA

>Canis-familiaris_DogHorse_canFam3_14-41168923-41169013_HOXA10_ANCORA
CCGCAGTATTAAAACTGCTCTAATTGCATTCTTCAAGCCAGCTGAAGGCTGACAGCTTCCGCTCCCGGCTCATTATCAGAGCACTTCAAG

>Canis-familiaris_DogHorse_canFam3_14-41169014-41169239_HOXA10_ANCORA
GGCTCCATTATGAGCAGAGCACCTGGTCCAAATTTATTTCAACGGCCCAGGGCAAAATGGGAAGGTGCTTAATGTTCAACCACAATGGTGACA
TTTTTATGAGATATCCAAGGTTCAGAAACAAATAAATGAAAGGTTTGAAAGGAGCACGAACAGAAACTGTGACAATAAAAATTCAACATTAAT
ATATCAATTTTAAACCATATACCACCCATACACTTGGGG

>Canis-familiaris_DogHorse_canFam3_14-41178502-41178598_HOXA10_ANCORA
CATTCCTATATAAAACAATCTAAAATGTCAGCACTCTGTCTTTATGACATCATGCCATCCCAATAATTCTGTTTTACAAGTAAATTCCTATTA
TAC

>Canis-familiaris_DogHorse_canFam3_14-41178992-41179100_HOXA10_ANCORA
TGATGCATGAAAGAAAGAACTTTACTCACTTTGTTAACATGTTTTTGTTGTAATTAGCTTGGGTCAGTGAAGGAGACATCAAATTAAAGTTTT
ATGTCATAATAGGAG

>Canis-familiaris_DogHorse_canFam3_14-41187259-41187385_HOXA10_ANCORA
AGTATTTTGAACCAGCAAATTCAAGCCTAATTACTAAAAACAGGTTGTGGAAAATATTACTAAGTGGCTTAAATACCATCTGCACCACATCAG
GCACACATTCCTAATGAAAAGGGCACATGTGTG

>Canis-familiaris_DogHorse_canFam3_14-41222450-41222501_HOXA10_ANCORA
GTTTTTAATGAAATGTAGAGCATTATTAATTGGTTTCCTAGCAACAGCCAG

>Canis-familiaris_DogHorse_canFam3_14-41222510-41222584_HOXA10_ANCORA
ACTTAACATGTCTGCTCAATCATATGCAAATATGAGTGTTATCCCTTAAATGTTGATTATAGTTGGTCTTTTCA

>Canis-familiaris_DogHorse_canFam3_14-41229441-41229499_HOXA10_ANCORA
ACACATAAGGAAGCAATTTCCAGCCATAATTTTAATTAGGAGCACAGAATGATCTTCT

>Canis-familiaris_DogHorse_canFam3_14-41234099-41234175_HOXA10_ANCORA
GGAGAAGCTTCAGCATTGCTGTGACTATTTCAACAGTAATTATTAAGTTGCTATGCCTTCCTTCCCTAATAAAAAA

>Canis-familiaris_DogHorse_canFam3_14-41238375-41238429_HOXA10_ANCORA
AAGATTAAAGATACTGCTGATTTGTAAAAGGTTGAAATATTAGAGGAAATAAAA

>Canis-familiaris_DogHorse_canFam3_14-41249558-41249611_HOXA10_ANCORA
AATTTGGGAACCTCTTACAGCTCTCTGGCCTGCAGTAAGTAATCCTTTCTTCC

>Canis-familiaris_DogHorse_canFam3_14-41339016-41339082_HOXA10_ANCORA
GCTTTGAAGAGACAGAAATTCCAAACAAATACAGATGGTCAGACCATAAACTTAATTAAAATTTCT

>Canis-familiaris_DogHorse_canFam3_14-41343319-41343369_HOXA10_ANCORA
AGAGCTAGATGTTAGCCTAGAGGTTTGACAAGCAGATGACTTCCAGACAG

>Canis-familiaris_DogHorse_canFam3_14-41370769-41370822_HOXA10_ANCORA
TTCTGTCTTTGATTTTAGGTAAGAATTGAATGCAGAAATCTGCAATTTCTCCC

>Canis-familiaris_DogHorse_canFam3_14-41377936-41377990_HOXA10_ANCORA
ATATTTCCTAAAAGATAATGGGTTTCAAAACCAAACATAAATCTTCAGTGACAT

>Canis-familiaris_DogHorse_canFam3_14-41410133-41410247_HOXA10_ANCORA

CTCAAGAAATAAAAATTTAATAGACATATATCTGAAAGTTTCTAGCACTTCAGGGAGATTTTGAGATTTATTCTAGCATACCATACAATGAGT
TTTATTGGGAACTGCAAAAAT

>Canis-familiaris_DogHorse_canFam3_14-41413155-41413218_HOXA10_ANCORA
TAATCTGGAAGCGATCAGTGGAGGAAAAAGATAACACAGCTGTGATTTTCAAGGAGACAGGTC

>Canis-familiaris_DogHorse_canFam3_14-41424787-41424837_HOXA10_ANCORA
ATTTGGGGATAAATGAGTTACTTTATATTCCATTTCCTTTTTCAAAAAAA

>Canis-familiaris_DogHorse_canFam3_14-41424859-41424921_HOXA10_ANCORA
CCTTATTACATTTAGCTTCTGTTCTCATAATCCATCAAAATTACTAGCTGAAAGATCAAAAA

>Canis-familiaris_DogHorse_canFam3_14-41426489-41426568_HOXA10_ANCORA
GCACTCAGAAAACAAACCCAGATTCAAATTTTAAACATTCATAAAAATCAGAATGGTGATATTTTTGACCTGCTGCCAA

>Canis-familiaris_DogHorse_canFam3_14-41427077-41427138_HOXA10_ANCORA
AGAGCAGATGCTTAAGTACTTTTTAAACCGATGGCTTTGTGGAGCTGTTTTCGCTTAAGGA

>Canis-familiaris_DogHorse_canFam3_14-41435517-41435569_HOXA10_ANCORA
CTTGCTGACAGCATCCATCTTTCCATTCACAAGTCACATGAAATATATGGCC

>Canis-familiaris_DogHorse_canFam3_14-41438274-41438359_HOXA10_ANCORA
TGAGATTTCCCTTTCCTATTCCTTTCTCCTCATCTATTAATGAGCATGAACTACATCCTGGCCTTTAATCATTGATATCATTTCA

>Canis-familiaris_DogHorse_canFam3_14-41438473-41438532_HOXA10_ANCORA
GAGCTAAATTTCCTATGCACCTTCAAGCATTAAATTGAAAATTAAGCTAAATGTACCAT

>Canis-familiaris_DogHorse_canFam3_14-41438576-41438638_HOXA10_ANCORA
GGTATCCAAGAAAGTTATGTGGTCCATATGCTATGGAGGAATTTATAGTCCACAGTAATAAA

>Canis-familiaris_DogHorse_canFam3_14-41441910-41441974_HOXA10_ANCORA
ACAACATGCTTTTCCTTCAAGCAATTTATGTTAATAGAAGAGTGTCCTAAAGAAAAATTAAACA

>Canis-familiaris_DogHorse_canFam3_14-41442422-41442490_HOXA10_ANCORA
AAGAGAGCATGACAGCTGTGGCAGGTACAAAACAGAGGTGCCTTGTTCCACTGAGTGACACTAATTAT

>Canis-familiaris_DogHorse_canFam3_14-41442571-41442631_HOXA10_ANCORA
GTCTATAAATAAATAGAAAATAACGGCACTTTTCTAATTGAATGCAGATAGGTAAATAAA

>Canis-familiaris_DogHorse_canFam3_14-41452812-41452862_HOXA10_ANCORA
AGTTAAATTAACAGTATTTCATAGGATTTATTAGCCATAAAAAGTCCCCC

>Canis-familiaris_DogHorse_canFam3_14-41465716-41465806_HOXA10_ANCORA
ATTATTTAATGAACAATTCCTCTTCTGGTCTGTATTAACAATGGTGCTTGGAACTCGGCATCGATAACACACTGTATGCCCTGTTGTGCT

>Canis-familiaris_DogHorse_canFam3_14-41465807-41465897_HOXA10_ANCORA
CATGTTGAATGATTTGTGTGTCTTTGAGAAAGTCCAGCTTCTCCTCTTAATCTGGTATCCTGGAGGCTCTGTAATTTACTCCCACAGTTC

>Canis-familiaris_DogHorse_canFam3_14-41472098-41472155_HOXA10_ANCORA
AACTCTGGAATTTGGGCTTGCTGAATGCATGCCATTCCCTTGCCACGTAGCACAAAT

>Canis-familiaris_DogHorse_canFam3_14-41472220-41472274_HOXA10_ANCORA
GCTCAATTAAACTGGTTATTCCACAAATAACCTAGAAACAGCAGGATTGCCAAA

>Canis-familiaris_DogHorse_canFam3_14-41472380-41472438_HOXA10_ANCORA
TTTTGGCAGCAGACTGTAGCAAACATGATTCTACACTTATTTTCTTTTAATCAGAAGA

>Canis-familiaris_DogHorse_canFam3_14-41476402-41476460_HOXA10_ANCORA
CTGAAAATAGATGGTTTTTCTAAACAGCACAATGTCCCAGTGTACTGTTTGTACTGCA

>Canis-familiaris_DogHorse_canFam3_14-41476666-41476718_HOXA10_ANCORA
CTGATCAATTAGGTTTAAAAGCTACTGAAATTCTCAAGAATCATTTAAATAA

>Canis-familiaris_DogHorse_canFam3_14-41476943-41477000_HOXA10_ANCORA
CTTACCATTGATCTTAAGGAAATGACTAAAATTTTTGGAGTGTGCTGACACAGCACT

>Canis-familiaris_DogHorse_canFam3_14-41484627-41484716_HOXA10_ANCORA
TGAGGATTGTTTCCAAGCTGACTCACACCACTAATGTCAGGGAGCCGTGTCACACGGCGCTGGGCTCTGCAAGCTGTTGCTTGCGCGGT

>Canis-familiaris_DogHorse_canFam3_14-41485006-41485057_HOXA10_ANCORA
GTAAGTTCCTAAAAATACGGCTCGGCCACTTCTGGGGCTTTTGCATTTAGC

>Canis-familiaris_DogHorse_canFam3_14-41485209-41485263_HOXA10_ANCORA
GGAGGGTGATCTGTGTTTGTAAACACTTTGGCGGACCAGAGGGAAGTCCGGCCC

>Canis-familiaris_DogHorse_canFam3_14-41485605-41485665_HOXA10_ANCORA
TGCGGCGGAACCCCCGGAGAAAGTTTGCAAACTTCCAGCGGGCGCGGCGAGGACGCCGGG

>Canis-familiaris_DogHorse_canFam3_14-41487618-41487775_HOXA10_ANCORA
GCACTGGAGAGTGTGATTAATGATCAGTGATTTAGTGATCAGTGATTAGTGGTTTTATTCAAACTGTTACCTCTGACCAGAAGCACACATCAG
AGGATTAACAGGCTCTTTAGATAAATGCTCATTAGTGGAAGGGGAAAAGCAAAGATAATAGGCA

>Canis-familiaris_DogHorse_canFam3_14-41487900-41487986_HOXA10_ANCORA
ATTTAAAACTTGCTTTTATCTTGTAAATCAAAGTTAGATGTAAAAGTTTGTAGTGCCATTATAACCTAAATTTTCCCATATCCACT

>Canis-familiaris_DogHorse_canFam3_14-41488046-41488112_HOXA10_ANCORA
AGAGCAGCTGAACCCTGACAAGTTCTTAACTCCCAGGGTTTGAAGGTGTCCCCCCTTCTAGAATGG

>Canis-familiaris_DogHorse_canFam3_14-41488223-41488304_HOXA10_ANCORA
CAACAAAGTTGATTCTGTGTAGGGTTGGAGGCTAGACAGTTCTACAAGTTTTTAGTCACATTTTCCATGTCAGTTAAATCT

>Canis-familiaris_DogHorse_canFam3_14-41489933-41489991_HOXA10_ANCORA
GCTTCTTTTGCTTCTTACAAATTGGATTTCTACAGCAATTTAAGATCTTTTTTTAGAG

>Canis-familiaris_DogHorse_canFam3_14-41490016-41490077_HOXA10_ANCORA
CAATGATCTCAGTCAATTCATTATCTTGTCCAGTTTGACTACCAGCTGTATTATGGGAATC

>Canis-familiaris_DogHorse_canFam3_14-41507191-41507242_HOXA10_ANCORA
GTGACAGAACTTACAAGAAATTCATTCAGAAAAACATCCAGTGTAGGAGTC

>Canis-familiaris_DogHorse_canFam3_14-41520099-41520157_HOXA10_ANCORA
AGCTGTACACACACTGGAGCATTTGGGGCTGACTTGGCAGGATGATGTGGCTGCCGGC

>Canis-familiaris_DogHorse_canFam3_14-41529685-41529745_HOXA10_ANCORA
TTTTTTTTCCCAAAGAATCCATAAATATTTACATAAGTGCAAGTTACAAATCTGTGGCTT

>Canis-familiaris_DogHorse_canFam3_14-41531898-41531960_HOXA10_ANCORA
TAATGGGCTGGGTCGATGTCATGCAGATCTGATGACAGGACACCCACACCCTGTCACCCTCC

>Canis-familiaris_DogHorse_canFam3_14-41538170-41538230_HOXA10_ANCORA
GGTGTGGCTTTGAAGTGGAGAAAGTGTGACTGTCAGGTTTTAAAAAGGAAGCAGAGAGAC

>Canis-familiaris_DogHorse_canFam3_14-41541748-41541800_HOXA10_ANCORA
TATTCTTGCTTGTTATTTTTGTCACCATAAAATAAACCATATTTACATGATT

>Canis-familiaris_DogHorse_canFam3_14-41545615-41545712_HOXA10_ANCORA
GTGTTTTGTAGGCAGTGAAGTTAAGGCTTGCCCAGAAAAATAACATGTTTTCCTTGCCTGGTGCTTGAGCTGTTTGCCTGGTGACTGGCTGAT
TGGC

>Canis-familiaris_DogHorse_canFam3_14-41572944-41572995_HOXA10_ANCORA
TTGCACATTTCCTGGCTTACTTGGAACTGGTACCATGTGGGATCAAAGACA

>Canis-familiaris_DogHorse_canFam3_14-41573032-41573116_HOXA10_ANCORA
AAAAGTTAAGCTCCATTTTCTGGTTCCTGTTGTAACTGGAAAGAATAGCTCATTCACAAGAAGAAAATGAATTAAAAATTAGGA

>Danio-rerio_ZebrafishStickeback_danRer10_16-21085042-21085201_HOXA13B_ANCORA
GAAATGTAGTTAGTGGTATGTTTCTGATCTACCCACCAAAGAGCCACGGAGAGGCCATAATGCTAGAGGTCACATGATCACAGGACAAACCCA
TAAAAAGTCCATTAAGCATGGAGGCCTAAGTGCTGTGTTGTTATCTAGTTGGCAGGTCTTACAAAC

>Danio-rerio_ZebrafishStickeback_danRer10_16-21089168-21089199_HOXA13B_ANCORA
AAAACACGATCATGCTAAATTCGTGGCATCA

>Danio-rerio_ZebrafishStickeback_danRer10_16-21089962-21090020_HOXA13B_ANCORA
GTTTAGACGTTTATCAACCTCTTTGCATGTCACGTGCCTGCTCTCGTCCAATAACAGT

>Danio-rerio_ZebrafishStickeback_danRer10_16-21090029-21090067_HOXA13B_ANCORA
ACGCACGCGGGGCGCATGGGTATCTGTTACTCCACTGA

>Danio-rerio_ZebrafishStickeback_danRer10_16-21092807-21092840_HOXA13B_ANCORA
TCCAAACGTTTACTGAAATCAAGATTCAAATTG

>Danio-rerio_ZebrafishStickeback_danRer10_16-21098875-21098934_HOXA11B_ANCORA
GGTACATGTCAATTTCTGCCCTTGGTCACATGACTGGCGCTCTCTGGAATGGATGGAGA

>Danio-rerio_ZebrafishStickeback_danRer10_16-21099729-21099868_HOXA11B_ANCORA
TGTTGTTAAATGTTTTATATGCTGTTTTATAAGCATATAAGGTTTATAGAGGGCTCTGTATATTTACCCTAACAAAACTTTGTTATAAACGCG
TGATCACGTGCTCGAAATTGAAATTGGCCGTGAATGATAGGCCATT

>Danio-rerio_ZebrafishStickeback_danRer10_16-21102034-21102140_HOXA10B_ANCORA
TGCGCATCCTTTGAGGCTCGATGGAATTTCGCCCTAATTACGGCACATCCCCCCGTTGCTGCAGCAACTCAGTCATAAAACCTGTCTTAGTCT
GGAGCATTTGTAG

>Danio-rerio_ZebrafishStickeback_danRer10_16-21102148-21102276_HOXA10B_ANCORA
TGAGGTGCCATAAACCGTCTGAGAACCAAGGTTATTAACTGTGACTAAGAGCTAGAAAACAACGGCTGGAACCATTGAAAGCTTGTGTTCACG
GACTTTTCCGCCATCATTTGTCTCAGTGTCCCATC

>Danio-rerio_ZebrafishStickeback_danRer10_16-21102331-21102380_HOXA10B_ANCORA
TCCACTGGATGGCGCACTTTAGATCAATGTCATTGCCGGTATCTAAAAT

>Danio-rerio_ZebrafishStickeback_danRer10_16-21104270-21104340_HOXA10B_ANCORA
GCTCGTTGCGATTTAATTGGCTGTTAGCTCACACTGAGTTGTATAGTTATCAGTAATTATATTCAGAATG

>Danio-rerio_ZebrafishStickeback_danRer10_19-20153643-20153693_HOXA13A_ANCORA
GTTTAACTATGACCAACTTGAACTTGACCATGATACGCATAGAATGTAAT

>Danio-rerio_ZebrafishStickeback_danRer10_19-20155688-20155731_HOXA13A_ANCORA
AAGCTTTTAGACTGGCCATCAAAGTCAGAATAGGCCAACAGGC

>Danio-rerio_ZebrafishStickeback_danRer10_19-20155820-20155850_HOXA13A_ANCORA
GGTGTTCTCCGTCTCTGCAACTTGTAAACA

>Danio-rerio_ZebrafishStickeback_danRer10_19-20160740-20160786_HOXA11A_ANCORA
GGTCGTCTAGACAATGAAAGCCAATGCCACAGTCACAATATCTCAA

>Danio-rerio_ZebrafishStickeback_danRer10_19-20162976-20163011_HOXA11A_ANCORA
CCCAAATTACTGCTGAAAACTTTCCCGCACGCGGC

>Danio-rerio_ZebrafishStickeback_danRer10_19-20163014-20163046_HOXA11A_ANCORA
GAATTTGTTATAAGCTGACGAAGAGGCAGAGC

>Danio-rerio_ZebrafishStickeback_danRer10_19-20164141-20164255_HOXA11A_ANCORA
GACGATCTGTCAATTTCTTCACATGATCACGTGACCCTGGCCTCAGTTGGAGCGGATAGAGATGGATTTCCACGTCATCTTACGTCTCAAAAT
TTCTGCCTCGCGGATATCCCT

>Danio-rerio_ZebrafishStickeback_danRer10_19-20165111-20165162_HOXA11A_ANCORA
ACACTGTGAAATTCAAGTGCTTGGAATTGAAATTAGCCATGAAAAGACTTA

>Danio-rerio_ZebrafishStickeback_danRer10_19-20166933-20166972_HOXA11A_ANCORA
TAGCGTTCATATATGGCGGCCAAAGAGCAATAAAGTCAG

>Danio-rerio_ZebrafishStickeback_danRer10_19-20167011-20167188_HOXA11A_ANCORA
GGCCTGGCTGGCGCATCCTTTGAGGTGCTGAGGAATATCCGCCTAATTACGGGGACATCCTCCTTGTTGCTTCAGCAACACGGCCATAAAAGC
TGTCTGTGTCTGGAGCATTTGGGCAATTGCAGCGTGGTGCCATAAACTGTCTGAGAACCAAGGTTATAGCACGATTCCAAAGGG

>Danio-rerio_ZebrafishStickeback_danRer10_19-20167330-20167371_HOXA11A_ANCORA
CATTGGGAGGCACTCGTGAGATCAATGTCATGGCCGTGGAT

>Danio-rerio_ZebrafishStickeback_danRer10_19-20209265-20209305_HOXA1A_ANCORA
CATTGGAATAAACCACAAACTGCCATCAGGTAATGGTAAT

>Danio-rerio_ZebrafishStickeback_danRer10_19-20212955-20212986_HOXA1A_ANCORA
CGTAACGTTGATGGACAAGAGGTCGGCATTT

>Danio-rerio_ZebrafishStickeback_danRer10_19-20213053-20213105_HOXA1A_ANCORA
AACTGATCACTGGCTGACCCAGCCCACGTGACTGTCACTGGTCATTCATATC

>Danio-rerio_ZebrafishStickeback_danRer10_19-20214936-20214969_HOXA1A_ANCORA
AAGTTGTTCTCCGGTGATGCTGAGTTTTATCAG

>Danio-rerio_ZebrafishStickeback_danRer10_19-20219115-20219207_HOXA1A_ANCORA
AAGGACAGAAAGAGAGAGGTCCATCCATCACACTGTCTTAACCTCACTGTGACCCCAGCCTGGCAGCCAATGAGGCACCTCCCGTCTGTGCA

>Danio-rerio_ZebrafishStickeback_danRer10_19-20237808-20237837_HOXA1A_ANCORA
ATCACTGCCGGACGTTGGCAGGGATCATT

>Danio-rerio_ZebrafishStickeback_danRer10_19-20237853-20237933_HOXA1A_ANCORA
TGTCACCAATGTGTCTGTGTGGCTGCTGATGTGATCTTTAGTCCCCTCATGATCTATAGACTGGCATTTTGGTCGCTCAG

>Danio-rerio_ZebrafishStickeback_danRer10_19-20237978-20238020_HOXA1A_ANCORA
CCTGACAGGAACTTAATGCAGTCACGGCAATAGTGAGACAGG

>Danio-rerio_ZebrafishStickeback_danRer10_19-20241840-20241871_HOXA1A_ANCORA

TTTACATAAACAGCCGCTTAGTGGGATTAGC

>Danio-rerio_ZebrafishStickeback_danRer10_19-20266160-20266213_HOXA1A_ANCORA
CTCTGATTCTGACATGGCTCATACATCACAACTCTTGAGTGTGTGTGATTACA

>Danio-rerio_ZebrafishStickeback_danRer10_19-20272275-20272343_HOXA1A_ANCORA
GGATGCATTAGCTATGCAGGAAACATCTTAATTAATCTTGATGATGACAGCGTGACTGTCCTTCCGGA

>Danio-rerio_ZebrafishStickeback_danRer10_19-20272347-20272390_HOXA1A_ANCORA
TCCCCATCGATTCATAAATAACCATTGAGATTTATAATGAATG

>Danio-rerio_ZebrafishStickeback_danRer10_19-20281514-20281703_HOXA1A_ANCORA
CAAGATTATTATTAGGACATGGCTTTACAAAGTAAGCCAATTTACACAAGCAATCCTGCTTTGAATTAGCAGGTAATGACTCAATTAGCTTTC
ATTTTTGCACAGAGGAGATTTGGCTGCATAAATTAGCCTCCATTAATCGGGAAAGTGTCAGTAGCAAAGGACAGCTCACAAATACTGCAGCCA
ATC

>Danio-rerio_ZebrafishStickeback_danRer10_19-20281726-20281816_HOXA1A_ANCORA
ATATAGACGTCCGGCCAGACTTTGTGTTTGAGCTGAACGGACCGGTGTCACTCAGAGTGTGACACGGGAACAGGAGGATAGCCCAGGGGA

>Danio-rerio_ZebrafishStickeback_danRer10_19-20281819-20281942_HOXA1A_ANCORA
CTGGCCGCCTATGCCCTGAAAAGGTCAACTTGACACAGGCAGCGTCCCATCGACCTGTCCCCTGTTCGGCCAGTGGAAAATCAGATAAACTTA
AGACCACTCATCGATCCTCAGATACACAGC

>Danio-rerio_ZebrafishStickeback_danRer10_19-20283367-20283412_HOXA1A_ANCORA
CAGACATATTCCGTCCGTAAGGTTAAGCGTTCCTAATATTACGGA

>Danio-rerio_ZebrafishStickeback_danRer10_19-20283413-20283446_HOXA1A_ANCORA
CATTTGTTTATTGGCATGTCTCAGTTTAAGGAC

>Danio-rerio_ZebrafishStickeback_danRer10_19-20283547-20283576_HOXA1A_ANCORA
CGGAGGCGCGAACTGAACTCTTCACCTGG

>Gasterosteus-aculeatus_ZebrafishStickeback_BROADS1_X-9833780-9833811_hoxa1a_ANCORA
GGACACTCGCTGACCAGATACGGTGTGTGGA

>Gasterosteus-aculeatus_ZebrafishStickeback_BROADS1_X-9835453-9835503_hoxa1a_ANCORA
GTAATCAAACGGGCTCCAGCCGTGATGTATGAGCCGTGTCACAATTACTG

>Gasterosteus-aculeatus_ZebrafishStickeback_BROADS1_X-9845281-9845311_hoxa1a_ANCORA
ATAATCCTGCTAACAGGGTGTTTATCTGGA

>Gasterosteus-aculeatus_ZebrafishStickeback_BROADS1_X-9846378-9846420_hoxa1a_ANCORA
CTGTCGGAATATTGCGCTGGCTGGCATTAACTTCCTGTTGGG

>Gasterosteus-aculeatus_ZebrafishStickeback_BROADS1_X-9846501-9846575_hoxa1a_ANCORA
TGAGCCAAATGCCAGTCTATAGATCGCAGAGGGGACTAAAGATCACATCATCGTCCACCCGGACACGGGTGACA

>Gasterosteus-aculeatus_ZebrafishStickeback_BROADS1_X-9846609-9846636_hoxa1a_ANCORA
ATGATCCCAGCCAGGACGAGCCGTTAT

>Gasterosteus-aculeatus_ZebrafishStickeback_BROADS1_X-9849967-9850009_hoxa1a_ANCORA
CAATGCAACTAATTACTTATTTAATAAAACACACACATATAT

>Gasterosteus-aculeatus_ZebrafishStickeback_BROADS1_X-9849971-9850004_hoxa1a_ANCORA
GCAACTAATTACTTATTTAATAAAACACACACA

>Gasterosteus-aculeatus_ZebrafishStickeback_BROADS1_X-9850522-9850614_hoxa1a_ANCORA
TTAGAGGGTGAGGGCGCCTCATTGGCTGCCATGGCGAGGTCAAGCTGAGGTTAGGACATGTGTGATGGATGGACCTCTGTCTCTAGACCACT

>Gasterosteus-aculeatus_ZebrafishStickeback_BROADS1_X-9853365-9853401_hoxa1a_ANCORA
TCCTCATCCTATCATTCAAAATATGTTTAATCATAT

>Gasterosteus-aculeatus_ZebrafishStickeback_BROADS1_X-9854049-9854090_hoxa1a_ANCORA
AATACTAACCAAACGTATTCAACGTTTAACGACAATATCAT

>Gasterosteus-aculeatus_ZebrafishStickeback_BROADS1_X-9854719-9854751_hoxa1a_ANCORA
TGATAAAACTCAGCTCCCCGCTAATACAACAT

>Gasterosteus-aculeatus_ZebrafishStickeback_BROADS1_X-9856723-9856775_hoxa1a_ANCORA
ATATCAATGAATGGCTGCTGTCACGTGTGTCTGGGTCAGCCAGTGAGGTACT

>Gasterosteus-aculeatus_ZebrafishStickeback_BROADS1_X-9856825-9856855_hoxa1a_ANCORA
AATTCCATGCGCTCGTCGATCAAGGTGACG

>Gasterosteus-aculeatus_ZebrafishStickeback_BROADS1_X-9912070-9912247_hoxa11a_ANCORA
TCCTTGGAAACAGTTAATAACCTTGGGTCTCAGACGGTTTATTGCACTGCACTCCAGTTGTACAAATGCCCCAGACTCAGACAGCTTTTATGG
CACCGTTGCTGCGGCAACGGGGAGGATGTCCCGTAATTAGGGAAGGAATTCCAGCGCGCAGTAAAGGATGCGCACGGCAAGGCC

>Gasterosteus-aculeatus_ZebrafishStickeback_BROADS1_X-9912292-9912331_hoxa11a_ANCORA
CGCGTTTATGGCCCTTTTGACTGTCATATATGAACGCCA

>Gasterosteus-aculeatus_ZebrafishStickeback_BROADS1_X-9914817-9914866_hoxa11a_ANCORA
CGTCTCTTTCACGGCCAATTTCAATTCCAAACACGTGATCCTGCAGTTT

>Gasterosteus-aculeatus_ZebrafishStickeback_BROADS1_X-9915787-9915900_hoxa11a_ANCORA
AGCAGGTTCGCTAAGCAGACATTTGGAGACGTAGGCTGACGTGGAGAGCCATCTCCATCCACTCTACGGAGGAGGAGGTCACGTGACCAGACG
GCAGAAATTGACAGATTTTC

>Gasterosteus-aculeatus_ZebrafishStickeback_BROADS1_X-9916657-9916685_hoxa11a_ANCORA
ACCTACCAATAATGCATTTGTTCATTTA

>Gasterosteus-aculeatus_ZebrafishStickeback_BROADS1_X-9916751-9916777_hoxa11a_ANCORA
CACCTCTCAGTGAAGACACGGTTCAC

>Gasterosteus-aculeatus_ZebrafishStickeback_BROADS1_X-9917834-9917864_hoxa11a_ANCORA
CTCCGCCTCCCCGTCCACCCAGACAAATTC

>Gasterosteus-aculeatus_ZebrafishStickeback_BROADS1_X-9917868-9917900_hoxa11a_ANCORA
ACTTGTGCTGAAGCTTTTAAGCTGTAATTGAG

>Gasterosteus-aculeatus_ZebrafishStickeback_BROADS1_X-9920362-9920399_hoxa11a_ANCORA
ATAAAAATGCAATTTCATATTTAATAGCTGCAGTGGC

>Gasterosteus-aculeatus_ZebrafishStickeback_BROADS1_X-9925307-9925355_hoxa13a_ANCORA
GAAATCAAATGAAATATTTCAGTGTTTATGGTTTAAAATGCCATAACA

>Gasterosteus-aculeatus_ZebrafishStickeback_BROADS1_X-9926467-9926499_hoxa13a_ANCORA
CGCCGCCATTGGCTCAGATGTTATCACGTGAC

>Gasterosteus-aculeatus_ZebrafishStickeback_BROADS1_X-9927287-9927316_hoxa13a_ANCORA
GTTGACAGCATGCAAAGAGAGACAACAGC

>Gasterosteus-aculeatus_ZebrafishStickeback_BROADS1_X-9927408-9927451_hoxa13a_ANCORA
CCCGTCGGGCTATTCAGCCTTTAAAGGACCCGTCTGAGGGCTT

>Gasterosteus-aculeatus_ZebrafishStickeback_BROADS1_X-9928408-9928454_hoxa13a_ANCORA
TACACAGTGTAGGTCAGCATTGTGTTTTTTTGTTAATAACAGAAAA

>Gasterosteus-aculeatus_ZebrafishStickeback_BROADS1_XX-9707342-9707370_hoxa13b_ANCORA
GTAAATAAATACAAATTGAAATGTCTAA

>Gasterosteus-aculeatus_ZebrafishStickeback_BROADS1_XX-9708001-9708157_hoxa13b_ANCORA
ACCAGGAGGGCGGCATGTTTCTGATCTAGTTTGCCAAGAGCCACTGAGGGGCCCCGGCGCTCCAGGTCATGCGATCACAAGACAAACCCATAA
AAAGTCCATTAAGCACAGTAGGCTGAGAGCTGTGTTGCCATCTATTTGATAGGTACCGCAAAC

>Gasterosteus-aculeatus_ZebrafishStickeback_BROADS1_XX-9709696-9709722_hoxa13b_ANCORA
AAACAATTTCATGGAATTTGTCATCA

>Gasterosteus-aculeatus_ZebrafishStickeback_BROADS1_XX-9710425-9710482_hoxa13b_ANCORA
TCTAGATGTCTACCATCTCCTTTGCATATCACGTGCTTGGCCCTGGCCAATAACCTT

>Gasterosteus-aculeatus_ZebrafishStickeback_BROADS1_XX-9710492-9710528_hoxa13b_ANCORA
TGGACTCAGGCGTGTTGGTATCGTTACTCTGCCCGA

>Gasterosteus-aculeatus_ZebrafishStickeback_BROADS1_XX-9716671-9716730_hoxa11b_ANCORA
CTATCTGTCATTTTGTGCCAGTGGTCACATGGCTTTTGGCCCTCAGGAATGGATAGAGA

>Gasterosteus-aculeatus_ZebrafishStickeback_BROADS1_XX-9717507-9717654_hoxa11b_ANCORA
GCTCTTAAAGCTTTTTTTTTATATGCTGTTTTATAAGCACGTAAGGTTTATGGAGGGCCGTGCAGATTTGCCCTCTAACAAAACAATGTTAT
AAACTGCAAGATCACGTGCTTGGGAATGAAATTGGCCGTGAAGACTTTGGCGTT

>Gasterosteus-aculeatus_ZebrafishStickeback_BROADS1_XX-9719986-9720092_hoxa10b_ANCORA
GTGCATCCTGTGAGACTCCCAGGAATTCGGCTTTGATTGCTGCACATCCTCCCAGTTGCTCCAGTAACTTGGCCATAAAAGGCGGATTCGTCT
GGAGCATTTGGAG

>Gasterosteus-aculeatus_ZebrafishStickeback_BROADS1_XX-9720093-9720220_hoxa10b_ANCORA
GCAGTGCAATAAAGCGTCTGAGACCAAGGTTATTAACTGTGACTATAGGGGCTGAATACAACGGCCGGGAGCATTGGAGGCTTGTGTTCACGG
ACCTTTCCGGCGTCATTTCTTTCAGCATCGGACC

>Gasterosteus-aculeatus_ZebrafishStickeback_BROADS1_XX-9720277-9720325_hoxa10b_ANCORA
CCACTGGGTGGCGCTGCTCAGATCAATGTCATTGCCATGAATTGAGAT

>Gasterosteus-aculeatus_ZebrafishStickeback_BROADS1_XX-9722390-9722458_hoxa10b_ANCORA
TTGCCTGGTGTTTTAATTGGCTGCAGGCTACAGAGAGAAGCGTGAGTATCAGTAATTATTCAGCAATG

>Gasterosteus-aculeatus_ZebrafishStickeback_BROADS1_XX-9723796-9723827_hoxa10b_ANCORA
TTAGGCTATATGATTGCGTGGTATGTGTCAC

>Homo-sapiens_Sarcopterygii_GRCh37.p13_7-27130687-27130687_HOXA1_Matsunami13
AAGAGGGGAGAGGAAAGTGTTTTAAGTTTCTAGTAAACATTGTTTAGCAAAAGAATAATAGGAAAGTTGCCCCTTTTCCAAAGAGGGATGGAA
CTATAATTGTTGCCTTTAGTTGAGAGCTCAGATAAACTGCTGGGACTCATTCTAAAGTGACCCATCACTGCATTCATCTCTTCTTGAACTTTC
GGTGAACCTGAGATTAAACAGGGGTGAGCAGCTAAGCAGTTTGTTGTTAGCAGCCTTTTCAACAACCCCTTTCAGACTGAGTAAATATTGATCG
GTTTGAATCTGATTGCCCCAGAGGAAAACACCACCGCATTTGAATAGCCTCCAAAAGTAAACTTTAAAAGGTGAAACCCAGAGCAGACTTCAG
AGCCAAAGCAAGACCT

>Homo-sapiens_Sarcopterygii_GRCh37.p13_7-27223843-27223843_HOXA11_Matsunami13
CAGGCATGCCTTGGCCGGTGGGTATTTCACGGCCAATTTCAGCACTCGCCACGTGATCCCGCCTTTTATAACAAAGTTTTGTTGGGGGAAACC
TAAAGGCCCTTCATAAACCTTATATGCTTATAAAACAGCATATAAAAATTTAACAGCGGTGCTGCGCTAGATTTCCAACTCCCCTTTCATAAA
GCGCAGGGCGCTGCCTTTATACG

>Homo-sapiens_Sarcopterygii_GRCh37.p13_7-27224767-27224767_HOXA11_Matsunami13
TGGGCTACCTTGGGCTCTCCGCAGTAGCCGAGCTTAACATGATTCTCCACTGCAGCTGCCTCTTTGAAGCGGATCCGTGAAGTAGAAATTTGG
AGACGTAAGCTGACGTGGAAATCTATCCCCATCCTTAGCAGGGAGGTGCTGGTCATGTGACCCGATGTTGAAATTGACAAGCTGCTAGCTAGT
CC

>Homo-sapiens_Gnathostomata_hg19_7-27096564-27096754_HOXA1_Matsunami10
ATGATTATTCTTAAGGAGGAAACACAAACAGCAGTGACCATTTTTCACAGACGTGCTCAGCCATCAGGAAAGCTTAATAAATCCCTATTTAGT
ATGCATTTATGCAGTGATTACATGAACTTTGACCTCTGAAGAGCTGTAGCAATTAGCTTCAGTGTCTAGCATCTTCTACTCACCATTCCCCAC
TGGCT

>Homo-sapiens_Gnathostomata_hg19_7-27097212-27097599_HOXA1_Matsunami10
AAGAGGGGAGAGGAAAGTGTTTTAAGTTTCTAGTAAACATTGTTTAGCAAAAGAATAATAGGAAAGTTGCCCCTTTTCCAAAGAGGGATGGAA
CTATAATTGTTGCCTTTAGTTGAGAGCTCAGATAAACTGCTGGGACTCATTCTAAAGTGACCCATCACTGCATTCATCTCTTCTTGAACTTTC
GGTGAACCTGAGATTAAACAGGGGTGAGCAGCTAAGCAGTTTGTTGTTAGCAGCCTTTTCAACAACCCCTTTCAGACTGAGTAAATATTGATCG
GTTTGAATCTGATTGCCCCAGAGGAAAACACCACCGCATTTGAATAGCCTCCAAAAGTAAACTTTAAAAGGTGAAACCCAGAGCAGACTTCAG
AGCCAAAGCAAGACCT

>Homo-sapiens_Gnathostomata_hg19_7-27097824-27098084_HOXA1_Matsunami10
AACTGTGCTCCTTAGAGACCAAAGCAGAGAGACCTTGAAATAGGATGTGTTAAGCGCCTTTGATTTAATCGATTGAGACAGCTAAAAAGATAC
AAATGTTTTCCTTGTTGAAAGGTGTATTAATGTTTCCTAAATGCATTGCTTGTTAACTGATTTATTCCATGACCTAGAGAAAGCTAGAGATGG
TTAGCCATAATTTATTGTTCTCTCTTGCTGCTACATTAAAACCAGCATCTTAAAGACTGGAATCTCTGGGATTCC

>Homo-sapiens_Gnathostomata_hg19_7-27098100-27098361_HOXA1_Matsunami10
GCTTTTAGATATTGAAATGGAGTCAATGGCATGCAAATGACACTTTAACACATTAAAGTATTTGGAAGCCAGCCATCTGTTTTGCTGTGTGGC
CCTACACTAATAAACAAAAGATACAGTATAAGGCCAGGCAGGAAATGAAAGAAAGGGTTGACCGTCTTTGAAGTTCTTCTCCATCCACTTGAG
TCTTTTACAGGCAACCTCAGAACCCAGCAGCTGAGGGGAGGGCACCCGGTGCCCTTGATATGGCCGCAGCAACTGC

>Homo-sapiens_Gnathostomata_hg19_7-27178987-27179128_HOXA10_Matsunami10
TCCCGCCCCTCAGCAACTTTGAAAAAAGCATGGGGGATCGTAAACTCGAACTTCGCCGGTTAATGGGCTTATTTATTGGCGCTGGCGGCTGCT
TATTTTGGATGCCTTACAAACATCCGCGCTATCTGCGGGCGAGCTACTT

>Homo-sapiens_Gnathostomata_hg19_7-27180486-27180716_HOXA10_Matsunami10
CCCCCCGCACCGCGGCGGGCGCCCGCAGCCAATCCCGAGCCAGAGTTTCCGCGCGACCACTCCCAGTTTGGTTTCGTAGGCGCGGGGCCGCTC
TCCGAGGGCGCCCTCAGAGCCCGCGATTGATATAAATATGTAATCTGTATTGATGGGCCAGGAGACGCACCCCGACACCTTGGCCCGAAGGCC
GGGAGCTGTGGGGGCTGCCCCAACGTGGCTGGTGGGGGGCCTGGC

>Homo-sapiens_Gnathostomata_hg19_7-27185928-27186077_HOXA10_Matsunami10
GAAGGGGAGCGGGACAAGTGAAATAATGTACCGTGCTGCTCTTAGTATCAGAAGCGAACAAAGGCCAAGAATCATGCTGGGGTTCCCGGCTCC
CCGGCGGCTTTGACATTGATCGGAAGTGCGCCATCTCGTGGCGGCTGCGCGCCTAGG

>Homo-sapiens_Gnathostomata_hg19_7-27186152-27186329_HOXA10_Matsunami10
ATGTGCCCAGCCCGCTGCTATTGAGATCTCATTTTTACATCTAAGAAATCGCTGCAAAACCCCAGCCGGGTTTATAGCGGCGCATTCCAAATA
TGCAAATTGGCCGGCCCCGGACGGGTTTACGACCACATTGTCACAGCCATCGGAGGATGGGCTTTTATAGGGCTCAGAAATCAAA

>Homo-sapiens_Gnathostomata_hg19_7-27186623-27186782_HOXA10-HOXA9_Matsunami10
CTCTCTCCAAATGCGACCCTATCTGCTGCTCTGGCCCTGCCTGGGTGGCTGAAGAGAGGGTGGGGTGGGCAACAAAGGGCTCTGTCCTTTCAG
CCCTTCTCCTCAAGGTTATGGGTGATGTCCAATTTTAAGGCAGAAGTTCAAAGGCAGCAAACAAAGA

>Homo-sapiens_Gnathostomata_hg19_7-27190015-27190357_HOXA11_Matsunami10
CCAGAGGAAAATGGCTTCCTTTGGACAAAAAGGCCAACTTTGGGTTAATTTGTTCTTTTAATATATTGCTAGAGCTAAGCGGGCTACTTTATC
TGTTAAACGCGCTCCTAGCGCCGTCGTTAAACAGCGACGCCTTTGAATCCCGGCCGGGACTGGAGTCCCGGCGCAACACATGGCTTTTATAAA
AATCTCCGGATTACCTCGCTATCAAAAGCTCCCGAAGCCCTTGCAGGGGGAATTTACAGGCGCCCACCTCCGGCTCCCCAGCCTGGAGCTGGC

CCCGAGCGGGGTCGAGCTGCTGGGCTTGGGAGCTGAGAAGGGAAAAAAGGGAAAAGGGGAGTTG

>Homo-sapiens_Gnathostomata_hg19_7-27190367-27190576_HOXA11_Matsunami10
GCAGGCATGCCTTGGCCGGTGGGTATTTCACGGCCAATTTCAGCACTCGCCACGTGATCCCGCCTTTTATAACAAAGTTTTGTTGGGGGAAAC
CTAAAGGCCCTTCATAAACCTTATATGCTTATAAAACAGCATATAAAAATTTAACAGCGGTGCTGCGCTAGATTTCCAACTCCCCTTTCATAA
AGCGCAGGGCGCTGCCTTTATACG

>Homo-sapiens_Gnathostomata_hg19_7-27191361-27191488_HOXA11_Matsunami10
CGGATCCGTGAAGTAGAAATTTGGAGACGTAAGCTGACGTGGAAATCTATCCCCATCCTTAGCAGGGAGGTGCTGGTCATGTGACCCGATGTT
GAAATTGACAAGCTGCTAGCTAGTCCGGGCCTTTT

>Homo-sapiens_Gnathostomata_hg19_7-27191521-27191674_HOXA11-AS_Matsunami10
CTCCCCTCCCTCCCGGCTTCCTTTCTTTGTAGCCACCTCAGGGGAAGCAACAGATCGTCACTCGGTGTTCTCACCGAAAGCACGTAATCGCCG
GTGTAACTCATGTTGGCTGGGGGGCCTCCCGGCGCGCGCGGAGAGGCTGGGGTGCGCCCCC

>Homo-sapiens_Gnathostomata_hg19_7-27191880-27192275_HOXA11-AS_Matsunami10
CCTTCTTCGCAGTCCCCCTCCCATAGACTTGCTCTGGGAAGCGCCTCTGCCTCCGACCCTAGCCGGAACCCCTTCGGGGCCAGAGTTTGAAGC
CGTGGATGTGCCTGCCTGGTGGCTTGTCCGATTTGCACGGTGACTTGATTACACTCTCTCATTCATGGTCACTTCCGAAGCGCTTTAGTGCCT
TCCGTCCCTAAACCGCCAACAGCCAGAACGGCTTCTCCCCGCGGTTTGTCACTGATCCGCAGGGCCCGGAAGGGCCTTCGTCTTACCCGGGAT
CCACCTCTCCCCTCATCTTCCCTGCCTACCTCTTCATCCCACCTTCTGTCCTTGGAGAAACTCCCTCCTCCTCGCTGCCTGCCGGGCTTCGGA
GTGACTCGGCAGAGACAGAGGCAC

>Homo-sapiens_Gnathostomata_hg19_7-27193021-27193315_HOXA11-AS_Matsunami10
CAGGAAATAGCCAAAGTTATTTACATATCTTGGGGAGATTTAGAGTATAAACTCTAAGATCTTTGGTATTTAAGTGTCAACATCGATTTATTT
ATTTATTGCTGAGCTGACTGTAACTGACTCAATAACAAATCTAATCGTGTATTGCACTGGAAAAGAAATATTCTTATTATGTATTTTCTCCAA
ATAATGGCCTACCATTGCATTTGAATACCTGCTGTAAATATCAATAATATGAAGTAATTACTCTGTAGTCGAGTAAACTAATTTATTAGCATT
AATGTTTATGTGGCTC

>Homo-sapiens_Gnathostomata_hg19_7-27193338-27193502_HOXA11-AS_Matsunami10
TTTACAAGGATTGCTTATCACAAATCAAGCTACTTGGACACATTGGTTTAATGAACTCTTTATTCAAGATTTGCTACAAGAATTTTCATGTCC
TTTGAATCCCTGAGAACTGAACTTGAAATTATTTGTGCATCTTCAGCTTGACATATTTGTCCACTGTGGCTT

>Homo-sapiens_Gnathostomata_hg19_7-27193783-27194044_HOXA11-AS_Matsunami10
GCAGCCCACCCAGTCCACGCCACCCCTACCTTCGAACAAAAGGAATGCATGAAGGGTTTCAGTGACTTTGCCATAACAAAGGCGCCACCATTG
CGGGGCTCGCCCCGCCCCTGGGTGAAGGCAAACAAATTCTTGCACTTGTATTAGGGCTTTTAAGACCATAATTGAACCCGGGGGCGTCTAGGA
AAACCGAAAACAGTTCTAGACAGACCTGCGGTTTTATAGCAGTTTTGGCAGTCAACTTCAGCTTGTGCCTGAGCAG

>Homo-sapiens_Gnathostomata_hg19_7-27194814-27195138_HOXA11-AS_Matsunami10
AAGGCCAAGTCCGAGTTCCATTTCTTGAAGAGGCCGGCGCGCGTAAGGCTGTGACATTGGCCCTGGCGACTGGCTTCCCAGGAGCTGTTCTTT
CTCAGGAGCTCCACAGCGCGGGCCATCTCCAGAAAACTGTCTTCAGAGTGTATTTCCTTTTATCGTCAACCCAGAGCCCCACCGCGGCTAATG
CAAGAGGCCAAAAAATGTTTGGAGGAAGAAAAACAAAGGCAGGAAGTGGCGGCGGCCTGACGGTGCGTGTGTGTCTGCAGAGAAGGGAGGGAG
CCGGCTCAGTCTCTTCTTGTTTTTCCAAACTTCAAGGTCCAGGCAG

>Homo-sapiens_Gnathostomata_hg19_7-27202498-27202765_HOXA13_Matsunami10
CAAGGAGGCTTGTCAACGCGAGGTGGCGCCCTTGATCTACTAATCCAGCTAAGGCCAATTCATGAGCTGTCAAAAGTCAAGGTCACAAATTGT
CTTTTTTTTCGTCAAGGTCAAGGTTTAAGGCCTTTCGTAGTCCTGTCATTTCAACAAATATCAAAACCTGCCCTCTCAGACACATGCAGACCC
AACAGCAGATTTTAAAATACGACAGCCTAGGCATTGCTGCTACAAAACAACTGGTTTTCATTTTCCACAGGGAGCCTGGGAA

>Homo-sapiens_HumanMouse_hg38_7-26981170-26981225_HOXA1_ANCORA
AAAGAAATTATTTAATTTAGAAAATATTTGATTTGGAATATTTAATATTGGAGTA

>Homo-sapiens_HumanMouse_hg38_7-26982201-26982305_HOXA1_ANCORA
ACAAGACAACCTGAAGCTAAATGGATGCCCACTGCAGAGTCAACAGGTCCAGCCTCACAGTGCATATCCTGAGCTACAGCCCCTTCAAAAGGC
ATCTTCCCCAT

>Homo-sapiens_HumanMouse_hg38_7-26983385-26983471_HOXA1_ANCORA
TAGTCTGGGCAGCGCAGGGCACAATGCATGGCTCAGTGCTCCCCAGCCTTGTGCTAGCTGCCTCCATGAGAGCCCGACTATCCTTT

>Homo-sapiens_HumanMouse_hg38_7-26988765-26988917_HOXA1_ANCORA
ACTTAGACATGCTAAATGGTGACCTAGGAGAATAATGTATGAGGACTGATGTCCCCTTTTGACCTAGAAGTGTACTTTTTAAACCAGGTTACT
TACTGCCCAAGGGTTCCATAGCTGGGGAAGATACAATGAGAAATTAAGTACATAACAAA

>Homo-sapiens_HumanMouse_hg38_7-26988926-26989484_HOXA1_ANCORA
GATGGTTATCATCACCTCAGCAAACCGCCGTACCCTCAGTACACAGTGCCCTCAGCCACCATCTCCAGATCAATATGGCTATCAATGACAGAT
GGCCAAAGCATCCCTATTCACTCAGGTCATTCTTAGTTCACACGGTCCGACAGGATTTAGGAGTCTTGTGTCATTAGACTGTCTTTAGAAAAC
AACCTAGCCATTAACGGCGGAAGCAGAGTAAAGTTGTATGTCAGACAGCTCACTGTTGACAGTTTAAATACACTCAGAAAGCTTAAAACATGT
TGATTTTCTGTGTACTACGATTACATTTTTACTGGCAGAGAACGTATCATTAAAACGTAAATGCATGTTGAAAGCAAAGCTATAAAAAGACTT
TTCTGTCACAAACCATTTCTAGACCCACCATAGCTAGGAAGAAAAAGTAATATTGTTCAGCACTGATTTGATCAAAATTGTAATTGCTGTGGA
AAGGAGAAGGGACTTTGGCTCAAATATAATTTTTAATATAGTTTATACTTCACAGAATCTGATTTGGGATCCATTTAGTTAAGAGACAGTCAC

>Homo-sapiens_HumanMouse_hg38_7-26989892-26990042_HOXA1_ANCORA
TTATTACTATCACAGGGTAAATTCCTTCTTGCTCAAGTACTACTGACTTCATTATCTGAGGATTGGACATGGCCCAAAGTTTATTAACTCACA
ATATTTTCCTTCACAGCAGTATTTCCATGAACAGTTGCTTTAAATTGAAAAAACAGA

>Homo-sapiens_HumanMouse_hg38_7-26990134-26990239_HOXA1_ANCORA
GTCACAAATACTCCATTTAGAAAGAGGCCTTAAATATTTTCTAGGAAGAATGAAAAATAATTTCTTTAGGGAGGAACATCCCTCAGTCAAATT
TCAAAAATAAAT

>Homo-sapiens_HumanMouse_hg38_7-26990280-26990393_HOXA1_ANCORA
ACAAGAGAGAGAACTCAAAAATAAAAGAATCTTGGCCTCTAGAAAAAGCAGTGGGAAACCTCTGGCCCTTAGTTTAATTTCTTCCAGCTACAC
CACGAAAAGTTTTCTAAAAC

>Homo-sapiens_HumanMouse_hg38_7-26990402-26990467_HOXA1_ANCORA
TTTGTGCCTCCCTTCTCTACCATTAAGAGCAACAAATACAGCTGTGCAAACAGCAGGAATGTGTG

>Homo-sapiens_Boreoeutheria_hg38_7-26990416-26990476_HOXA1_UCSC20200609
CTCTACCATTAAGAGCAACAAATACAGCTGTGCAAACAGCAGGAATGTGTGTTCCTGTGG

>Homo-sapiens_HumanMouse_hg38_7-26994725-26994776_HOXA1_ANCORA
CCCAGGACCCTCCAGAAAGTGCCACCCTTTCCCAGAGAGAACTCTTTAGGC

>Homo-sapiens_HumanMouse_hg38_7-26995456-26995549_HOXA1_ANCORA
GCTACCATACATTTCCTTTATGTAAATAAATAAATATTTCTAAATTGCTTCCATTAATGTATAGAAAAACATTTGTTATTTATTTGTAAATGG

>Homo-sapiens_HumanMouse_hg38_7-26995584-26995685_HOXA1_ANCORA
TTCTATAACAATTCTCTTAGTATATTATGTTTGAAAATGCAATATTTATATAGTAGAAACCATAATATTAAAACCCAACAAGCTTCTATAAGT
TTGTCCTT

>Homo-sapiens_HumanMouse_hg38_7-26995729-26995900_HOXA1_ANCORA
CTTTTAATGCCAATTCCATATCAAAGGTTAGGATTTTGCATTTATACAGAGCAGAGTTCATGAACTCCTGCTAATCATGTTAACAATCTACCA
GGTTTCTAAAGGCTCAGCAGGGGTTAGAATTAGAAGGCTCTGCCATCAACATCAATAACAGGAACAGTGACACTCAGA

>Homo-sapiens_HumanMouse_hg38_7-26995936-26996046_HOXA1_ANCORA
ACATTCAGGCACTGTAGCCCATAACTGAAGGAAATCTATAGTTAGTGATTCCAAAAGGGATCTAGAGGGACACATTATTTCAGATTTTGTTTT
AAATAGTGAGTCTTAAA

>Homo-sapiens_HumanMouse_hg38_7-26996330-26996405_HOXA1_ANCORA
CACATAAAAACAAAGTGAATACCTTTGAGTTTACAGATATACATTAAAAGCTAATTTCAAAGTATTTTTAAAAAT

>Homo-sapiens_HumanMouse_hg38_7-27010041-27010173_HOXA1_ANCORA
AAGAAGTATAACCATGGTAGTCATGGCAATTTACACTAGGAAGCTAAATTGTCTCTTTTCTAAATTACCATATGTACAAATTCTGCCTTTGGT
GAAAGTGCTATGTTACTTGCAATCCATGGAATAGCTATT

>Homo-sapiens_HumanMouse_hg38_7-27021467-27021519_HOXA1_ANCORA
TCGGGTCAAGAGATATAATTTAATTATGAGAAATTAATAAAGATATATGAAA

>Homo-sapiens_HumanMouse_hg38_7-27027106-27027169_HOXA1_ANCORA
TAACCTTAGAATGCCTAAGATGAAACTGTTCCTTGATACTGTCATTAAAAGAAGCATTTAATA

>Homo-sapiens_HumanMouse_hg38_7-27027199-27027286_HOXA1_ANCORA
TAATTTCTTTTTTAAACTCTTTACCCTTTGCTGAGGTTTTTTATTTGACATGTGTCCAAGGTAAAAAGACAAATCTACTCTTTTGCT

>Homo-sapiens_HumanMouse_hg38_7-27027358-27027549_HOXA1_ANCORA
GTTATATATGAGAATTGGTAGCAGTCTGTTGAAAGAAGTTTACCTGTGGTTAATAGCACTTATCTCACTTCTATCTATCATGCCATCAATTTC
TTTATTACAAGAGTTTACCTTTGGGCAGCTCATACACCAGCTGTCAATCTGTCCTCACCCCGAGACTTTGCAGTAAACCAAGCTCAGGTGTGT
TGAGG

>Homo-sapiens_HumanMouse_hg38_7-27027585-27027658_HOXA1_ANCORA
TAGAACAGGTGTAGTCAAAATGCAAGCCTCTGGCACTCCAGATGTGCCCCATCACAAGACAGAGCCAGAACTT

>Homo-sapiens_HumanMouse_hg38_7-27029304-27029372_HOXA1_ANCORA
AAAGAAAAATATTCATAAAGGAAATAGAAAGTGTGGTGTTTGCCAGAAAAAATTCAGAATTTAAAGCAT

>Homo-sapiens_HumanMouse_hg38_7-27030529-27030592_HOXA1_ANCORA
CTTAAAAGACCTCCCAGGGAGAGTAGCAACATTACAAAAACTCACACTGTTTCCCCTGTGGAG

>Homo-sapiens_HumanMouse_hg38_7-27030762-27031075_HOXA1_ANCORA
CTGTGGGAAACAGTGCAAGAACAAAAATAAGCAGGCAAGTGTTTGGGGCATTTCAGCTGGTAAGTGAGAGAGAGAAGCAGCCCCAAGACAGAT
GCAGTCCACGCCCTCCATCTGTAACCAGAGAGTGGCTCACATGTCAGAAAAAAGTTTGTCCTCCCTTAGGGGAATCTTGAAGGCTGTCAATGT
TCTAACCATGAGGAATCACAAAATCTATACGGAACATTCCAACATGTGCAGGCCTGATAGGTCTCAGAGCAATGTTCTATGGGAGTCTGAGTT
TTGCAGGCTTCTCACTTGGTGGACTGGGAATGTC

>Homo-sapiens_HumanMouse_hg38_7-27032915-27033095_HOXA1_ANCORA
CCTGTCACTTTAAGAATTTGTAATTTTTTCCATTAACATTCCTCTCTGGTTTTATGTACTTGGTCTGTGCTGTGGGATGTAGCATAAATTATC
TGCCTTCAGCAAAACGAGCAGCTGAAATGTGAGGCTGCCTGACAGTGCTTGTTAAGCCATTAGCTATTGTGAGCACACACTACTAGG

>Homo-sapiens_HumanMouse_hg38_7-27038125-27038210_HOXA1_ANCORA
GTGTTCAACAAGCCTAAGCATTTTTCTAGATCATCATCAAATGTCAAGGTTCACAAGTTTTTCAAATGTACTTAATGGTACTTAA

>Homo-sapiens_HumanMouse_hg38_7-27040299-27040370_HOXA1_ANCORA
ACAAACTAATATAGAAACCCTGTATCATTTTATTATAACTGTAAAGTTTGTCACAGAGTTCTTATGAGGAT

>Homo-sapiens_HumanMouse_hg38_7-27040775-27040861_HOXA1_ANCORA
CATTGTATATCTCTTTGCAATTATCTGCCACCTTAAAATGCTGTGGCTTGTAAGGCTGCCTCTCTCTTGGAATATTTACAAAAGGT

>Homo-sapiens_HumanMouse_hg38_7-27043102-27043222_HOXA1_ANCORA
TCAATTACAGTCTTATTCCTTCCCATTCTGCCAGTTCTGCACCAAGTGGCCTGTTCATACTTGACTCTATTCTGAAATGTCACTGAGAATTTT
TGTATTGCTGTTCTCCTTTCACAACTA

>Homo-sapiens_HumanMouse_hg38_7-27046857-27047031_HOXA1_ANCORA
CTTCCTTTTTAATGAGCTCTTCCTTAATCAAATCTTGGTTTAGCTTTGTATTGATTTTAGAACATGCTCCCTTTCCTATGCACATGTGTGTTT
TTCAAAGGCTGCAAAAGGTTAACAATTGTTATATATCATTATATACTAACAGTTGGTCTAATGACTAAGGATTGAATCAGC

>Homo-sapiens_HumanMouse_hg38_7-27047129-27047201_HOXA1_ANCORA
TATCTATAGACTATATTTATATTAAGACCTTTCAGTGAAAATGAGAAATGAATGTAAACTCATAAAGACCCA

>Homo-sapiens_HumanMouse_hg38_7-27049929-27049979_HOXA1_ANCORA
ACCTTCAAGCTGTTAAATTAGTGAATGGAGATCTATTATGACTTTAACAA

>Homo-sapiens_HumanMouse_hg38_7-27064323-27064374_HOXA1_ANCORA
TTTCAAAATGTCTAAACTGCTTCAACAGGAAGCCAGCCAAAGTTCTGTCCA

>Homo-sapiens_Boreoeutheria_hg38_7-27068562-27068613_HOXA1_UCSC20200609
AGCTGTTTGTAAAAGGTTATGAATCAGATTAAAATCCTCATTTGTCACAGA

>Homo-sapiens_HumanMouse_hg38_7-27068722-27068819_HOXA1_ANCORA
ATTAGAATACGCAGATCACTACCTGTTTCTCTCATAAAAAGCACAGCAAGGAATAAAAAACCACAAAATGCTATGCTGGGAAAAGGATGGGCT
TGAA

>Homo-sapiens_HumanMouse_hg38_7-27069036-27069085_HOXA1_ANCORA
AGACCTGGGAGTCCAGTTCCCATGGAACTGCCCATGGAACTCCCAAAGT

>Homo-sapiens_HumanMouse_hg38_7-27070574-27070659_HOXA1_ANCORA
GGTTAAAGAGCTACCAAAACAAAGCAAAGGTATATTTTATCATAAAATGCCACATGTGACTTTAAGCACTTTAGAGTATATCATT

>Homo-sapiens_HumanMouse_hg38_7-27071753-27071894_HOXA1_ANCORA
GTGCTCTGCAACAGATCTATCTCCTGCACCCAGCAGAGCTCAGGGTCATTCTTAGGTCATAGTGGTTAAAGACGGCTGTCAATTCACTGCTTG
GGCTAGGTTTCAAGTCTCCTTTATCTCTGTTATATTGATGATTCCCAG

>Homo-sapiens_HumanMouse_hg38_7-27071978-27072045_HOXA1_ANCORA
TGCTGTTATGTGAAAGTCGGCTACCCTTTAAAAACTAAGAACTGGAACAAATATATTTGAATAAATA

>Homo-sapiens_HumanMouse_hg38_7-27072093-27072163_HOXA1_ANCORA
AAAAGGTTGAAACTAGCTGATCCTGCCTCTTCTCAGTGCAAAGGTAATTATTATTCAGTGAAAAAATATG

>Homo-sapiens_HumanMouse_hg38_7-27072213-27072269_HOXA1_ANCORA
CACCTGGTGTTTTGATTTTTATGAATACCCTTTTGGATCCCCATGCCTGTGCAACT

>Homo-sapiens_HumanMouse_hg38_7-27072377-27072447_HOXA1_ANCORA
TCATTCCATTTAATTACTCTGATTTTGACAGGGACCTATTACAGATAAAATTGATAGCATGAGTAATAAG

>Homo-sapiens_HumanMouse_hg38_7-27072875-27072940_HOXA1_ANCORA
AATAGGATAGCCACCCTCAAATTGCTTCCTTCCAAGTTGAGTTATGTTTATGATATGACAGTGAT

>Homo-sapiens_HumanMouse_hg38_7-27073436-27073491_HOXA1_ANCORA
CATTAAACTATCTGCTACTTGAGTTCATTTTCCACATTCTAGTTTATGACTTACT

>Homo-sapiens_HumanMouse_hg38_7-27074098-27074451_HOXA1_ANCORA
ATTAAATCAATAGGCTATAAATTATATGCAAAAAAAAAAAAAATCAGGTCACTACCCTGACACAACAGATTTATTATTAAAGTAGGTAACTCCA
TCGCAATTCACATTAGTTTTTATAAAGTAAGAGACAGGGAGAGATCAATGAGAACTTTGGCTTACTGCTGCTTTTAATGGTTCATTTATAATA
GCTACTTATCTCCTATTACTTTAGCTTGCTATAATAATGCCAAGCCAAGAAAAAAGATAATCACCATTATGGGAATATATATTTAATTCAAGT
CTGATTGACTGTTCTTTAATCTGATTCATGTATTTGTAGAGTGGCCAGAAGTACTCTAAAAGCATAGTGATTCT

>Homo-sapiens_HumanMouse_hg38_7-27079752-27079818_HOXA1_ANCORA
CACTAAAACCCAAACACTAACTTCTCTCTGACCTCCACCCCCTGCAAAACAGAAAAGAAACTAGTA

>Homo-sapiens_HumanMouse_hg38_7-27080186-27080257_HOXA1_ANCORA
AGTAAGTTTTAGCAGTATGTTAATTCAAGCAATATTTACATTTTAAGCCATTTTATCCCTTATTGCATAAT

>Homo-sapiens_HumanMouse_hg38_7-27082551-27082624_HOXA1_ANCORA
TTTTATCTTGACTAATGTTTCATCTATACTATATATTTTGCATCAGAAATAAATTTTATGATTCTAGAAACAT

```
>Homo-sapiens_HumanMouse_hg38_7-27088535-27088589_HOXA1_ANCORA
ACAGCTGACCCCTGCGCATCCTAAAGGAAAGACCCCATCTGTTCCTCAGAATGG

>Homo-sapiens_HumanMouse_hg38_7-27088946-27089040_HOXA1_ANCORA
TTAAAAATAGAGACACGTGTTTTAGTTGTGTTTGAACATGTTGCAAAAAGAGACATTGGTCTTTTCATTTTTTCCAGTAAACTCAAAATGCAG
T

>Homo-sapiens_HumanMouse_hg38_7-27089283-27089345_HOXA1_ANCORA
ATCAGGTTCTGCTGAGATCAGTAGGACCCCATGGGGATGAGATGGAGGAGAATGTGGAAAGT

>Homo-sapiens_HumanMouse_hg38_7-27089761-27089811_HOXA1_ANCORA
AACAAGATGGCAGAAGCATTCAACTCAGAGGTTGTTTCAAAGATTCGGTA

>Homo-sapiens_HumanMouse_hg38_7-27090406-27090614_HOXA1_ANCORA
TTCTGCTTTAAGAATGATTATTCTTAAGGAGGAAACACAAACAGCAGTGACCATTTTTCACAGACGTGCTCAGCCATCAGGAAAGCTTAATAA
ATCCCTATTTAGTATGCATTTATGCAGTGATTACATGAACTTTGACCTCTGAAGAGCTGTAGCAATTAGCTTCAGTGTCTAGCATCTTCTACT
CACCATTCCCCACTGGCTTCCT

>Homo-sapiens_Boreoeutheria_hg38_7-27090487-27090553_HOXA1_UCSC20200609
AAAGCTTAATAAATCCCTATTTAGTATGCATTTATGCAGTGATTACATGAACTTTGACCTCTGAAG

>Homo-sapiens_HumanMouse_hg38_7-27090734-27090846_HOXA1_ANCORA
ACTCAACAGCCATCTCAGATGGATATTGGGGCTCTGATTGTCAATATTACTCAAACACAAATGGATTTCAATTCAATCTTGCCCTGTACTTTC
ACAAAGATAAACTCTAATG

>Homo-sapiens_HumanMouse_hg38_7-27090969-27091016_HOXA1_ANCORA
ATTGGCAGAAAACTAGAGGAGGGGGCAGGAGAAGGGCAGCTGGAAGC

>Homo-sapiens_HumanMouse_hg38_7-27091065-27091478_HOXA1_ANCORA
CCAAGAGGGGAGAGGAAAGTGTTTTAAGTTTCTAGTAAACATTGTTTAGCAAAAGAATAATAGGAAAGTTGCCCCTTTTCCAAAGAGGGATGG
AACTATAATTGTTGCCTTTAGTTGAGAGCTCAGATAAACTGCTGGGACTCATTCTAAAGTGACCCATCACTGCATTCATCTCTTCTTGAACTT
TCGGTGAACCTGAGATTAAACAGGGGTGAGCAGCTAAGCAGTTTGTTGTTAGCAGCCTTTTCAACAACCCTTTCAGACTGAGTAAATATTGAT
CGGTTTGAATCTGATTGCCCCAGAGGAAAACACCACCGCATTTGAATAGCCTCCAAAAGTAAACTTTAAAAGGTGAAACCCAGAGCAGACTTC
AGAGCCAAAGCAAGACCTCAACTCAGCCTAGTTTGGGAACA

>Homo-sapiens_Boreoeutheria_hg38_7-27091198-27091253_HOXA1_UCSC20200609
GCTGGGACTCATTCTAAAGTGACCCATCACTGCATTCATCTCTTCTTGAACTTTC

>Homo-sapiens_Boreoeutheria_hg38_7-27091254-27091385_HOXA1_UCSC20200609
GTGAACCTGAGATTAAACAGGGGTGAGCAGCTAAGCAGTTTGTTGTTAGCAGCCTTTTCAACAACCCTTTCAGACTGAGTAAATATTGATCGG
TTTGAATCTGATTGCCCCAGAGGAAAACACCACCGCAT

>Homo-sapiens_HumanMouse_hg38_7-27091569-27091633_HOXA1_ANCORA
TGCCTAACTGAGACCCTTGTTTCCTTTGTAAATGTATCAATAAAATGTTATTTTCAGTTTTCCC

>Homo-sapiens_HumanMouse_hg38_7-27091677-27091948_HOXA1_ANCORA
CTAACTGTGCTCCTTAGAGACCAAAGCAGAGAGACCTTGAAATAGGATGTGTTAAGCGCCTTTGATTTAATCGATTGAGACAGCTAAAAAGAT
ACAAATGTTTTCCTTGTTGAAAGGTGTATTAATGTTTCCTAAATGCATTGCTTGTTAACTGATTTATTCCATGACCTAGAGAAAGCTAGAGAT
GGTTAGCCATAATTTATTGTTCTCTCTTGCTGCTACATTAAAACCAGCATCTTAAAGACTGGAATCTCTGGGATTCCAGTGGATC

>Homo-sapiens_HumanMouse_hg38_7-27091955-27092217_HOXA1_ANCORA
GCTTTTAGATATTGAAATGGAGTCAATGGCATGCAAATGACACTTTAACACATTAAAGTATTTGGAAGCCAGCCATCTGTTTTGCTGTGTGGC
CCTACACTAATAAACAAAAGATACAGTATAAGGCCAGGCAGGAAATGAAAGAAAGGGTTGACCGTCTTTGAAGTTCTTCTCCATCCACTTGAG
TCTTTTACAGGCAACCTCAGAACCCAGCAGCTGAGGGGAGGGCACCCGGTGCCCTTGATATGGCCGCAGCAACTGC

>Homo-sapiens_HumanMouse_hg38_7-27092639-27092684_HOXA1_ANCORA
GGTTATGTGGCATGGAATTAGAACTGTGGATCCTTTACAGCCATG

>Homo-sapiens_HumanMouse_hg38_7-27092919-27092992_HOXA1_ANCORA
ATACTGGAGCTAATTAAATGTGAATCTCTTAGCTCTTGGTTCACTGATGTGAGGAAAGACTGACTGAAGACAG

>Homo-sapiens_HumanMouse_hg38_7-27167361-27167432_HOXA10-AS_ANCORA
GAAATGGCCCCGTCCTGTCCGCCTCATCTCCCTCCCCCTAATATTTTCCTTGCCCCATAAATTCCTCTAGG

>Homo-sapiens_HumanMouse_hg38_7-27172173-27172245_HOXA10_ANCORA
CTGGCATGTAAGAGAATAAAGAGGGGATGATTAAGTCGAGGCCACACGGGCTGCCCGCGGGGGTGAATTGCC

>Homo-sapiens_HumanMouse_hg38_7-27173106-27173290_HOXA10_ANCORA
TCCCTAGTCAGGGGGAGCTGAGGCCAGCGCCGAGGACGTCTTGCTGTGGGGCGCTAAGCCGGACATGAATTTTACTGCGTCCCCACGCCCAAA
TATTAAAAAGCAAGTTCACAAGGTCAGCCTGCCTGCAGCTTGGGCCAAGGCCGGCCGGCTGCTGCGCGGGCTCCTAGTTTTCTGATCCTTC

>Homo-sapiens_Boreoeutheria_hg38_7-27173175-27173251_HOXA10_UCSC20200609
TTTTACTGCGTCCCCACGCCCAAATATTAAAAAGCAAGTTCACAAGGTCAGCCTGCCTGCAGCTTGGGCCAAGGCC
```

```
>Homo-sapiens_HumanMouse_hg38_7-27174336-27174577_HOXA10_ANCORA
GCAATCCCCCCGCACCGCGGCGGGCGCCCGCAGCCAATCCCGAGCCAGAGTTTCCGCGCGACCACTCCCAGTTTGGTTTCGTAGGCGCGGGGC
CGCTCTCCGAGGGCGCCCTCAGAGCCCGCGATTGATATAAATATGTAATCTGTATTGATGGGCCAGGAGACGCACCCCGACACCTTGGCCCGA
AGGCCGGGAGCTGTGGGGGCTGCCCCAACGTGGCTGGTGGGGGGCCTGGCCATTG

>Homo-sapiens_Boreoeutheria_hg38_7-27174377-27174449_HOXA10_UCSC20200609
GAGCCAGAGTTTCCGCGCGACCACTCCCAGTTTGGTTTCGTAGGCGCGGGGCCGCTCTCCGAGGGCGCCCTC

>Homo-sapiens_HumanMouse_hg38_7-27174620-27174701_HOXA10_ANCORA
CCCTTAGAAGGGCCCTTGGGCCCCGCGCAGTTAACAAGTGGGGTGTTTATGGTGCGCGCCCAGTCTGCCTTGGGTGCTCAC

>Homo-sapiens_HumanMouse_hg38_7-27175930-27175980_HOXA10_ANCORA
CAGAGAGAGCTGGCTAGGTAGAGCTCCAGCTCTGGCTTCTGAGATTCAAG

>Homo-sapiens_HumanMouse_hg38_7-27176420-27176470_HOXA10_ANCORA
CCCTATCTTTAGCTTCCCGGATCTGCCTGGTCCTTACCCTGCTCACCAGA

>Homo-sapiens_HumanMouse_hg38_7-27176976-27177024_HOXA10_ANCORA
GGACTTGGGGGCCCTGCCAATTTGGCTGGCAAGACAAGCTGGGTGCCC

>Homo-sapiens_HumanMouse_hg38_7-27177601-27177676_HOXA10_ANCORA
AACACTGGAGTGTTAGGTTTCTCAATAAAATATTCCATTTATTGAGCGCCTCGGCTTTCCTGAGATTCCCCACCC

>Homo-sapiens_HumanMouse_hg38_7-27177810-27177861_HOXA10_ANCORA
CTTTGACCTAAATCTTCAGTGCCTGCTTGCCTCAACCAGGCCTTATGCAGC

>Homo-sapiens_HumanMouse_hg38_7-27179100-27179155_HOXA10_ANCORA
CTCAAAAATCACATGCGACTTCTCCGAATTGGCCTTTTTCGGAACAAAGGTTTTC

>Homo-sapiens_HumanMouse_hg38_7-27179243-27179294_HOXA10_ANCORA
CGGCCGCAGACTTCTACCACCCCATTGTTTAGAATGGAAACTTTGTTTTCC

>Homo-sapiens_HumanMouse_hg38_7-27179433-27179521_HOXA10_ANCORA
CTTGAGTGAAATGCTGGCGCAAGGAGCTAAAATCCTCAACTTTTCTACTTAGGCCTCCCCCTGCTTTCCAACCTTAGGGAGCAATGGG

>Homo-sapiens_HumanMouse_hg38_7-27179596-27179645_HOXA10_ANCORA
CCCACTCCCGCCCCACCAGACTGAAATTGCTAAACTTGTGGCCTCTTAC

>Homo-sapiens_HumanMouse_hg38_7-27180261-27180410_HOXA10-HOXA9_ANCORA
AATCTTTAACCATCCCAAAGGAAGTCTTTCCCTAAACCCAGGCTTCCCAGCCCGCCCCCTCCCTGCCCCCCAGGAGGGCCCTTGTTCATGTCT
GCGTGTCTGCCTATCAACCTAGACATTCATCTCTAGATCTGTCCCACTACCCTTTC

>Homo-sapiens_HumanMouse_hg38_7-27180469-27180658_HOXA10-HOXA9_ANCORA
GTCTGGGGCCTCTCTCCAAATGCGACCCTATCTGCTGCTCTGGCCCTGCCTGGGTGGCTGAAGAGAGGGTGGGGTGGGCAACAAAGGGCTCTG
TCCTTTCAGCCCTTCTCCTCAAGGTTATGGGTGATGTCCAATTTTAAGGCAGAAGTTCAAAGGCAGCAAACAAAGAGGAAATCGGCATCCTTT
TTT

>Homo-sapiens_HumanMouse_hg38_7-27181104-27181151_HOXA10-HOXA9_ANCORA
AGACATTTGGTAGTAAAAGGCGATCAGGGAGAGGAATGTCACACCAG

>Homo-sapiens_HumanMouse_hg38_7-27183116-27183171_HOXA11_ANCORA
GGGACTGGTGGTCCAGCCCAAGCCCCGTCAAAGTCTGCCCAGGCCCCTGCCTTTA

>Homo-sapiens_HumanMouse_hg38_7-27183199-27183450_HOXA11_ANCORA
GCAGTTGGAGGCTCAGCCACAGATAAAAGCCAGCTCCCTAAATAGGCCCCCGGGGAAGCTGCTCCCGCTGCCCCCAGAACAGAAAGGAGAGCT
TCGCACAGCAACAGGCGAGTTTGCGCTGCCTAAGCCTCTTTGAAAGCAGCCAAGTGGGGGTTGCCGGCTGCCTCTCGCAGGCCAGACTAAACA
AACAGCCGCCTGCGGACATTTCACCTTCCAGCACCTGGGCTGCCCCTTCCCAAGGCAGTAAAGGC

>Homo-sapiens_HumanMouse_hg38_7-27183465-27183552_HOXA11_ANCORA
CTTTCCCGGCTGCGGCCAAGACTGGGCAGTCCTTAACATCTGGGGCGGCTGCCGGGTCTTTGTCAGCCTGAGTCCTGGCCACCAGCT

>Homo-sapiens_HumanMouse_hg38_7-27183564-27183633_HOXA11_ANCORA
GCCAGCCTTCCGGCTCCGCCGAGGTGGGGAGGTGGCGGCGGAAGCCCCCTACCCTAGGCCTTCGCTGAG

>Homo-sapiens_Boreoeutheria_hg38_7-27184001-27184082_HOXA11_UCSC20200609
GCCTTTGAATCCCGGCCGGGACTGGAGTCCCGGCGCAACACATGGCTTTTATAAAAATCTCCGGATTACCTCGCTATCAAA

>Homo-sapiens_Boreoeutheria_hg38_7-27184222-27184385_HOXA11_UCSC20200609
GCAGGCATGCCTTGGCCGGTGGGTATTTCACGGCCAATTTCAGCACTCGCCACGTGATCCCGCCTTTTATAACAAAGTTTTGTTGGGGGAAAC
CTAAAGGCCCTTCATAAACCTTATATGCTTATAAAACAGCATATAAAAATTTAACAGCGGTGCTGCGCTA

>Homo-sapiens_BonyVertebrates_hg38_7-27184248-27184399_HOXA11_UCSC20200609
TTCACGGCCAATTTCAGCACTCGCCACGTGATCCCGCCTTTTATAACAAAGTTTTGTTGGGGGAAACCTAAAGGCCCTTCATAAACCTTATAT
GCTTATAAAACAGCATATAAAAATTTAACAGCGGTGCTGCGCTAGATTTCCAACTCCC
```

```
>Homo-sapiens_Amniota_hg38_7-27184315-27184375_HOXA11_UCSC20200609
CTAAAGGCCCTTCATAAACCTTATATGCTTATAAAACAGCATATAAAAATTTAACAGCGG

>Homo-sapiens_BonyVertebrates_hg38_7-27185244-27185300_HOXA11_UCSC20200609
GTAAGCTGACGTGGAAATCTATCCCCATCCTTAGCAGGGAGGTGCTGGTCATGTGA

>Homo-sapiens_Boreoeutheria_hg38_7-27185244-27185302_HOXA11_UCSC20200609
GTAAGCTGACGTGGAAATCTATCCCCATCCTTAGCAGGGAGGTGCTGGTCATGTGACC

>Homo-sapiens_BonyVertebrates_hg38_7-27185265-27185321_HOXA11_UCSC20200609
TCCCCATCCTTAGCAGGGAGGTGCTGGTCATGTGACCCGATGTTGAAATTGACAAG

>Homo-sapiens_HumanMouse_hg38_7-27187125-27187372_HOXA11-AS_ANCORA
TGTAGTCGAGTAAACTAATTTATTAGCATTAATGTTTATGTGGCTCTCCACCTCCCCCCACCACCACCTTTACAAGGATTGCTTATCACAAAT
CAAGCTACTTGGACACATTGGTTTAATGAACTCTTTATTCAAGATTTGCTACAAGAATTTTCATGTCCTTTGAATCCCTGAGAACTGAACTTG
AAATTATTTGTGCATCTTCAGCTTGACATATTTGTCCACTGTGGCTTGTCCAGAGGGCAGC

>Homo-sapiens_Boreoeutheria_hg38_7-27187303-27187358_HOXA11-AS_UCSC20200609
TGAACTTGAAATTATTTGTGCATCTTCAGCTTGACATATTTGTCCACTGTGGCTT

>Homo-sapiens_HumanMouse_hg38_7-27187457-27187526_HOXA11-AS_ANCORA
ACAGAATTGCCCTGCAGCGAAAAAACTTCTATTTCCAGCAGTGACCAACAAGGCAAAATGTTTGTTTCT

>Homo-sapiens_HumanMouse_hg38_7-27187600-27187818_HOXA11-AS_ANCORA
TCCCTGGCTTGATTTTGGCTATCCCAAGCTCTCTCTCTGCAGCCCACCCAGTCCACGCCACCCCTACCTTCGAACAAAAGGAATGCATGAAGG
GTTTCAGTGACTTTGCCATAACAAAGGCGCCACCATTGCGGGGCTCGCCCCGCCCCTGGGTGAAGGCAAACAAATTCTTGCACTTGTATTAGG
GCTTTTAAGACCATAATTGAACCCGGGGGCGT

>Homo-sapiens_Boreoeutheria_hg38_7-27187739-27187797_HOXA11-AS_UCSC20200609
GCCCCGCCCCTGGGTGAAGGCAAACAAATTCTTGCACTTGTATTAGGGCTTTTAAGAC

>Homo-sapiens_HumanMouse_hg38_7-27190269-27190347_HOXA11-AS_ANCORA
GTGGATTGCTTAGGGCACCATCATTATTTTGCCTCCTCCTGCTTGCCTAATCCCTTACTTTCCTCTGTCCACAAATGT

>Homo-sapiens_HumanMouse_hg38_7-27190903-27190964_HOXA11-AS_ANCORA
GCACAGGGCTCAGCTGAGGGTCTCAAGTACCTAAGCAATCTAGTTATCATTTACTTTAGTG

>Homo-sapiens_HumanMouse_hg38_7-27191574-27191625_HOXA11-AS_ANCORA
CTTAAGCTAACGTTTACCGCAGGGCCTGATGCTCTGATCTGCTGGAGGCAG

>Homo-sapiens_HumanMouse_hg38_7-27192195-27192249_HOXA11-AS_ANCORA
CGATCGGCCCTGACCATAGAGGCGGCCGTGGCGCGGAGGACTTGACCTTTACGG

>Homo-sapiens_HumanMouse_hg38_7-27192853-27193044_HOXA13_ANCORA
AGAAAAACACGGTTTCTTTTCAGCTCGTCTCAAAATCCCTTTTAGAGAAAATGCTCCTGTCAAGTTTTATTTCCCGTTGCAAACCACCTTCCA
CGCTGCCAAGAATTAAGACCGGGAGAGATTAAATACCCGATTATTCTCCTGGGGAGGGCGGGGCGGGGCCGGGAAGTGGGCACCCACACCAAA
CATTC

>Homo-sapiens_Boreoeutheria_hg38_7-27192930-27192994_HOXA13_UCSC20200609
TGCAAACCACCTTCCACGCTGCCAAGAATTAAGACCGGGAGAGATTAAATACCCGATTATTCTC

>Homo-sapiens_HumanMouse_hg38_7-27193637-27193913_HOXA13_ANCORA
ATTTTCATAAAGAATGAGGCCGGCTGTTATAGACCGGCGGCCTAGCAGATGAAAACTTAATTAGCGTGCCTGTCCTAAAACCTAGGCATAAAT
CTCCCTCTGCCTTTTGGATAACGCTATATCTTTGCTTATGAGAAATGGGATGTGAGCAACTCGCTGCACATTTCTCTGATTCTCCAGGTCTTG
GTCGGCTGACACGCATTCGATCAAGTTTAAAGGAATGCGCATAAATCAGCAAGCCCCTAGCGTCTCCTTGGGAGAGGTCCGCAAATCCAG

>Homo-sapiens_HumanMouse_hg38_7-27194244-27194359_HOXA13_ANCORA
TGGCTTCAGCTCTGCATCAGTCACAAATAGGAGTGAAATGCATAGCGACATTTAACAATCATCCACTTAAAATAAGTAAATAAATATGATAGT
ACTGAGAGCAGATAGAAAAAGT

>Homo-sapiens_HumanMouse_hg38_7-27194378-27194472_HOXA13_ANCORA
CCATTTTTATTTTCTTAATTCAGGAAGAGTTTTCTTTTTAGAAAAAAATACTTTAATCAGGCTTTCAACAACATTATCCATGGGTCAGTGGCT
G

>Homo-sapiens_HumanMouse_hg38_7-27198442-27198512_HOXA13_ANCORA
CTAGAAGACAAGGGAGAAGGCAAGTTACACAGGGATCGGACCCCAGCCAGGGCATAGGCGACAGCTCGAT
```

```
(A)
Your keyword:
7:27097212-27097599

Sequence was retrieved from :Homo_sapiens.GRCh38.dna_rm.primary_assembly.fa

>Homo-sapiens_GRCh38_7-27097212-27097599
ACCTTCCTCATTCTGCTGCACGTCTAGAGTGGGTGTGGGGGCTGGCAGGTGGGAGGGGCGGTGGACAAATGGCTGATGGTGGACGGGACA
CTTTACCCCAACGACACCTCCTCCCCTTTCCAACTGGCTGTGTAGTTGCTTATGAGAACCTTCAAGTCCTTCCCTAGAGAGACACATGCA
AATCTGAGCCTCATCCCAGGCCAGGGGTCCTGTTCCTCATCACCCTACTTCCCTGAGGCTGCTGAGGTCGTTAAATTGTTGTTTACTATT
AGGTTTCACGTCAACCCTGGGCTTGTAGAGAGAAAAAGCCAAACGGAGACCAAGAATTGATGCAGTCTTGGGTAGGAGAAATCGAGAGCT
TGTCCAGGAAGCTTTGCTGTATAAATTA


(B)
Your keyword:
11:31664397>A

Sequence with SNP was generated for :Homo_sapiens.GRCh38.dna_rm.primary_assembly.fa

>Homo-sapiens_GRCh38_11-31664297-31664497
AGATAAACTTTCCCATTGTCATTAATGAGGATGGATAATACTGAGTGACAAGTGCATTAGGAATGACACAGAAAGAGCTGTTAGATTTGTCAG
AGCGTATCACTTCAGGCATTGACACTTGAGGACATTCTTTTTGTGGATATTTGGTTNNNNNNNNAGTCTGATTTAAAGATACCAAAGTTTGTG
TTTGCTAATAGAATA
=>
>Homo-sapiens_GRCh38_11-31664297-31664497
AGATAAACTTTCCCATTGTCATTAATGAGGATGGATAATACTGAGTGACAAGTGCATTAGGAATGACACAGAAAGAGCTGTTAGATTTGTCAG
AGCGTATaACTTCAGGCATTGACACTTGAGGACATTCTTTTTGTGGATATTTGGTTNNNNNNNNAGTCTGATTTAAAGATACCAAAGTTTGTG
TTTGCTAATAGAATA
```

Fig. S3

(A) SIMO region

```
Download:result1534.zip

Query sequences:
>Homo-sapiens_GRCh38_11-31664297-31664497
AGATAAACTTTCCCATTGTCATTAATGAGGATGGATAATACTGAGTGACAAGTGCATTAGGAATGACACAGAAAGAGCTGTTAGATTTGT
CAGAGCGTATaACTTCAGGCATTGACACTTGAGGACATTCTTTTTGTGGATATTTGGTTNNNNNNNNAGTCTGATTTAAAGATACCAAAG
TTTGTGTTTGCTAATAGAATA

Human SNP in dbSNP:
11:31664297-31664497

# of blast hits:
Callorhinchus-milii               :1
Rhincodon-typus-N                 :1
Erpetoichthys-calabaricus         :1
Lepisosteus-oculatus              :1
Danio-rerio                       :1
Gasterosteus-aculeatus            :0
Tetraodon-nigroviridis            :0
Oryzias-latipes                   :0
Latimeria-chalumnae               :1
Microcaecilia-unicolor-N          :1
Xenopus-tropicalis                :1
Sphenodon-punctatus               :1
Podarcis-muralis-N                :1
Anolis-carolinensis               :1
Python-bivittatus-N               :1
Protobothrops-mucrosquamatus-N    :1
Thamnophis-sirtalis-N             :1
Pseudonaja-textilis-N             :1
Notechis-scutatus                 :1
Chelonoidis-abingdonii            :1
Alligator-mississippiensis-N      :1
Nothoprocta-perdicaria            :1
Gallus-gallus                     :1
Calidris-pugnax                   :1
Ornithorhynchus-anatinus          :1
Monodelphis-domestica             :1
Choloepus-hoffmanni               :0
Chrysochloris-asiatica-N          :1
Condylura-cristata-N              :1
Myotis-lucifugus                  :1
Equus-caballus                    :1
Bos-taurus                        :1
Heterocephalus-glaber-male        :1
Nannospalax-galili                :1
Mus-musculus                      :1
Propithecus-coquereli             :1
Aotus-nancymaae                   :1
Homo-sapiens                      :1

Alignment of blast hits:
121 sites (1) were used for tree reconstruction by excluding 86 sites (0).
speciesName_database_chr-start-end_geneName_bitScore

                                                                                  1
Used4treeSearch                                                                   111111111111111111111111111111111111111111111111111111111111111111100111111111111111111111
Callorhinchus-milii_3_KI635862.1-c11191492-11191364_ENSCMIG00000018323_155        AGATAAACTTTCCCATTGTCATTAATGAGGATGGGTAATACTGAGTGACAAGTGCATTTGGAATAGC--ACAGAAAGAGCTGCAAGGTTT
Rhincodon-typus-N_v2_NW018061796.1-136784-136905_NONE_145                         AGATAAACTTTCCCATTGTCATTAATGAGGATGGATAATACTGAGTGACAAGTGCATTTGCAATAGC--ACAGAAAGAGTTGCTCGGTTT
Erpetoichthys-calabaricus_1_2-56059158-56059220_ENSECRG00000004255_92             --ATAAACTTTCCCATTGTCATTAATGAGGATACATAATACTGAGTGACAAGTGAATTAAGATTG-------------------------
Lepisosteus-oculatus_LepOcu1_LG27-c9647340-9647199_elp4_157                       AGATAAACTTTCCCATTGTCATTAATGAGGATGGATAATACTGAGTGACAAGTGCATTAGGAATGG---GAAAAAGAGCTGTTAAATTT
Danio-rerio_GRCz11_25-14982965-14983072_pax6a_115                                 AGATAAACTTTCCCATTGTCATTAATGAGGACGGCTAATACTGAGTGACAAGTGCATTTGGAGAGGGA-GAAAAAGAGCCGTTAAATTT
Latimeria-chalumnae_LatCha1_JH126652.1-1144608-1144742_PAX6_186                   AGATAAACTTTCCCATTGTCATTAATGAGGATAGATAATACTGAGTGACAAGTGCATTAGGAGTGAC--ACAGAAAGAGCTGTTAGATTT
Microcaecilia-unicolor-N_aMicUni1-1_NC044034.1-c140995046-140994903_NONE_178      AGATAAACTTTCCCATTGTCATTAATGAGGATGAATAATCCTGAGTGACAAGTGCATTAGTAATGAC--ACTGAAAGATCTGTTGGATTT
Xenopus-tropicalis_v91_4-c9928179-9928050_pax6_163                                AGATAAACTTTCCCATTGTCATTAATGAGGATGGGTAATACTGAGTGACAAGTGTATTAGGCCTGAC--ACAGAGCGAGCTGATAGATTT
Sphenodon-punctatus_ASM311381v1_QEPC01008061.1-c1322829-1322700_ENSSPUG00000004916_217  AGATAAACTTTCCCATTGTCATTAATGAGGATGGATAATACTGAGTGACAAGTGCATTAGGAATGAC--ACAGAAAGAGCTGTTAGATTT
Podarcis-muralis-N_PodMur10_NC041312.1-66208970-66209091_NONE_185                 AGATAAACTTTCCCATTGTCATTAATGAGGATGGGTAATACTGAGTGACAAGTGCATTAGGAATGAC--ATAGAAAGAGCTGTTAGATTT
Anolis-carolinensis_0_1-60596126-60596247_PAX6_176                                AGATAAACTTTCCCATTGTCATTAATGAGGATGGGTAATACTGAGTGACAAGTGCATTAGGCATGAC--ATAGAAAGAGCTGTAAGATTT
Python-bivittatus-N_502_NW006532569.1-c336185-336041_NONE_181                     AGATAAACTTTCCCATTGTCCTTAATAAGTATGGGTAATACTGAGTGACAAGTGCATTAGGAATGAC--ACAGAAAGAGCTGTTAGATTT
Protobothrops-mucrosquamatus-N_1-0_NW015387874.1-c243457-243313_NONE_181          AGATAAACTTTCCCATTGTCCTTAATGAGTATGGGTAATACTGAGTGACAAGTGCATTAGGAATGAC--ACAGATAGAGCTGTTAGATTT
Thamnophis-sirtalis-N_6-0_NW013659983.1-c77921-77777_NONE_181                     AGATAAACTTTCCCATTGTCCTTAATGAGTATGGGTAATACTGAGTGACAAGTGCATTAGGAATGAC--ACAGAAAGAGCTGTTAGATTT
Pseudonaja-textilis-N_EBS10Xv2-PRI_NW020769309.1-c4740213-4740069_NONE_181        AGATAAACTTTCCCATTGTCCTTAATGAGTATGGGTAATACTGAGTGACAAGTGCATTAGGAATGAC--ACTGAAAGAGCTGTTAGATTT
Notechis-scutatus_TS10Xv2-PRI_ULFQ01000010.1-15211878-15212022_PAX6_181          AGATAAACTTTCCCATTGTCCTTAATGAGTATGGGTAATACTGAGTGACAAGTGCATTAGGAATGAC--ACTGAAAGAGCTGTTAGATTT
Chelonoidis-abingdonii_ASM359739v1_PKMU01008833.1-927903-928047_ELP4_222          AGATAAACTTTCCCATTGTCATTAATGAGGATGGATAATACTGAGTGACAAGTGCATTAGGAATGAC--ACAGAAAGAGCTGTTAGATTT
Alligator-mississippiensis-N_ASM28112v4_NW017713697.1-28306752-28306890_NONE_218  AGATAAACTTTCCCATTGTCATTAATGAGGATGGATAATACTGAGTGACAAGTGCATTAGGAATGAC--ACAGAAAGAGCTGTTAGATTT
Nothoprocta-perdicaria_notPer1_PTEW01000029.1-c3379258-3379125_PAX6_220          AGATAAACTTTCCCATTGTCATTAATGAGGATGGATAATACTGAGTGACAAGTGCATTAGGAATGAC--ACAGAAAGAGCTGTTAGATTT
Gallus-gallus_GRCg6a_5-5377906-5378040_ELP4_222                                   AGATAAACTTTCCCATTGTCATTAATGAGGATGGATAATACTGAGTGACAAGTGCATTAGGAATGAC--ACAGAAAGAGCTGTTAGATTT
Calidris-pugnax_ASM143184v1_KQ482227.1-1351623-1351757_IMMP1L_222                 AGATAAACTTTCCCATTGTCATTAATGAGGATGGATAATACTGAGTGACAAGTGCATTAGGAATGAC--ACAGAAAGAGCTGTTAGATTT
Ornithorhynchus-anatinus_OANA5_3-17794124-17794258_ENSOANG00000015151_217         AGATAAACTTTCCCATTGTCATTAATGAGGATGGATAATACTGAGTGACAAGTGCATTAGGAATGAC--ACAGAAAGAGCTGTTAGATTT
Monodelphis-domestica_monDom5_5-267266289-267266415_ELP4_155                      AGATAAGCTTTCCCATTGTCATTCATGAGGATGGATAATACTGAGTGACAAGTGCATTAGGAGTGACAGAGAGAGAGAGCTGTGAGATTT
Chrysochloris-asiatica-N_ChrAsi1-0_NW006408590.1-7043846-7044049_NONE_297         AGATAAACTTTCCCATTGTCATTAATGAGGATGGATAATACTGAGTGACAAGTGCATTAGGAATGAC--ACAGAAAGAGCGGTTAGATTT
Condylura-cristata-N_ConCri1-0_NW004567116.1-c18581419-18581232_NONE_275          AGATAAACTTTCCCATTGTCATTAATGAGGATGGATAATACTGAGTGACAAGTGCATTAGGAATGAC--ACAGAAAGAGCTGTTAGATTT
Myotis-lucifugus_0_GL429859-3566568-3566767_ELP4_289                              AGATAAACTTTCCCATTGTCATTAATGAGGATGGATAATACTGAGTGACAAGTGCATTAGGAATGAC--ACAGAAAGAGCTGTTAGATTT
Equus-caballus_0_7-99677548-99677745_PAX6_274                                     AGATAAACTTTCCCATTGTCATTAATGAGGATGGATAATACTGAGTGACAAGTGCATTAGGAATGAC--ACAGAAAGAGCTGTTACATTT
Bos-taurus_2_15-62436978-62437178_ELP4_306                                        AGATAAACTTTCCCATTGTCATTAATGAGGATGGATAATACTGAGTGACAAGTGCATTAGGAATGAC--ACAGAAAGAGCTGTTAGATTT
Heterocephalus-glaber-male_0_JH173915.1-573235-573378_ENSHGLG00100010470_193      AGATAAACTTTCCCATTGTCATTAATGAGGGTGGGTAATACTGAGTGACAAGTGCATTAGGAGTGAC--ACGGAGAGAGCTGTTAGATTT
Nannospalax-galili_0_KL199188.1-4838897-4839095_Elp4_298                          AGATAAACTTTCCCATTGTCATTAATGAGGATGGCTAATACTGAGTGACAAGTGCATTAGGAATGAC--ACAGAAAGAGCTGTTAGATTT
Mus-musculus_GRCm38_2-c105779564-105779372_Elp4_242                               AGATAAACTTTCCCATTGTCATTAATGAGGATGGATAATGCTGAGTGACAAGTGCATTAGGAATGAC--ACAGAGAGAGCTGTTAGATTT
Propithecus-coquereli_0_KQ022625.1-c5284583-5284383_PAX6_306                      AGATAAACTTTCCCATTGTCATTAATGAGGATGGATAATACTGAGTGACAAGTGCATTAGGAATGAC--ACAGAAAGAGCTGTTAGATTT
Aotus-nancymaae_0_KZ197012.1-6939453-6939641_PAX6_298                             AGATAAACTTTCCCATTGTCATTAATGAGGATGGATAATACTGAGTGACAAGTGCATTAGGAATGAC--ACAGAAAGAGCTGTTAGATTT
Homo-sapiens_GRCh38_11-31664297-31664497_PAX6_330                                 AGATAAACTTTCCCATTGTCATTAATGAGGATGGATAATACTGAGTGACAAGTGCATTAGGAATGAC--ACAGAAAGAGCTGTTAGATTT
YOURSEQ1_Homo-sapiens_GRCh38_11-31664297-31664497                                 AGATAAACTTTCCCATTGTCATTAATGAGGATGGATAATACTGAGTGACAAGTGCATTAGGAATGAC--ACAGAAAGAGCTGTTAGATTT

                                                                                  91
Used4treeSearch                                                                   111111111111111111111111111011111100000000000000000000000000000000000000000000000000000000
Callorhinchus-milii_3_KI635862.1-c11191492-11191364_ENSCMIG00000018323_155        GTCAAAGCATTTGCTTCAGGCATTG-ACTTAAGGCCT-TTC-------------------------------------------------
Rhincodon-typus-N_v2_NW018061796.1-136784-136905_NONE_145                         GTCAAAGCA-TTGCTTCAGGCATTG-ACTTAAGG--------------------------------------------------------
Erpetoichthys-calabaricus_1_2-56059158-56059220_ENSECRG00000004255_92             ------------------------------------------------------------------------------------------
Lepisosteus-oculatus_LepOcu1_LG27-c9647340-9647199_elp4_157                       GTCAAAGCAGATCATTTCAAGCAGTAAAACTTTAGGC---CTTTTTTGGTGTATTTTT--------------------------------
Danio-rerio_GRCz11_25-14982965-14983072_pax6a_115                                 GTCAAAGCAGATCATTTCA-----------------------------------------------------------------------
Latimeria-chalumnae_LatCha1_JH126652.1-1144608-1144742_PAX6_186                   GTCAAAGCACATTACTTCAGGCAATAAAACTTCAGGCCG-TTCCTTTT------------------------------------------
Microcaecilia-unicolor-N_aMicUni1-1_NC044034.1-c140995046-140994903_NONE_178      GTCAGAGCAAATCAGTTCAGGAGTGAAATTTAAGGACA-TTCTTTTTTGGTAATTT----------------------------------
Xenopus-tropicalis_v91_4-c9928179-9928050_pax6_163                                GTCAGAGCCTATCACTCCAAACAGTAGCACTTAAGGACA-TTC-----------------------------------------------
Sphenodon-punctatus_ASM311381v1_QEPC01008061.1-c1322829-1322700_ENSSPUG00000004916_217  GTCAGAGCATATCACTTCAGGCAATGAAACTTGAGGACA-TTC-----------------------------------------------
Podarcis-muralis-N_PodMur10_NC041312.1-66208970-66209091_NONE_185                 GTCAGAGCATATCACTTCAGGCTATGAAACCTGA--------------------------------------------------------
Anolis-carolinensis_0_1-60596126-60596247_PAX6_176                                GTCAGAGCATATCACTTCAGGCTATGAAACCTGA--------------------------------------------------------
Python-bivittatus-N_502_NW006532569.1-c336185-336041_NONE_181                     GTCAGAGCATATAACTTCAGGCTATGAAACCTGATGGCT-TTTTCTAGGAGTATATTT--------------------------------
Protobothrops-mucrosquamatus-N_1-0_NW015387874.1-c243457-243313_NONE_181          GTCAGAGCATATAACTTCAGGCTATGAAACCTGATGGCT-TTTTCTGGGAGTATATTT--------------------------------
Thamnophis-sirtalis-N_6-0_NW013659983.1-c77921-77777_NONE_181                     GTCAGAGTATATAACTTCAGGCTATGAAACCTGATGGCT-TTTTCTGGGAGTATATTT--------------------------------
Pseudonaja-textilis-N_EBS10Xv2-PRI_NW020769309.1-c4740213-4740069_NONE_181        GTCAGAGCATATAACTTCAGGCTATGAAACCTGATGGCT-TTTTCTGAGAGTATATTT--------------------------------
Notechis-scutatus_TS10Xv2-PRI_ULFQ01000010.1-15211878-15212022_PAX6_181          GTCAGAGCATATAACTTCAGGCTATGAAACCTGATGGCT-TTTTCTGAGAGTATATTT--------------------------------
Chelonoidis-abingdonii_ASM359739v1_PKMU01008833.1-927903-928047_ELP4_222          GTCAGAGCATATCACTTCTGGCAATGAAACTTGAGGACA-TTCTGTTTTGGTAATTT---------------------------------
Alligator-mississippiensis-N_ASM28112v4_NW017713697.1-28306752-28306890_NONE_218  GTCAGAGCATATCACTTCAGGCAATGAAACTTGAGGACA-TTTATCTTTTTG--------------------------------------
Nothoprocta-perdicaria_notPer1_PTEW01000029.1-c3379258-3379125_PAX6_220          GTCAGAGCATATCACTTCAGGCAATGAAACTTGAGGACA-TTCTCTT-------------------------------------------
Gallus-gallus_GRCg6a_5-5377906-5378040_ELP4_222                                   GTCAGAGCATATCACTTCAGGCAATGAAACTTGAGGACA-TTCTCTTT------------------------------------------
Calidris-pugnax_ASM143184v1_KQ482227.1-1351623-1351757_IMMP1L_222                 GTCAGAGCATATCACTTCAGGCAATGAAACTTGAGGACA-TTCTCTTT------------------------------------------
Ornithorhynchus-anatinus_OANA5_3-17794124-17794258_ENSOANG00000015151_217         GTCAGAGCACATCACTTCAGGCAAGGAAACTTGAGGACA-TTCTTTTT------------------------------------------
Monodelphis-domestica_monDom5_5-267266289-267266415_ELP4_155                      GTCAGAGCACATCACTTCAGTCACAGGAACTTGAGGA-----------------------------------------------------
Chrysochloris-asiatica-N_ChrAsi1-0_NW006408590.1-7043846-7044049_NONE_297         GTCAGAGCGTATCACTTCAGGCAGTGACACTTGAGGACA-TTGTTTTTGTGGATATTTGGTGAGTGAGAAGGGGGTCTGATTTAAAGATA
Condylura-cristata-N_ConCri1-0_NW004567116.1-c18581419-18581232_NONE_275          GTCAGAGCATATCACTTCAGGGAGTGACACTTGAGGACA-TTCTTTTTGTGGATATTTGATTGG---GGGGTAGTCTGATTTAAAGCTA
Myotis-lucifugus_0_GL429859-3566568-3566767_ELP4_289                              GTCAGAGTCTATCACTTCAGGCAGTGACACTTGAGGACATTCTTTTTGTGGATATTTGG-----GATGGGGGGTCTGATTTAAAGATA
Equus-caballus_0_7-99677548-99677745_PAX6_274                                     GTCAGAGCGTATCACTTCAGGCAGTGACACTTGAGGACA-TTCTTTTTGTGGATATTTGGTT----GAGGGGGGCTCTGTTTTAGAG--A
Bos-taurus_2_15-62436978-62437178_ELP4_306                                        GTCAGAGCATATCACTTCAGGCAGTGACACTTGAGGACA-TTCTTTTTGTGGATATTTGGTTGA--TGGGGGAGGTCTGATTTAAAGATA
Heterocephalus-glaber-male_0_JH173915.1-573235-573378_ENSHGLG00100010470_193      GTCAGAGCCCATCGCTTCAGGGCGTGACACTTGAGGACG-TTATTTCTGTGGATATT---------------------------------
Nannospalax-galili_0_KL199188.1-4838897-4839095_Elp4_298                          GTCAGAGCGGATCACTTCAGCCATTGACACTTGAGGACA-TTCTTTTTGTAGGTATTTGGTT-----GAGGGGAGTCTGATTTAAAGACA
Mus-musculus_GRCm38_2-c105779564-105779372_Elp4_242                               GTCAGAGCGTATCACTTCAGGCACTGACGCTCGACGACA-TTCCTTTTGAGAGTAT-----------GGGTGGGGCTGATTTAAAGATA
Propithecus-coquereli_0_KQ022625.1-c5284583-5284383_PAX6_306                      GTCAGAGCGTATCACTTCAGGCATTGACACTTGAGGACATTTCTTTTTGTGAATATTTGGTT----NNNNNNNAATCTGATTTTAAGATA
Aotus-nancymaae_0_KZ197012.1-6939453-6939641_PAX6_298                             GTCAGAGCGTATCACTTCAGGCATTGACACTTCAGGACA-TTCTTTTTGTGGATATTTGGTT-----GGGGGAGTCTGATTTAAAGATA
Homo-sapiens_GRCh38_11-31664297-31664497_PAX6_330                                 GTCAGAGCGTATCACTTCAGGCATTGACACTTGAGGACA-TTCTTTTTGTGGATATTTGGTTN---NNNNNNNAGTCTGATTTAAAGATA
YOURSEQ1_Homo-sapiens_GRCh38_11-31664297-31664497                                 GTCAGAGCGTATaACTTCAGGCATTGACACTTGAGGACA-TTCTTTTTGTGGATATTTGGTTN---NNNNNNNAGTCTGATTTAAAGATA

                                                                                  181
Used4treeSearch                                                                   000000000000000000000000000
Callorhinchus-milii_3_KI635862.1-c11191492-11191364_ENSCMIG00000018323_155        ---------------------------
Rhincodon-typus-N_v2_NW018061796.1-136784-136905_NONE_145                         ---------------------------
Erpetoichthys-calabaricus_1_2-56059158-56059220_ENSECRG00000004255_92             ---------------------------
Lepisosteus-oculatus_LepOcu1_LG27-c9647340-9647199_elp4_157                       ---------------------------
Danio-rerio_GRCz11_25-14982965-14983072_pax6a_115                                 ---------------------------
Latimeria-chalumnae_LatCha1_JH126652.1-1144608-1144742_PAX6_186                   ---------------------------
Microcaecilia-unicolor-N_aMicUni1-1_NC044034.1-c140995046-140994903_NONE_178      ---------------------------
Xenopus-tropicalis_v91_4-c9928179-9928050_pax6_163                                ---------------------------
Sphenodon-punctatus_ASM311381v1_QEPC01008061.1-c1322829-1322700_ENSSPUG00000004916_217  ---------------------------
Podarcis-muralis-N_PodMur10_NC041312.1-66208970-66209091_NONE_185                 ---------------------------
```

Fig. S4- 1

```
Anolis-carolinensis_0_1-60596126-60596247_PAX6_176                      ---------------------------
Python-bivittatus-N_502_NW006532569.1-c336185-336041_NONE_181           ---------------------------
Protobothrops-mucrosquamatus-N_1-0_NW015387874.1-c243457-243313_NONE_181 ---------------------------
Thamnophis-sirtalis-N_6-0_NW013659983.1-c77921-77777_NONE_181           ---------------------------
Pseudonaja-textilis-N_EBS10Xv2-PRI_NW020769309.1-c4740213-4740069_NONE_181 ---------------------------
Notechis-scutatus_TS10Xv2-PRI_ULFQ01000010.1-15211878-15212022_PAX6_181 ---------------------------
Chelonoidis-abingdonii_ASM359739v1_PKMU01008833.1-927903-928047_ELP4_222 ---------------------------
Alligator-mississippiensis-N_ASM28112v4_NW017713697.1-28306752-28306890_NONE_218 ---------------------------
Nothoprocta-perdicaria_notPer1_PTEW01000029.1-c3379258-3379125_PAX6_220 ---------------------------
Gallus-gallus_GRCg6a_5-5377906-5378040_ELP4_222                         ---------------------------
Calidris-pugnax_ASM143184v1_KQ482227.1-1351623-1351757_IMMP1L_222       ---------------------------
Ornithorhynchus-anatinus_OANA5_3-17794124-17794258_ENSOANG00000015151_217 ---------------------------
Monodelphis-domestica_monDom5_5-267266289-267266415_ELP4_155            ---------------------------
Chrysochloris-asiatica-N_ChrAsi1-0_NW006408590.1-7043846-7044049_NONE_297 CCAAAGTTTGTGTTTGCTAATAGAGTA
Condylura-cristata-N_ConCri1-0_NW004567116.1-c18581419-18581232_NONE_275 CTAAGGTTTGTGTT-------------
Myotis-lucifugus_0_GL429859-3566568-3566767_ELP4_289                    CCAAAGTTTGTGTTTGCTTATAGAATA
Equus-caballus_0_7-99677548-99677745_PAX6_274                           CCAAAGTTCACGTTTGCTAATAGAATA
Bos-taurus_2_15-62436978-62437178_ELP4_306                              CCAA-ATTTGTGTTTGCTAATAGAATA
Heterocephalus-glaber-male_0_JH173915.1-573235-573378_ENSHGLG00100010470_193 ---------------------------
Nannospalax-galili_0_KL199188.1-4838897-4839095_Elp4_298                CCAAAGTTTGTGTTTGCTAATAGAATA
Mus-musculus_GRCm38_2-c105779564-105779372_Elp4_242                     GTAAAGTTTATGTTTGCTAATAGAATA
Propithecus-coquereli_0_KQ022625.1-c5284583-5284383_PAX6_306            CCAAAGTTTGTGTTTGCTAATAGAATA
Aotus-nancymaae_0_KZ197012.1-6939453-6939641_PAX6_298                   CTAAAGTTTGTGTTTGC----------
Homo-sapiens_GRCh38_11-31664297-31664497_PAX6_330                       CCAAAGTTTGTGTTTGCTAATAGAATA
YOURSEQ1_Homo-sapiens_GRCh38_11-31664297-31664497                       CCAAAGTTTGTGTTTGCTAATAGAATA
```

CNS tree:

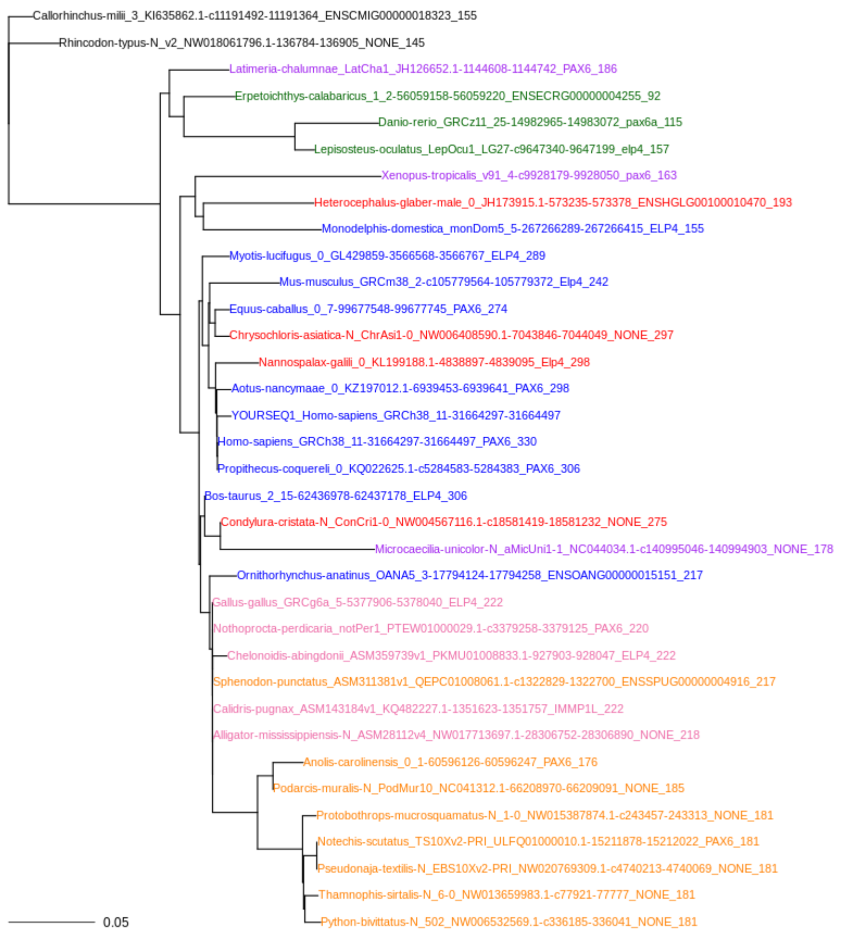

############### Settings ###############

Taxon sampling:
Callorhinchus-milii_Black
Rhincodon-typus-N_Black
Erpetoichthys-calabaricus_Green
Lepisosteus-oculatus_Green
Danio-rerio_Green
Gasterosteus-aculeatus_Green
Tetraodon-nigroviridis_Green
Oryzias-latipes_Green
Latimeria-chalumnae_Purple
Microcaecilia-unicolor-N_Purple
Xenopus-tropicalis_Purple
Sphenodon-punctatus_Orange
Podarcis-muralis-N_Orange
Anolis-carolinensis_Orange
Python-bivittatus-N_Orange
Protobothrops-mucrosquamatus-N_Orange
Thamnophis-sirtalis-N_Orange
Pseudonaja-textilis-N_Orange
Notechis-scutatus_Orange
Chelonoidis-abingdonii_Magenta
Alligator-mississippiensis-N_Magenta
Nothoprocta-perdicaria_Magenta
Gallus-gallus_Magenta
Calidris-pugnax_Magenta
Ornithorhynchus-anatinus_Blue
Monodelphis-domestica_Blue
Choloepus-hoffmanni_Blue
Chrysochloris-asiatica-N_Red
Condylura-cristata-N_Red
Myotis-lucifugus_Blue
Equus-caballus_Blue
Bos-taurus_Blue
Heterocephalus-glaber-male_Red
Nannospalax-galili_Red

Fig. S4- 2

```
Mus-musculus_Blue
Propithecus-coquereli_Blue
Aotus-nancymaae_Blue
Homo-sapiens_Blue

>blastn_task
blastn

>blastn_word_size
11

>blastn_E-value_threshold_for_reported_sequences
1e-3

>Number_of_hits_to_report_per_genome
2

>TreeSearchMethod
Neighbor-joining method (Saitou and Nei 1986)

>SubstitutionModel
TN93 (Tamura and Nei 1993) + gamma

>Dependencies
BLAST 2.7.1+
MAFFT v7.356b
trimAl 1.2rev59
ape in R, Version: 5.0

Analysis time: 36.6 seconds

Database:
Callorhinchus-milii              :Callorhinchus_milii.Callorhinchus_milii-6.1.3.dna_rm.toplevel.fa
Rhincodon-typus-N                :Rhincodon-typus-N_GCF_001642345.1_ASM164234.v2.dna_nb.genomic.fna
Erpetoichthys-calabaricus        :Erpetoichthys_calabaricus.fErpCal1.1.dna_rm.toplevel.fa
Lepisosteus-oculatus             :Lepisosteus_oculatus.LepOcu1.dna_rm.toplevel.fa
Danio-rerio                      :Danio_rerio.GRCz11.dna_rm.primary_assembly.fa
Gasterosteus-aculeatus           :Gasterosteus_aculeatus.BROADS1.dna_rm.toplevel.fa
Tetraodon-nigroviridis           :Tetraodon_nigroviridis.TETRAODON8.dna_rm.toplevel.fa
Oryzias-latipes                  :Oryzias_latipes.ASM223467v1.dna_rm.toplevel.fa
Latimeria-chalumnae              :Latimeria_chalumnae.LatCha1.dna_rm.toplevel.fa
Microcaecilia-unicolor-N         :Microcaecilia-unicolor-N.GCF_901765095-1.aMicUni1-1.dna_nb.genomic.fna
Xenopus-tropicalis               :Xenopus-tropicalis.v91.dna_rm.toplevel.fa
Sphenodon-punctatus              :Sphenodon_punctatus.ASM311381v1.dna_rm.toplevel.fa
Podarcis-muralis-N               :Podarcis-muralis-N.GCF_004329235-1.PodMur10.dna_nb.genomic.fna
Anolis-carolinensis              :Anolis_carolinensis.AnoCar2.0.dna_rm.toplevel.fa
Python-bivittatus-N              :Python-molurus-bivittatus-N.GCF_000186305-1_.502.dna_nb.genomic.fna
Protobothrops-mucrosquamatus-N   :Protobothrops-mucrosquamatus-N.GCF_001527695-2.1-0.dna_nb.genomic.fna
Thamnophis-sirtalis-N            :Thamnophis-sirtalis-N.GCF_001077635-1.6-0.dna_nb.genomic.fna
Pseudonaja-textilis-N            :Pseudonaja-textilis-N.GCF_900518735-1.EBS10Xv2-PRI.dna_nb.genomic.fna
Notechis-scutatus                :Notechis_scutatus.TS10Xv2-PRI.dna_rm.toplevel.fa
Chelonoidis-abingdonii           :Chelonoidis_abingdonii.ASM359739v1.dna_rm.toplevel.fa
Alligator-mississippiensis-N     :Alligator-mississippiensis-N.GCF_000281125-3.ASM28112v4.dna_nb.genomic.fna
Nothoprocta-perdicaria           :Nothoprocta_perdicaria.notPer1.dna_rm.toplevel.fa
Gallus-gallus                    :Gallus_gallus.GRCg6a.dna_rm.toplevel.fa
Calidris-pugnax                  :Calidris_pugnax.ASM143184v1.dna_rm.toplevel.fa
Ornithorhynchus-anatinus         :Ornithorhynchus_anatinus.OANA5.dna_rm.toplevel.fa
Monodelphis-domestica            :Monodelphis_domestica.monDom5.dna_rm.toplevel.fa
Choloepus-hoffmanni              :Choloepus_hoffmanni.choHof1.dna_rm.toplevel.fa
Chrysochloris-asiatica-N         :Chrysochloris-asiatica-N.GCF_000296735-1.ChrAsi1-0.dna_nb.genomic.fna
Condylura-cristata-N             :Condylura-cristata-N.GCF_000260355-1.ConCri1-0.dna_nb.genomic.fna
Myotis-lucifugus                 :Myotis_lucifugus.Myoluc2.0.dna_rm.toplevel.fa
Equus-caballus                   :Equus_caballus.EquCab3.0.dna_rm.toplevel.fa
Bos-taurus                       :Bos_taurus.ARS-UCD1.2.dna_rm.toplevel.fa
Heterocephalus-glaber-male       :Heterocephalus_glaber_male.HetGla_1.0.dna_rm.toplevel.fa
Nannospalax-galili               :Nannospalax_galili.S.galili_v1.0.dna_rm.toplevel.fa
Mus-musculus                     :Mus_musculus.GRCm38.dna_rm.primary_assembly.fa
Propithecus-coquereli            :Propithecus_coquereli.Pcoq_1.0.dna_rm.toplevel.fa
Aotus-nancymaae                  :Aotus_nancymaae.Anan_2.0.dna_rm.toplevel.fa
Homo-sapiens                     :Homo_sapiens.GRCh38.dna_rm.primary_assembly.fa
```

(B) Nasopharyngeal carcinoma-related SNP

Download:result2116.zip

Query sequences:
>Homo-sapiens_GRCh38_3-169364745-169364945
TGACATCACCTGTTCACCCACTCAAAATTGATGATGAAAGCAAACAGATCTATCTTACTTACATTTACCAAATCAATTTAATTTTTACAG
GCCATGGCTTaGTGACATATTGACTGTTTTCCTTAATGGCATCTACCGTATTTAATGCAAGGATGTCATCTTTGATTCCTGGCTTGCATA
ATGTGTCATTTCATAATAAAT

Human SNP in dbSNP:
3:169364745-169364945

```
# of blast hits:
Callorhinchus-milii              :1
Rhincodon-typus-N                :1
Erpetoichthys-calabaricus        :1
Lepisosteus-oculatus             :1
Danio-rerio                      :1
Gasterosteus-aculeatus           :1
Tetraodon-nigroviridis           :1
Oryzias-latipes                  :2
Latimeria-chalumnae              :1
Microcaecilia-unicolor-N         :1
Xenopus-tropicalis               :1
Sphenodon-punctatus              :1
Podarcis-muralis-N               :1
Anolis-carolinensis              :1
Python-bivittatus-N              :1
Protobothrops-mucrosquamatus-N   :1
Thamnophis-sirtalis-N            :1
Pseudonaja-textilis-N            :1
Notechis-scutatus                :1
Chelonoidis-abingdonii           :1
Alligator-mississippiensis-N     :1
Nothoprocta-perdicaria           :1
Gallus-gallus                    :1
Calidris-pugnax                  :1
Ornithorhynchus-anatinus         :1
Monodelphis-domestica            :1
Choloepus-hoffmanni              :1
Chrysochloris-asiatica-N         :1
Condylura-cristata-N             :1
Myotis-lucifugus                 :1
Equus-caballus                   :1
Bos-taurus                       :1
Heterocephalus-glaber-male       :1
Nannospalax-galili               :1
Mus-musculus                     :1
Propithecus-coquereli            :1
Aotus-nancymaae                  :1
Homo-sapiens                     :1
```

Alignment of blast hits:
192 sites (1) were used for tree reconstruction by excluding 27 sites (0).
speciesName_database_chr-start-end_geneName_bitScore

```
                                                                                     1
Used4treeSearch                                                                      111111111111111110111111111111111111111111111111111111111101111111111111111111100111110111
Callorhinchus-milii_3_KI635855.1-c6442281-6442076_ENSCMIG00000008884_190             TGACATCACCTCTTCTC-CTCTCCAAAATGGATGATGAAAGCAAACAGATCTATCTTG-CTTACATTTACCAAATCAA--TTTAA-TTTT
Rhincodon-typus-N_v2_NW018043067.1-26837-27027_NONE_182                              TGACATCACCTGTTCTC-CCATCTGAAATTGATGATGAAAGCAAACAGATCTATCTTG-CTTACATTTACCAAATCAA--TTTAA-TTTT
Erpetoichthys-calabaricus_1_2-138640288-138640492_ENSECRG00000013444_203             TGACATCACCTGGTCTC-CCACTCAGAACTGATGATGAAAGCAAACAGATCTATCTTG-CTTACATTTACCAAATCAA--TTTTATTTTA
Lepisosteus-oculatus_LepOcu1_LG14-c19988744-19988541_ENSLOCG00000009550_198          TGACATCACCTGGTCTC-CCACTCAGAATTGATGATGAAAGCAAACAGATCTATCTTG-CTTACATTTACCAAATCAANNNNNNNNNNNN
Danio-rerio_GRCz11_15-35542490-35542588_mecom_108                                    -----------------------CAGAACTGATGATGAAAGCAAACAGATCTATCTTAACTCGCATTTACCAAATCAA--TTTTATTTTT
Gasterosteus-aculeatus_BROADS1_groupI-c5410722-5410623_ENSGACG00000007274_97         --------------------------AATTGATGATGAAAGCAAACAAATCTGTCTTAACTCACGTATACCAAATCAA--TTTTGTTTTT
Tetraodon-nigroviridis_TETRAODON8_Unrandom-c54161107-54161030_ENSTNIG00000002725_58  ------------------------------------------------------------------------------------------
Oryzias-latipes_ASM223467v1_13-23170268-23170388_mecom_104                           TGACATCACCCCGCCACGCTGCACAGAATTGATGATGAAAGCAAACATATCTGTCTTAACTTGCATTTACCAAATCAA--TTTTATTTTT
Oryzias-latipes_ASM223467v1_13-23170479-23170561_mecom_67                            ------------------------------------------------------------------------------------------
Latimeria-chalumnae_LatCha1_JH126697.1-c1747311-1747112_ENSLACG00000016795_265       TGACATCACCTGTTCAC-CCACTCAGAATTGATGATGAAAGCAAACAGATCTATCTTG-CTTACATTTACCAAATCAA--TTTAA-TTTT
Microcaecilia-unicolor-N_aMicUni1-1_NC044040.1-195525342-195525529_NONE_270          TGACATCACCTGTTCAC-CCACTCAAAATTGATGATGAAAGCAAACAGATCTATCTCA-CTTACATTTACCAAATCAA--TTTAA-TTTT
Xenopus-tropicalis_v91_KV460428.1-c193815-193644_erp29_120                           ----------------C-CTCTCAGAATTTGATGATGAAAATAAACCAGTGTTTCTCA-CTTACATTTACCAAATCAA--TTTAA-TTTT
Sphenodon-punctatus_ASM311381v1_QEPC01014730.1-c6120370-6120172_MECOM_320            TGACATCACCTGTTCA--CCACTCAAAATTGATGATGAAAGCAAACAGATCTATCTTA-CTTACATTTACCAAATCAA--TTTAA-TTTT
Podarcis-muralis-N_PodMur10_NC041316.1-84176510-84176708_NONE_295                    TGACATCACCTGTTCAC-CCCCTCAAAATTGATGATGAAAGCAAACAGATCTATCTTA-CTTACATTTACCAAATCAA--TTTAA-TTTT
Anolis-carolinensis_0_3-c8300518-8300320_ENSACAG00000015690_286                      TGACATCACCTGTTCAC-CCCCTCAAAATTGATGATGAAAGCAAACAGATCTATCTTA-CTTACATTTACCAAATCAA--TTTAA-TTTT
Python-bivittatus-N_502_NW006534654.1-166579-166775_NONE_299                         TGACATCACCTGTTCAC-CCCCTCAAAATTGATGATGAAAGCAAACAGATCTATCTTA-CTTACATTTACCAAATCAA--TTTAA-TTTT
Protobothrops-mucrosquamatus-N_1-0_NW015387498.1-c242829-242632_NONE_298             TGACATCACCTGTTCAC-CCCCTCAAAATTGATGATGAAAGCAAACAGATCTATCTTA-CTTACATTTACCAAATCAA--TTTAA-TTTT
Thamnophis-sirtalis-N_6-0_NW013657765.1-c228880-228683_NONE_298                      TGACATCACCTGTTCAC-CCCCTCAAAATTGATGATGAAAGCAAACAGATCTATCTTA-CTTACATTTACCAAATCAA--TTTAA-TTTT
Pseudonaja-textilis-N_EBS10Xv2-PRI_NW020769337.1-c8326428-8326231_NONE_303           TGACATCACCTGTTCAC-CCCCTCAAAATTGATGATGAAAGCAAACAGATCTATCTTA-CTTACATTTACCAAATCAA--TTTAA-TTTT
Notechis-scutatus_TS10Xv2-PRI_ULFQ01000001.1-43599067-43599263_ENSNSUG00000002523_301 TGACATCACCTGTTCAC-CCCCTCAAAATTGATGATGAAAGCAAACAGATCTATCTTA-CTTACATTTACCAAATCAA--TTTAA-TTTT
Chelonoidis-abingdonii_ASM359739v1_PKMU01001678.1-c2481550-2481351_ENSCABG00000009144_297 TGACATCACCTGTTCAT-CCACTCAAAATTGATGATGAAAGCAAACAGATCTATCTTA-CTTACATTTACCAAATCAA--TTTAA-TTTT
Alligator-mississippiensis-N_ASM28112v4_NW017711041.1-26335021-26335220_NONE_328     TGACATCACCTGTTCAC-CCACTCAAAATTGATGATGAAAGCAAACAGATCTATCTTA-CTTACATTTACCAAATCAA--TTTAA-TTTT
Nothoprocta-perdicaria_notPer1_PTEW01000005.1-3871716-3871914_ENSNPEG00000002221_299 TGACATCACCTGTTCAC-CCACTCAAAATTGATGATGAAAGCAAACAGATCTATCTTA-CTTACATTTACCAAATCAA--TTTAA-TTTT
Gallus-gallus_GRCg6a_9-c20276997-20276798_MECOM_310                                  TGACATCACCTGTTCAC-CCACTCAAAATTGATGATGAAAGCAAACAGATCTATCTTA-CTTACATTTACCAAATCAA--TTTAA-TTTT
Calidris-pugnax_ASM143184v1_KQ482134.1-1573146-1573344_MECOM_308                     TGACATCACCTGTTCAC-CCACTCAAAATTGATGATGAAAGCAAACAGATCTATCTTA-CTTACATTTACCAAATCAA--TTTAA-TTTT
Ornithorhynchus-anatinus_OANA5_Contig328-c389526-389326_ENSOANG00000007584_323       TGACATCACCTGTTCGC-CCGTTCAAAATTGATGATGAAAGCAAACAGATCTATCTTA-CTTACATTTACCAAATCAA--TTTAATTTTT
Monodelphis-domestica_monDom5_7-c233068836-233068636_ENSMODG00000024658_291          TGACATCACCTGTTCAC-CCACTCAAAATTGATGATGAAAGCAAACAGATCTATCTTA-CTTACATTTACCAAATCAA--TTTAATTTTT
Choloepus-hoffmanni_choHof1_scaffold1067-25174-25374_ENSCHOG00000003815_345          TGACATCACCTGTTCAC-CCACTCAAAATTGATGATGAAAGCAAACAGATCTATCTTA-CTTACATTTACCAAATCAA--TTTAATTTTT
Chrysochloris-asiatica-N_ChrAsi1-0_NW006408561.1-16774181-16774381_NONE_345          TGACATCACCTGTTCAC-CCACTCAAAATTGATGATGAAAGCAAACAGATCTATCTTA-CTTACATTTACCAAATCAA--TTTAATTTTT
Condylura-cristata-N_ConCri1-0_NW004567104.1-7625208-7625408_NONE_350                TGACATCACCTGTTCAC-CCACTCAAAATTGATGATGAAAGCAAACAGATCTATCTTA-CTTACATTTACCAAATCAA--TTTAATTTTT
Myotis-lucifugus_0_GL429820-c3017858-3017658_ENSMLUG00000015759_354                  TGACATCACCTGTTCAC-CCACTCAAAATTGATGATGAAAGCAAACAGATCTATCTTA-CTTACATTTACCAAATCAA--TTTAATTTTT
Equus-caballus_0_19-12246137-12246337_MECOM_350                                      TGACATCACCTGTTCAC-CCACTCAAAATTGATGATGAAAGCAAACAGATCTATCTTA-CTTACATTTACCAAATCAA--TTTAATTTTT
Bos-taurus_2_1-c98035718-98035518_ENSBTAG00000047144_345                             TGACATCACCTGTTCAC-CCACTCAAAATTGATGATGAAAGCAAACAGATCTATCTTA-CTTACATTTACCAAATCAA--TTTAATTTTT
Heterocephalus-glaber-male_0_JH173818.1-448008-448186_ENSHGLG00100021283_319         TGACATCACCTGTTCAC-CCACTCAAAATTGATGATGAAAGCAAACAGATCTATCTTA-CTTACATTTACCAAATCAA--TTTAATTTTT
Nannospalax-galili_0_KL200152.1-c2486863-2486663_ENSNGAG00000001009_345              TGACATCACCTGTTCAC-CCACTCAAAATTGATGATGAAAGCAAACAGATCTATCTTA-CTTACATTTACCAAATCAA--TTTAATTTTT
Mus-musculus_GRCm38_3-30229286-30229486_Mecom_350                                    TGACATCACCTGTTCAC-CCACTCAAAATTGATGATGAAAGCAAACAGATCTATCTTA-CTTACATTTACCAAATCAA--TTTAATTTTT
Propithecus-coquereli_0_KQ018509.1-3029968-3030168_MECOM_350                         TGACATCACCTGTTCAC-CCACTCAAAATTGATGATGAAAGCAAACAGATCTATCTTA-CTTACATTTACCAAATCAA--TTTAATTTTT
Aotus-nancymaae_0_KZ197721.1-c792585-792385_MECOM_350                                TGACATCACCTGTTCAC-CCACTCAAAATTGATGATGAAAGCAAACAGATCTATCTTA-CTTACATTTACCAAATCAA--TTTAATTTTT
Homo-sapiens_GRCh38_3-169364745-169364945_MECOM_359                                  TGACATCACCTGTTCAC-CCACTCAAAATTGATGATGAAAGCAAACAGATCTATCTTA-CTTACATTTACCAAATCAA--TTTAATTTTT
YOURSEQ1_Homo-sapiens_GRCh38_3-169364745-169364945                                   TGACATCACCTGTTCAC-CCACTCAAAATTGATGATGAAAGCAAACAGATCTATCTTA-CTTACATTTACCAAATCAA--TTTAATTTTT

                                                                                     91
Used4treeSearch                                                                      111111111000001111111111111111111110111111111111111111111111011111111111000011111111111111
Callorhinchus-milii_3_KI635855.1-c6442281-6442076_ENSCMIG00000008884_190             ACAAG-CCAA----GTCTTGGTGGCATATTGACTGTTCTGTGTTAATGGCATCTGCTGTG-TTTAGTGCTGGCTGTACAGTCCTCTTTGA
Rhincodon-typus-N_v2_NW018043067.1-26837-27027_NONE_182                              ACAGGCCCCA----GCGTTGGTGGCATATTGACAG-TCTGTGTTAATGGCATCTACTGTG-TTTAGTGCCTG--GAAGAGTCCTCCTTGA
Erpetoichthys-calabaricus_1_2-138640288-138640492_ENSECRG00000013444_203             ACAGGCCTC-----TGCATATTGGCATATTGACTG-TTGCTCTTAATAGCATTTACCATATTTTAATGCAAA----GATGTCATCACTGA
Lepisosteus-oculatus_LepOcu1_LG14-c19988744-19988541_ENSLOCG00000009550_198          ACAGGCCCC-----TGCATATTGGCATATTGACTG-TTCGCCTTAATGGCATTTGCCATA-TTTAATGCAAG----GATGTCATCACTGA
Danio-rerio_GRCz11_15-35542490-35542588_mecom_108                                    ACAGTCTCTT-----GCATATTGGCATATTGACGG-------------------------CTTT--------------------------
Gasterosteus-aculeatus_BROADS1_groupI-c5410722-5410623_ENSGACG00000007274_97         ACAGACCCTCCGCGGGCTTATTGGCATATTGACGG-------------------------TTT---------------------------
Tetraodon-nigroviridis_TETRAODON8_Unrandom-c54161107-54161030_ENSTNIG00000002725_58  ----------------------------------------------TGGCATTTACCATA-TTTAATGCAGC--GGGATGTCATCACTGA
Oryzias-latipes_ASM223467v1_13-23170268-23170388_mecom_104                           ACAGACCCTCCTCAGACTTATTGGCATATTGAC---------------------------------------------------------
Oryzias-latipes_ASM223467v1_13-23170479-23170561_mecom_67                            -----------------------------------------CTTAATGGCATTTACCATA-TTTAATGCCGA--GGGATGTCATCACTGA
Latimeria-chalumnae_LatCha1_JH126697.1-c1747311-1747112_ENSLACG00000016795_265       ACAGGCCAC-----AGCGCAGTGACATATTGACTG-CTCTCCTTAATGGCATATACTGTA-GTTAATGCAAG----GATGTCATCTCGGA
Microcaecilia-unicolor-N_aMicUni1-1_NC044040.1-195525342-195525529_NONE_270          ACAGGCCATA----GGCTTAGTGACATATTGACTG-TTTTCCTTAATGGCATCTACTGTA-CTTAATGCA-G----AATGTCATCTTT--
Xenopus-tropicalis_v91_KV460428.1-c193815-193644_erp29_120                           ACAGGCTTCTGT--------GTGACTTATTGATTG-TTTCATTTAATAGCATCCACTGT--TTTAATGAAGG----TAAGTCATCTTTGA
Sphenodon-punctatus_ASM311381v1_QEPC01014730.1-c6120370-6120172_MECOM_320            ACAGGCCGT-----GGCTTAGTGACATATTGACTG-TTTTCCTTAATGGCATCTACTGTA-CTTAATGCAAG----GAACTCATCTTTGA
Podarcis-muralis-N_PodMur10_NC041316.1-84176510-84176708_NONE_295                    ACAGGCTGT-----GGCTTAGTGACATATTGACTG-TTTTCCTTAATGGCATCTACTATA-CTTAACGTAAG----GAACTCATCTTTGA
Anolis-carolinensis_0_3-c8300518-8300320_ENSACAG00000015690_286                      ACAGGCTGT-----GGCTTAGTGACATATTGACTG-TTTTCCTTAATGGCATTTACTGTA-CTTAACGCAAA----GAACTCATCTTTGA
Python-bivittatus-N_502_NW006534654.1-166579-166775_NONE_299                         ACAGGCTGT-----GGCTTAGTGACATATTGACTG-TTTTCCTTAATGGCATCTACCGTA-CTTAAAGCAAG----GAACTCATCTTTGA
Protobothrops-mucrosquamatus-N_1-0_NW015387498.1-c242829-242632_NONE_298             ACAGGCTGT-----GGCTTAGTGACATATTGACTG-TTTTCCTTAATGGCATCTACTGTA-CTTAAAGCAAG----GAACTTATCTTTGA
Thamnophis-sirtalis-N_6-0_NW013657765.1-c228880-228683_NONE_298                      ACAGGCTGT-----GGCTTAGTGACATATTGACTG-TTTTCCTTAATGGCATCTACTGTA-CTTAAAGCAAG----GAACTCATCTTTGA
Pseudonaja-textilis-N_EBS10Xv2-PRI_NW020769337.1-c8326428-8326231_NONE_303           ACAGGCTGT-----GGCTTAGTGACATATTGACTG-TTTTCCTTAATGGCATCTACTGTA-CTTAAAGCAAG----GAACTCATCTTTGA
Notechis-scutatus_TS10Xv2-PRI_ULFQ01000001.1-43599067-43599263_ENSNSUG00000002523_301 ACAGGCTGT-----GGCTTAGTGACATATTGACTG-TTTTCCTTAATGGCATCTACTGTA-CTTAAAGCAAG----GAACTCATCTTTGA
Chelonoidis-abingdonii_ASM359739v1_PKMU01001678.1-c2481550-2481351_ENSCABG00000009144_297 ACAGGCTGA-----GGCTTAGTGACATATTGACTA-TTTTCCTTAATGGCATCTACTGTA-CTTAATGCAAG----GAACTCATCTTTGA
Alligator-mississippiensis-N_ASM28112v4_NW017711041.1-26335021-26335220_NONE_328     ACAGGCCAT-----GGCTTAGTGACATATTGACTG-TTTTCCTTAATGGCATCTACTGTA-CTTAATGCAAG----GAACTCATCTTTGA
Nothoprocta-perdicaria_notPer1_PTEW01000005.1-3871716-3871914_ENSNPEG00000002221_299 ACAGGCCGT-----GGCTTAGTGACATATTGACTG-TTTTCCTTAATGGCATCTACTGTA-CTTAATGCAAA----GAACTCATCTTCGG
Gallus-gallus_GRCg6a_9-c20276997-20276798_MECOM_310                                  ACAGGCCGT-----GGCTTAGTGACATATTGACTG-TTTTCCTTAATGGCATCTACTGTA-CTTAATGCAGA----GAACTCATCTTTGA
Calidris-pugnax_ASM143184v1_KQ482134.1-1573146-1573344_MECOM_308                     ACAGGCCAT-----GGCTTAGTGACATATTGACTG-TTTTCCTTAATGGCATCTACTGTA-CTTAATGCAAA----GAACTCATCTTTGG
Ornithorhynchus-anatinus_OANA5_Contig328-c389526-389326_ENSOANG00000007584_323       ACAGGCCAC-----GGCTTAGTGACATATTGACTG-TTTTCCTTAATGGCATCTACTGTA-TTTAATGCAAA----GATGTCATCTTTGA
Monodelphis-domestica_monDom5_7-c233068836-233068636_ENSMODG00000024658_291          ACAGGCCGG-----GCCTCCGTGACATATTGACTG-GTTTCCTTAATGGCACCTACCGTA-TTTAATGCAAG----GATGTCATCTCGAG
Choloepus-hoffmanni_choHof1_scaffold1067-25174-25374_ENSCHOG00000003815_345          ACAGGCCGT-----GGCTTAGTGACATATTGACTG-TTTTCCTTAATGGCATCTACCGTA-TTTAATGCAAG----GATGTCATCTTTGA
Chrysochloris-asiatica-N_ChrAsi1-0_NW006408561.1-16774181-16774381_NONE_345          ACAGGTCGT-----GGCTTAGTGACATATTGACTG-TTTTCCTTAATGGCATCTACCGTA-TTTAATGCAAG----GATGTCATCTTTGA
Condylura-cristata-N_ConCri1-0_NW004567104.1-7625208-7625408_NONE_350                ACAGGGCAT-----GGCTTGGTGACATATTGACTG-TTTTCCTTAATGGCATCTACCGTA-TTTAATGCAAG----GATGTCATCTTTGA
Myotis-lucifugus_0_GL429820-c3017858-3017658_ENSMLUG00000015759_354                  ACAGGCCAT-----GGCTTGGTGACATATTGACTG-TTTTCCTTAATGGCATCTACCGTA-TTTAATGCAAG----GATGTCATCTTTGA
Equus-caballus_0_19-12246137-12246337_MECOM_350                                      ACAGGCCGT-----GGCTTGGTGACATATTGACTG-TTTTCCTTAATGGCATCTACCGTA-TTTAATGCAAG----GATGTCATCTTTGA
Bos-taurus_2_1-c98035718-98035518_ENSBTAG00000047144_345                             ACAGGCTGT-----GGCTTGGTGACATATTGACTG-TTTTCCTTAATGGCATCTACTGTA-TTTAATGCAAG----GATGTCATCTTTGA
Heterocephalus-glaber-male_0_JH173818.1-448008-448186_ENSHGLG00100021283_319         ACAGGCCAT-----GGCTTAGTGACATATTGACTG-TTTTCCTTAATGGCATCTACCGTA-TTTAATGCAAG----GATGTCATCTTTGA
Nannospalax-galili_0_KL200152.1-c2486863-2486663_ENSNGAG00000001009_345              ACAGGCCGT-----GGCTTAGTGACATATTGACTG-TTTTCCTTAATGGCATCTACCGTA-TTTAATGCAAG----GATGTCATCTTTGA
Mus-musculus_GRCm38_3-30229286-30229486_Mecom_350                                    ACAGGCCGT-----GGCTTAGTGACATATTGACTG-TTTTCCTTAATGGCATCTACCGTA-TTTAATGCAAG----GATGTCATCTTTGA
Propithecus-coquereli_0_KQ018509.1-3029968-3030168_MECOM_350                         ACAGGCCAT-----GGCTTAGTGACATATTGACTG-TTTTCCTTAATGGCATCTACCATA-TTTAATTCAAG----GATGTCATCTTTGA
Aotus-nancymaae_0_KZ197721.1-c792585-792385_MECOM_350                                ACAGGCCAT-----GGCTTAGTGACATATTGACTG-TTTTCCTTAATGGCATCTACTGTA-TTTAATGCAAG----GATGTCCTCTTTGA
Homo-sapiens_GRCh38_3-169364745-169364945_MECOM_359                                  ACAGGCCAT-----GGCTTGGTGACATATTGACTG-TTTTCCTTAATGGCATCTACCGTA-TTTAATGCAAG----GATGTCATCTTTGA
YOURSEQ1_Homo-sapiens_GRCh38_3-169364745-169364945                                   ACAGGCCAT-----GGCTTaGTGACATATTGACTG-TTTTCCTTAATGGCATCTACCGTA-TTTAATGCAAG----GATGTCATCTTTGA

                                                                                     181
Used4treeSearch                                                                      110000000011111001111111111111111111110
```

Fig. S4- 4

Callorhinchus-milii_3_KI635855.1-c6442281-6442076_ENSCMIG00000008884_190
Rhincodon-typus-N_v2_NW018043067.1-26837-27027_NONE_182
Erpetoichthys-calabaricus_1_2-138640288-138640492_ENSECRG00000013444_203
Lepisosteus-oculatus_LepOcu1_LG14-c19988744-19988541_ENSLOCG00000009550_198
Danio-rerio_GRCz11_15-35542490-35542588_mecom_108
Gasterosteus-aculeatus_BROADS1_groupI-c5410722-5410623_ENSGACG00000007274_97
Tetraodon-nigroviridis_TETRAODON8_Unrandom-c54161107-54161030_ENSTNIG00000002725_58
Oryzias-latipes_ASM223467v1_13-23170268-23170388_mecom_104
Oryzias-latipes_ASM223467v1_13-23170479-23170561_mecom_67
Latimeria-chalumnae_LatCha1_JH126697.1-c1747311-1747112_ENSLACG00000016795_265
Microcaecilia-unicolor-N_aMicUni1-1_NC044040.1-195525342-195525529_NONE_270
Xenopus-tropicalis_v91_KV460428.1-c193815-193644_erp29_120
Sphenodon-punctatus_ASM311381v1_QEPC01014730.1-c6120370-6120172_MECOM_320
Podarcis-muralis-N_PodMur10_NC041316.1-84176510-84176708_NONE_295
Anolis-carolinensis_0_3-c8300518-8300320_ENSACAG00000015690_286
Python-bivittatus-N_502_NW006534654.1-166579-166775_NONE_299
Protobothrops-mucrosquamatus-N_1-0_NW015387498.1-c242829-242632_NONE_298
Thamnophis-sirtalis-N_6-0_NW013657765.1-c228880-228683_NONE_298
Pseudonaja-textilis-N_EBS10Xv2-PRI_NW020769337.1-c8326428-8326231_NONE_303
Notechis-scutatus_TS10Xv2-PRI_ULFQ01000001.1-43599067-43599263_ENSNSUG00000002523_301
Chelonoidis-abingdonii_ASM359739v1_PKMU01001678.1-c2481550-2481351_ENSCABG00000009144_297
Alligator-mississippiensis-N_ASM28112v4_NW017711041.1-26335021-26335220_NONE_328
Nothoprocta-perdicaria_notPer1_PTEW01000005.1-3871716-3871914_ENSNPEG00000002221_299
Gallus-gallus_GRCg6a_9-c20276997-20276798_MECOM_310
Calidris-pugnax_ASM143184v1_KQ482134.1-1573146-1573344_MECOM_308
Ornithorhynchus-anatinus_OANA5_Contig328-c389526-389326_ENSOANG00000007584_323
Monodelphis-domestica_monDom5_7-c233068836-233068636_ENSMODG00000024658_291
Choloepus-hoffmanni_choHof1_scaffold1067-25174-25374_ENSCHOG00000003815_345
Chrysochloris-asiatica-N_ChrAsi1-0_NW006408561.1-16774181-16774381_NONE_345
Condylura-cristata-N_ConCri1-0_NW004567104.1-7625208-7625408_NONE_350
Myotis-lucifugus_0_GL429820-c3017858-3017658_ENSMLUG00000015759_354
Equus-caballus_0_19-12246137-12246337_MECOM_350
Bos-taurus_2_1-c98035718-98035518_ENSBTAG00000047144_345
Heterocephalus-glaber-male_0_JH173818.1-448008-448186_ENSHGLG00100021283_319
Nannospalax-galili_0_KL200152.1-c2486863-2486663_ENSNGAG00000001009_345
Mus-musculus_GRCm38_3-30229286-30229486_Mecom_350
Propithecus-coquereli_0_KQ018509.1-3029968-3030168_MECOM_350
Aotus-nancymaae_0_KZ197721.1-c792585-792385_MECOM_350
Homo-sapiens_GRCh38_3-169364745-169364945_MECOM_359
YOURSEQ1_Homo-sapiens_GRCh38_3-169364745-169364945

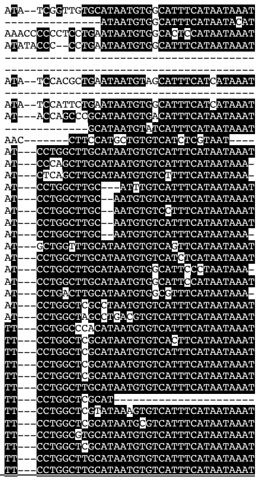

CNS tree:

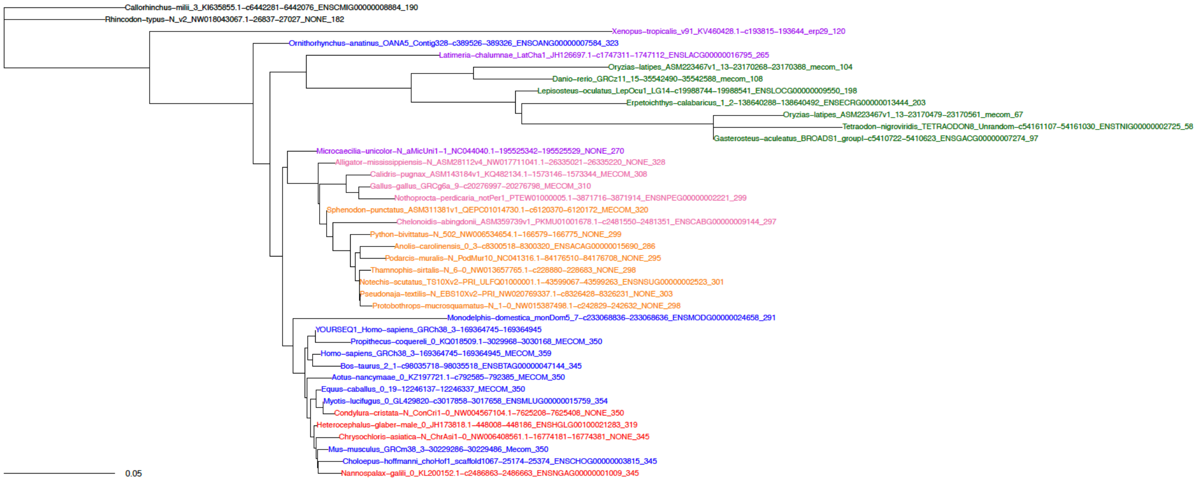

############### Settings ###############

Taxon sampling:
Callorhinchus-milii_Black
Rhincodon-typus-N_Black
Erpetoichthys-calabaricus_Green
Lepisosteus-oculatus_Green
Danio-rerio_Green
Gasterosteus-aculeatus_Green
Tetraodon-nigroviridis_Green
Oryzias-latipes_Green
Latimeria-chalumnae_Purple
Microcaecilia-unicolor-N_Purple
Xenopus-tropicalis_Purple
Sphenodon-punctatus_Orange
Podarcis-muralis-N_Orange
Anolis-carolinensis_Orange
Python-bivittatus-N_Orange
Protobothrops-mucrosquamatus-N_Orange
Thamnophis-sirtalis-N_Orange
Pseudonaja-textilis-N_Orange
Notechis-scutatus_Orange
Chelonoidis-abingdonii_Magenta
Alligator-mississippiensis-N_Magenta
Nothoprocta-perdicaria_Magenta
Gallus-gallus_Magenta
Calidris-pugnax_Magenta
Ornithorhynchus-anatinus_Blue
Monodelphis-domestica_Blue
Choloepus-hoffmanni_Blue
Chrysochloris-asiatica-N_Red
Condylura-cristata-N_Red
Myotis-lucifugus_Blue
Equus-caballus_Blue
Bos-taurus_Blue
Heterocephalus-glaber-male_Red
Nannospalax-galili_Red
Mus-musculus_Blue
Propithecus-coquereli_Blue
Aotus-nancymaae_Blue
Homo-sapiens_Blue

>blastn_task
blastn

>blastn_word_size
11

>blastn_E-value_threshold_for_reported_sequences
1e-3

>Number_of_hits_to_report_per_genome (num_alignments)
2

>Percent_identity_cutoff(perc_identity)
0

>Percent_identity_cutoff(template_length)
18

Fig. S4- 5

```
Color template_length:
18

>TreeSearchMethod
Neighbor-joining method (Saitou and Nei 1986)

>SubstitutionModel
TN93 (Tamura and Nei 1993) + gamma

>Dependencies
BLAST 2.7.1+
MAFFT v7.356b
trimAl 1.2rev59
ape in R, Version: 5.0

Analysis time: 34.0 seconds

Database:
Callorhinchus-milii              :Callorhinchus_milii.Callorhinchus_milii-6.1.3.dna_rm.toplevel.fa
Rhincodon-typus-N                :Rhincodon-typus-N_GCF_001642345.1_ASM164234.v2.dna_nb.genomic.fna
Erpetoichthys-calabaricus        :Erpetoichthys_calabaricus.fErpCal1.1.dna_rm.toplevel.fa
Lepisosteus-oculatus             :Lepisosteus_oculatus.LepOcu1.dna_rm.toplevel.fa
Danio-rerio                      :Danio_rerio.GRCz11.dna_rm.primary_assembly.fa
Gasterosteus-aculeatus           :Gasterosteus_aculeatus.BROADS1.dna_rm.toplevel.fa
Tetraodon-nigroviridis           :Tetraodon_nigroviridis.TETRAODON8.dna_rm.toplevel.fa
Oryzias-latipes                  :Oryzias_latipes.ASM223467v1.dna_rm.toplevel.fa
Latimeria-chalumnae              :Latimeria_chalumnae.LatCha1.dna_rm.toplevel.fa
Microcaecilia-unicolor-N         :Microcaecilia-unicolor-N.GCF_901765095-1.aMicUni1-1.dna_nb.genomic.fna
Xenopus-tropicalis               :Xenopus-tropicalis.v91.dna_rm.toplevel.fa
Sphenodon-punctatus              :Sphenodon_punctatus.ASM311381v1.dna_rm.toplevel.fa
Podarcis-muralis-N               :Podarcis-muralis-N.GCF_004329235-1.PodMur10.dna_nb.genomic.fna
Anolis-carolinensis              :Anolis_carolinensis.AnoCar2.0.dna_rm.toplevel.fa
Python-bivittatus-N              :Python-molurus-bivittatus-N.GCF_000186305-1_.502.dna_nb.genomic.fna
Protobothrops-mucrosquamatus-N   :Protobothrops-mucrosquamatus-N.GCF_001527695-2.1-0.dna_nb.genomic.fna
Thamnophis-sirtalis-N            :Thamnophis-sirtalis-N.GCF_001077635-1.6-0.dna_nb.genomic.fna
Pseudonaja-textilis-N            :Pseudonaja-textilis-N.GCF_900518735-1.EBS10Xv2-PRI.dna_nb.genomic.fna
Notechis-scutatus                :Notechis_scutatus.TS10Xv2-PRI.dna_rm.toplevel.fa
Chelonoidis-abingdonii           :Chelonoidis_abingdonii.ASM359739v1.dna_rm.toplevel.fa
Alligator-mississippiensis-N     :Alligator-mississippiensis-N.GCF_000281125-3.ASM28112v4.dna_nb.genomic.fna
Nothoprocta-perdicaria           :Nothoprocta_perdicaria.notPer1.dna_rm.toplevel.fa
Gallus-gallus                    :Gallus_gallus.GRCg6a.dna_rm.toplevel.fa
Calidris-pugnax                  :Calidris_pugnax.ASM143184v1.dna_rm.toplevel.fa
Ornithorhynchus-anatinus         :Ornithorhynchus_anatinus.OANA5.dna_rm.toplevel.fa
Monodelphis-domestica            :Monodelphis_domestica.monDom5.dna_rm.toplevel.fa
Choloepus-hoffmanni              :Choloepus_hoffmanni.choHof1.dna_rm.toplevel.fa
Chrysochloris-asiatica-N         :Chrysochloris-asiatica-N.GCF_000296735-1.ChrAsi1-0.dna_nb.genomic.fna
Condylura-cristata-N             :Condylura-cristata-N.GCF_000260355-1.ConCri1-0.dna_nb.genomic.fna
Myotis-lucifugus                 :Myotis_lucifugus.Myoluc2.0.dna_rm.toplevel.fa
Equus-caballus                   :Equus_caballus.EquCab3.0.dna_rm.toplevel.fa
Bos-taurus                       :Bos_taurus.ARS-UCD1.2.dna_rm.toplevel.fa
Heterocephalus-glaber-male       :Heterocephalus_glaber_male.HetGla_1.0.dna_rm.toplevel.fa
Nannospalax-galili               :Nannospalax_galili.S.galili_v1.0.dna_rm.toplevel.fa
Mus-musculus                     :Mus_musculus.GRCm38.dna_rm.primary_assembly.fa
Propithecus-coquereli            :Propithecus_coquereli.Pcoq_1.0.dna_rm.toplevel.fa
Aotus-nancymaae                  :Aotus_nancymaae.Anan_2.0.dna_rm.toplevel.fa
Homo-sapiens                     :Homo_sapiens.GRCh38.dna_rm.primary_assembly.fa
```

Fig. S4- 6

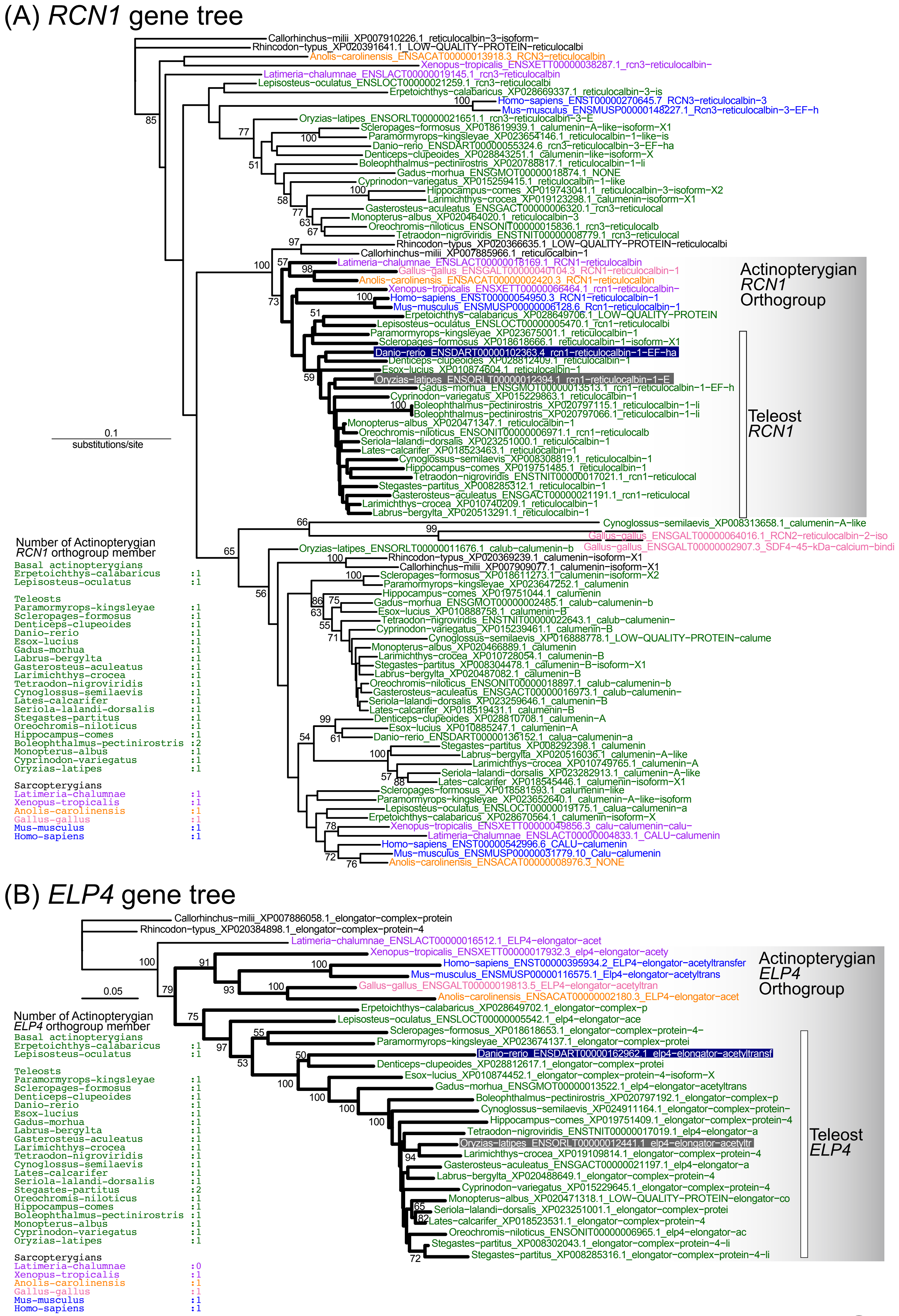
(A) RCN1 gene tree
Callorhinchus-milii_XP007910226.1_reticulocalbin-3-isoform-
Rhincodon-typus_XP020391641.1_LOW-QUALITY-PROTEIN-reticulocalbi
Anolis-carolinensis_ENSACAT00000013918.3_RCN3-reticulocalbin
Xenopus-tropicalis_ENSXETT00000038287.1_rcn3-reticulocalbin-
Latimeria-chalumnae_ENSLACT00000019145.1_rcn3-reticulocalbin
Lepisosteus-oculatus_ENSLOCT00000021259.1_rcn3-reticulocalbi
Erpetoichthys-calabaricus_XP028669337.1_reticulocalbin-3-is
Homo-sapiens_ENST00000270645.7_RCN3-reticulocalbin-3
Mus-musculus_ENSMUSP00000148227.1_Rcn3-reticulocalbin-3-EF-h
Oryzias-latipes_ENSORLT00000021651.1_rcn3-reticulocalbin-3-E
Scleropages-formosus_XP018619939.1_calumenin-A-like-isoform-X1
Paramormyrops-kingsleyae_XP023654146.1_reticulocalbin-1-like-is
Danio-rerio_ENSDART00000055324.6_rcn3-reticulocalbin-3-EF-ha
Denticeps-clupeoides_XP028843251.1_calumenin-like-isoform-X
Boleophthalmus-pectinirostris_XP020788817.1_reticulocalbin-1-li
Gadus-morhua_ENSGMOT00000018874.1_NONE
Cyprinodon-variegatus_XP015259415.1_reticulocalbin-1-like
Hippocampus-comes_XP019743041.1_reticulocalbin-3-isoform-X2
Larimichthys-crocea_XP019123298.1_calumenin-isoform-X1
Gasterosteus-aculeatus_ENSGACT00000006320.1_rcn3-reticulocal
Monopterus-albus_XP020464020.1_reticulocalbin-3
Oreochromis-niloticus_ENSONIT00000015836.1_rcn3-reticulocalb
Tetraodon-nigroviridis_ENSTNIT00000008779.1_rcn3-reticulocal
Rhincodon-typus_XP020366635.1_LOW-QUALITY-PROTEIN-reticulocalbi
Callorhinchus-milii_XP007885966.1_reticulocalbin-1
Latimeria-chalumnae_ENSLACT00000018169.1_RCN1-reticulocalbin
Gallus-gallus_ENSGALT00000040104.3_RCN1-reticulocalbin-1
Anolis-carolinensis_ENSACAT00000002420.3_RCN1-reticulocalbin
Xenopus-tropicalis_ENSXETT00000066464.1_rcn1-reticulocalbin-
Homo-sapiens_ENST00000054950.3_RCN1-reticulocalbin-1
Mus-musculus_ENSMUSP00000006128.6_Rcn1-reticulocalbin-1
Erpetoichthys-calabaricus_XP028649706.1_LOW-QUALITY-PROTEIN
Lepisosteus-oculatus_ENSLOCT00000005470.1_rcn1-reticulocalbi
Paramormyrops-kingsleyae_XP023675001.1_reticulocalbin-1
Scleropages-formosus_XP018618666.1_reticulocalbin-1-isoform-X1
Danio-rerio_ENSDART00000102363.4_rcn1-reticulocalbin-1-EF-ha
Denticeps-clupeoides_XP028812409.1_reticulocalbin-1
Esox-lucius_XP010874604.1_reticulocalbin-1
Oryzias-latipes_ENSORLT00000012394.1_rcn1-reticulocalbin-1-E
Gadus-morhua_ENSGMOT00000013513.1_rcn1-reticulocalbin-1-EF-h
Cyprinodon-variegatus_XP015229863.1_reticulocalbin-1
Boleophthalmus-pectinirostris_XP020797115.1_reticulocalbin-1-li
Boleophthalmus-pectinirostris_XP020797066.1_reticulocalbin-1-li
Monopterus-albus_XP020471347.1_reticulocalbin-1
Oreochromis-niloticus_ENSONIT00000006971.1_rcn1-reticulocalb
Seriola-lalandi-dorsalis_XP023251000.1_reticulocalbin-1
Lates-calcarifer_XP018523463.1_reticulocalbin-1
Cynoglossus-semilaevis_XP008308819.1_reticulocalbin-1
Hippocampus-comes_XP019751485.1_reticulocalbin-1
Tetraodon-nigroviridis_ENSTNIT00000017021.1_rcn1-reticulocal
Stegastes-partitus_XP008285312.1_reticulocalbin-1
Gasterosteus-aculeatus_ENSGACT00000021191.1_rcn1-reticulocal
Larimichthys-crocea_XP010740209.1_reticulocalbin-1
Labrus-bergylta_XP020513291.1_reticulocalbin-1
Actinopterygian RCN1 Orthogroup
Teleost RCN1
0.1 substitutions/site
Cynoglossus-semilaevis_XP008313658.1_calumenin-A-like
Gallus-gallus_ENSGALT00000064016.1_RCN2-reticulocalbin-2-iso
Gallus-gallus_ENSGALT00000002907.3_SDF4-45-kDa-calcium-bindi
Oryzias-latipes_ENSORLT00000011676.1_calub-calumenin-b
Rhincodon-typus_XP020369239.1_calumenin-isoform-X1
Callorhinchus-milii_XP007909077.1_calumenin-isoform-X1
Scleropages-formosus_XP018611273.1_calumenin-isoform-X2
Paramormyrops-kingsleyae_XP023647252.1_calumenin
Hippocampus-comes_XP019751044.1_calumenin
Gadus-morhua_ENSGMOT00000002485.1_calub-calumenin-b
Esox-lucius_XP010888758.1_calumenin-B
Tetraodon-nigroviridis_ENSTNIT00000022643.1_calub-calumenin-
Cyprinodon-variegatus_XP015239461.1_calumenin-B
Cynoglossus-semilaevis_XP016888778.1_LOW-QUALITY-PROTEIN-calume
Monopterus-albus_XP020466889.1_calumenin
Larimichthys-crocea_XP010728054.1_calumenin-B
Stegastes-partitus_XP008304478.1_calumenin-B-isoform-X1
Labrus-bergylta_XP020487082.1_calumenin-B
Oreochromis-niloticus_ENSONIT00000018897.1_calub-calumenin-b
Gasterosteus-aculeatus_ENSGACT00000016973.1_calub-calumenin-
Seriola-lalandi-dorsalis_XP023259646.1_calumenin-B
Lates-calcarifer_XP018519431.1_calumenin-B
Denticeps-clupeoides_XP028810708.1_calumenin-A
Esox-lucius_XP010885247.1_calumenin-A
Danio-rerio_ENSDART00000136152.1_calua-calumenin-a
Stegastes-partitus_XP008292398.1_calumenin
Labrus-bergylta_XP020516036.1_calumenin-A-like
Larimichthys-crocea_XP010749765.1_calumenin-A
Seriola-lalandi-dorsalis_XP023282913.1_calumenin-A-like
Lates-calcarifer_XP018545446.1_calumenin-isoform-X1
Scleropages-formosus_XP018581593.1_calumenin-like
Paramormyrops-kingsleyae_XP023652640.1_calumenin-A-like-isoform
Lepisosteus-oculatus_ENSLOCT00000019175.1_calua-calumenin-a
Erpetoichthys-calabaricus_XP028670564.1_calumenin-isoform-X
Xenopus-tropicalis_ENSXETT00000049856.3_calu-calumenin-calu-
Latimeria-chalumnae_ENSLACT00000004833.1_CALU-calumenin
Homo-sapiens_ENST00000542996.6_CALU-calumenin
Mus-musculus_ENSMUSP00000031779.10_Calu-calumenin
Anolis-carolinensis_ENSACAT00000008976.3_NONE
Number of Actinopterygian RCN1 orthogroup member
Basal actinopterygians
Erpetoichthys-calabaricus :1
Lepisosteus-oculatus :1
Teleosts
Paramormyrops-kingsleyae :1
Scleropages-formosus :1
Denticeps-clupeoides :1
Danio-rerio :1
Esox-lucius :1
Gadus-morhua :1
Labrus-bergylta :1
Gasterosteus-aculeatus :1
Larimichthys-crocea :1
Tetraodon-nigroviridis :1
Cynoglossus-semilaevis :1
Lates-calcarifer :1
Seriola-lalandi-dorsalis :1
Stegastes-partitus :1
Oreochromis-niloticus :1
Hippocampus-comes :1
Boleophthalmus-pectinirostris :2
Monopterus-albus :1
Cyprinodon-variegatus :1
Oryzias-latipes :1
Sarcopterygians
Latimeria-chalumnae :1
Xenopus-tropicalis :1
Anolis-carolinensis :1
Gallus-gallus :1
Mus-musculus :1
Homo-sapiens :1
(B) ELP4 gene tree
Callorhinchus-milii_XP007886058.1_elongator-complex-protein
Rhincodon-typus_XP020384898.1_elongator-complex-protein-4
Latimeria-chalumnae_ENSLACT00000016512.1_ELP4-elongator-acet
Xenopus-tropicalis_ENSXETT00000017932.3_elp4-elongator-acety
Homo-sapiens_ENST00000395934.2_ELP4-elongator-acetyltransfer
Mus-musculus_ENSMUSP00000116575.1_Elp4-elongator-acetyltrans
Gallus-gallus_ENSGALT00000019813.5_ELP4-elongator-acetyltran
Anolis-carolinensis_ENSACAT00000002180.3_ELP4-elongator-acet
Erpetoichthys-calabaricus_XP028649702.1_elongator-complex-p
Lepisosteus-oculatus_ENSLOCT00000005542.1_elp4-elongator-ace
Scleropages-formosus_XP018618653.1_elongator-complex-protein-4-
Paramormyrops-kingsleyae_XP023674137.1_elongator-complex-protei
Danio-rerio_ENSDART00000162962.1_elp4-elongator-acetyltransf
Denticeps-clupeoides_XP028812617.1_elongator-complex-protei
Esox-lucius_XP010874452.1_elongator-complex-protein-4-isoform-X
Gadus-morhua_ENSGMOT00000013522.1_elp4-elongator-acetyltrans
Boleophthalmus-pectinirostris_XP020797192.1_elongator-complex-p
Cynoglossus-semilaevis_XP024911164.1_elongator-complex-protein-
Hippocampus-comes_XP019751409.1_elongator-complex-protein-4
Tetraodon-nigroviridis_ENSTNIT00000017019.1_elp4-elongator-a
Oryzias-latipes_ENSORLT00000012441.1_elp4-elongator-acetyltr
Larimichthys-crocea_XP019109814.1_elongator-complex-protein-4
Gasterosteus-aculeatus_ENSGACT00000021197.1_elp4-elongator-a
Labrus-bergylta_XP020488649.1_elongator-complex-protein-4
Cyprinodon-variegatus_XP015229645.1_elongator-complex-protein-4
Monopterus-albus_XP020471318.1_LOW-QUALITY-PROTEIN-elongator-co
Seriola-lalandi-dorsalis_XP023251001.1_elongator-complex-protei
Lates-calcarifer_XP018523531.1_elongator-complex-protein-4
Oreochromis-niloticus_ENSONIT00000006965.1_elp4-elongator-ac
Stegastes-partitus_XP008302043.1_elongator-complex-protein-4-li
Stegastes-partitus_XP008285316.1_elongator-complex-protein-4-li
Actinopterygian ELP4 Orthogroup
Teleost ELP4
0.05
Number of Actinopterygian ELP4 orthogroup member
Basal actinopterygians
Erpetoichthys-calabaricus :1
Lepisosteus-oculatus :1
Teleosts
Paramormyrops-kingsleyae :1
Scleropages-formosus :1
Denticeps-clupeoides :1
Danio-rerio :1
Esox-lucius :1
Gadus-morhua :1
Labrus-bergylta :1
Gasterosteus-aculeatus :1
Larimichthys-crocea :1
Tetraodon-nigroviridis :1
Cynoglossus-semilaevis :1
Lates-calcarifer :1
Seriola-lalandi-dorsalis :1
Stegastes-partitus :2
Oreochromis-niloticus :1
Hippocampus-comes :1
Boleophthalmus-pectinirostris :1
Monopterus-albus :1
Cyprinodon-variegatus :1
Oryzias-latipes :1
Sarcopterygians
Latimeria-chalumnae :0
Xenopus-tropicalis :1
Anolis-carolinensis :1
Gallus-gallus :1
Mus-musculus :1
Homo-sapiens :1

Fig. S5

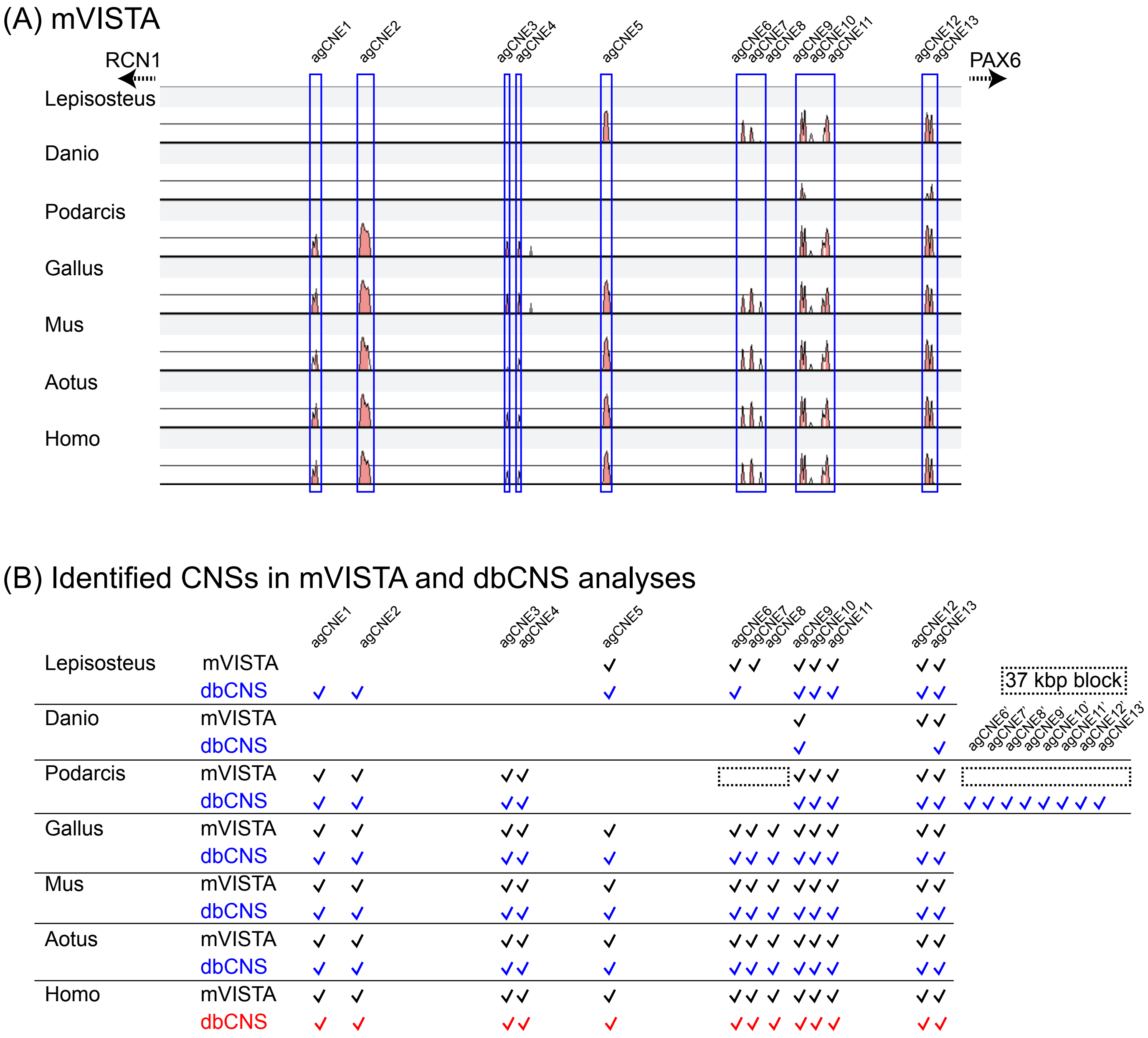
(A) mVISTA
RCN1
PAX6
agCNE1
agCNE2
agCNE3
agCNE4
agCNE5
agCNE6
agCNE7
agCNE8
agCNE9
agCNE10
agCNE11
agCNE12
agCNE13
Lepisosteus
Danio
Podarcis
Gallus
Mus
Aotus
Homo
(B) Identified CNSs in mVISTA and dbCNS analyses
mVISTA
dbCNS
37 kbp block
agCNE6'
agCNE7'
agCNE8'
agCNE9'
agCNE10'
agCNE11'
agCNE12'
agCNE13'

Fig. S6

Supplementary Table S1. Human CNS coordinates (hg19) used in the gnathostome analysis

Name	Coordinate	Reference
E-250	11:32085525-32086304	Bhatia et al. (2014)
agCNE1	11:32062882-32063230	Bhatia et al. (2014)
agCNE2	11:32052624-32053243	Bhatia et al. (2014)
agCNE3	11:32024810-32024977	Bhatia et al. (2014)
agCNE4	11:32016455-32017112	Bhatia et al. (2014)
agCNE5	11:31989455-31989799	Bhatia et al. (2014)
agCNE6	11:31898728-31898965	Bhatia et al. (2014)
agCNE7	11:31897563-31898031	Bhatia et al. (2014)
agCNE8	11:31896241-31896769	Bhatia et al. (2014)
agCNE9	11:31847850-31848450	Bhatia et al. (2014)
agCNE10	11:31846835-31847159	Bhatia et al. (2014)
agCNE11	11:31845331-31846404	Bhatia et al. (2014)
agCNE12	11:31843629-31844067	Bhatia et al. (2014)
cre86	11:31843671-31844100	Partha et al. (2017)
agCNE13	11:31843215-31843530	Bhatia et al. (2014)
P2	11:31841310-31841722	Bhatia et al. (2014)
P0	11:31839356-31839510	Bhatia et al. (2014)
cre149	11:31825492-31826050	Partha et al. (2017)
E+120	11:31712679-31713314	Bhatia et al. (2014)
cre21	11:31676830-31677382	Partha et al. (2017)

Supplementary Table S2 Query sequences

(A) Gnathostome analysis

>E-250_Homo-sapiens-hg19_GRCh37_11-32085525-32086304
TAACTATCTCCTAAGATTCCCACACACACATTTTAAGAGAGCAATTATTCCCTCTGAAGGCAACACTTGTTATTCTGAAGCACTGAAG
CGAAGCCGCCAGTACACAGAAGCCACTTGGATGTTGCTTAGCAGATGTTTACAGATAGCTTGATGTTAATTTCTACACAAAAGATATA
TTTCTGGCCTTGCCTTATTGTTTATGATTCACGACTAAATTCTGGGAACCTTGAGTCTGGCCCATCATCCTGCCTGAGCTGACCATAT
TTGACACAGACAGTTTCCATTCATGGTGGCCACATGCCCTGTCTACACCATCCACACCCCAAGAGGTAATTCAGTGCTTGTGTTTTAA
CATAAACTTCAAGTCACATGCATACTAACACTACTTCATGAGCAAGAAATTACCACGTCTGGTTCATTGACATTTACTTCTGGGAACC
ATTTCAATCAGATTAATTCATTTATCTAAATAATCTTCCACTCACTTCACAACATCAAAACTCACCAATACATTTTCTGTTGATGCTT
CCAACTTTCCTTTTTGTACAGTGAGAAGCAAGATTAATTTTTACCTGGAGTTCACAGACTGGGCACT

>agCNE1_Homo-sapiens-hg19_GRCh37_11-32062882-32063230
TGTCATTGCCACCATTGTCTCTTGTCACAATCCTAATTCTATGTTCTGATAATAAAACTTCATTAGTGTCTGCACCTCCTTTCCCGTA
CCTTTGAAACACTGTCTTCTGCTGAAACGATTAGCAGTGCGCTGCGTCAGATCATCCGGCTGTCTGACAGCCATTGTGAGTGAGCTAA
ATTGTGAGAAAATGAAAATTTCTGACAATTTCCATTATTGTCAAATGGAGATATTTACTTGTACAAATATTAAAGGTTATTTAAAGAT
GTAAACGGACTTCATTTCATTAGTGGTATGCATCAGGTAATAATTTAAAACAACTACCCAGCCTCATTCAAATAAATTACCAATT

>agCNE2_Homo-sapiens-hg19_GRCh37_11-32052624-32053243
GAATAAATTATAGATGAAACCCCCATCATATGTTTATAAATACACTAACATTAGCATTTTACTTCCTCATAAATCTGCAGCAAATACT
CAGGCACAAGATAGAAAGCCCTGTAAAACAATGATATATTCTCTGCTTGCAGGCATTGACTAGTGGCCCATTCACTCCCGGTGCTGTT
CTTCAACCACGCTCCCTCCGCTCATGCCCCGTCATTTAAAATCCATTTTAATTTAAAGATGCACAAGGCAGTGCTCGTGGTACATTTA
CTCATCCTAGTTATACATCATGGAGCAAGGAAAAGAAAATTGTTTCATCTAGAGATACGGTTCAATGTGGTCACCACTGAACAGTGGA
GTAATTACCATATCAGACCCACTCAAAATGCACTCAGGGTGTTCTGTGACCTATCATTTGCTAATCTGCATAGTGAAGTATAGCTTCA
TAACTATAAATTGCTGACTTAATTAAAACCAAGACCTTTCTGTCATTTAATTACAGGGAGAAAGGTTATTGCTTAAATGCAATTTTCC
AAATGTGATTTCTTAACAGTTATTAACTGCTTCACCATCATTTATTGTTTCAGATTTTTCAATTTGAAAATACTTCCCGTGACAGAAG
TAAT

>agCNE3_Homo-sapiens-hg19_GRCh37_11-32024810-32024977
TTACCCTGTGAAAGCCCCATCACTCACAGAAACTATTGCTGAGGAGGAGAGGATGATTTTATTTAGCTAATTTTTAACAAATCTCCAA
TGTTGCCCACATCTCCTGAAGCTGGGAGGAGGGATAATCAAGTCAAGCCCCATTGACTAGGAGAATAGTGAACTGTGGAA

>agCNE4_Homo-sapiens-hg19_GRCh37_11-32016455-32017112
TTTCCCTCTTCCAGCACAGAAATTTTGAGGAAAGACATGAAAAGCCCAGGAAAAATGGAAAGCGTTTAGATGCACATTTTATTAGCAG
GTTCNNNNNNNNNNNNNNNNNNNNNNNNNNNNNNNNNNGTTAAAAAGCAAGAGGCAAAAGAATTTGGCACCATTTCCATCATGTTGAT
TCTTCTGGGTTTCTCAAATGTTGTGATCCCTGGAAAGCCGGGATTCATGCTTACCTCCACTCTTCATTAAGGCGGCCGTGGCTGTGAG
CTGGGGAAGACAGGCTGCTGGTGCGGTGGGTGGGCGTGCTGAATAACACCCGAACCACAGCTGTGGCTGCCCTTGTCAGAGTGCCAGC
CTGCCTAACCCAGTCTGTTCTTGCCTGCTACCTGGCCCTCGCCCATCTGCTGCAGAATTCATAAAGTGCTGAAGATCACAAACATCTG
AACCTATTATAGGTAACATACTTCAATTACATTCTGATTGTGGGCTTGGCTTCCCCCAAGCCTGGTTTGTAATGGTGCACAGATCAAA
TTCTCCCCACAAAAGAAGCACTCATCTCTAGGTAATCTCATCATGTTGGGTGTGCTTAAGAAAGAAGCCATTCTCCTAGATGCTGCTT
TGTGTTATTCAGTTACCTGGCAAAGGTTCCCCCAGATGTAAA

>agCNE5-Id855_Homo-sapiens-hg19_GRCh37_11-31989455-31989799
TGCTACCTGATAGCTACTAATTGCTGCAACTACATTTCTGAATTGCAAATTTTCTGCACTGATAAAAACTTTGATTGCCTGTCAGCTA
CACATAAGGAAAAGATCAGCAGTGCTAAATGAGGGGTTCCACGCTTGGATAATAATGCCTAATGAATTATTCAGGAAAGAATGCTTGA
ATGAAGCAAACAATTTGAAGGGTTACTAGCAGCCGTAACAGTTCAACAGGCAAAAGAACAAAAGTATCATCCTGAACATTACACTAAT
GATTAAAAAATGTGTATTAATTCTAGATTTGGAGAAAAAAGAAATGCCAATCTGATTTTAAAGGTTAATCATTATTTTCTC

>agCNE6_Homo-sapiens-hg19_GRCh37_11-31898728-31898965
CCCCGCTCCAGGGCTATACATATGTAAAAGAAGCCAATAAAACAATCCAAGAGAGCCTGCTCTCCTAAATCTTCAGGGGACAAAACCT
CCTACTTGTGATGCTTCAAAGCTGTCCAGAGCTACCAGTTTACCCCCAGAAGTCCCGACTCAATGCCTCTGATTGGAAAATAAATAGC
TTTGCACATAAAAACCACAGGCCAATTGTTAGCCTAGGAATGGGAAAATGCTGTGTGCATCA

>agCNE7_Homo-sapiens-hg19_GRCh37_11-31897563-31898031
GAATGCAGTTTGGCTGCCCTTGAGTTGAATGATGATCGTGGACCTTTGGAATGTCCACAAAGCCTCGCACTCTCGGCCTCCCCTGTCT
CTCTCCTTCGCCGGGGTAACAGATGGCCCTGGCTTTCACCAGCCGGGCCTGAGCACATTGTCACTCCCAGCTGAAAGTGAAATATTCA
GTGGCATTTAAATTAAATAGACTCTAATGCTTCAGTAATATTAATTACGTTTTCATTCATCCCTGGCCTAATAAATCAATGCGTAAAG
AGAGGATGCTCGGGAGGCTGGCAGCCTAGTGGGGCCACTGGTCCTTTGTGTGAGCCCGCACACAATGCCCCCATTCTTACGGGGCTGC
GCTTGCCCCTGCAAACATCTGTGCTCAGCCCCTCTTGAGGAGGAGCCAAAGAGGTAACATTTACACTCTGCTCCTCCAATTTGTCCCT
GCACCAGGGCCTGAGTCAACGGGAAATGT

>agCNE8_Homo-sapiens-hg19_GRCh37_11-31896241-31896769
CCAAGTGGCTGAGCTAATCCTTTCCTCTGCCTTCTGCCTGCTTCCCCTGTCACCTTAGGGGCCCCTCCTGAGGGGACATCTGTGAATT
CTCCATGGAATCCCTCATTTAGCTCCCAAGACCCAGCGCAAGCCTGGGTGCAGAATGTTGGTGCAATTTTGACAAGATGCTCACAGAG
GTTCAGTGACTAGTTTTAATTAGGCCATCTTTGTTTACGTTTGATGTTTATCAGCTAATTGGCATGGCAAGGTGTGAGTCACACAGCT
TTCCCCTGGCATGGAATCAGTTGTCAGAGAAAAAGCCAGGAGAACAAATGATCCCAATTATGAGAGAACCCACCGGGCTAATTATCGT
CCCACTAAGGACGGAACCTTCCCCCTCCTGCTGCTCCTGCTACTGCTGTAGCTGCACCAAGTGCCCTGGGATCTCTACACCCCTTCCT
CCAGCCCCAGAAACCCAGAAACCAGGATCTGGGCCCCCAGCTGGCTGCCTGGAGATTTATTCTCTCAAATTCAAAACTTGCATGTTCC
T

```
>agCNE9_Homo-sapiens-hg19_GRCh37_11-31847850-31848450
AGCCAGGCCAAGGCCTCCTCAGTGCGCAGGCGCCGTTCGGGCCCGCCGCCCCCTCCCAAGGCGTGGGGTCCCGGGGACAGACCCCGAG
CCCCTCTCTGCGCAACTACCGCCTCTNNNNNNNNGACCATCTGGTAATTGTCTTTCTTTAGATCTTCCCTTAATTATGCTTTTAATTA
AAGATGGTTCAAGGCACCGGACCACATGAAGGGCAGTAATTACAAATTACATTACAAACCCGACAGACGTAGCAGGATCTGCTGGGAA
AAAGGTACCGGAATAAAAATTTGACTAAAGTTTGGGATAAATTAATCGGTGGCTTGAAGAACAGTAGCACATTTCTGATTATGTAGGC
TCCAGCCAGGATTTAAGTTTTGTGTCATGATTGTTTTCATATTTCTTTGACATCTATTGATTTGGAGGCAGCTGCAAGCCTTTGTCAC
AGGAGAGAAGAGCAGTCACAATATATTAGCTTGATGGCATAACTCGAACCGGAGAAGCGAGGGGAGCCTGAGTGACTGGGGCGCACAT
GACCCCCCTCCTGGGGCCTTTGGGCTCAACCCTTACTTTTAACTTGTTCTAACAGGAATAATGGAGTAGTACT

>agCNE10_Homo-sapiens-hg19_GRCh37_11-31846835-31847159
TAGCCGGCCCGCAGCGCCAGAGAAGCCGCTCACTCCGGCGGAGTCTCTGCAAGAGAATCTGAGGCTGGGGAATAAAAGCCGAAACTAA
ATCAGGCAGCAAGAGTAACAAACCATCTGGAAAATGGACCCACGGAGCCGCAAGAATGTACNNNNNNNNNNNNATCCTTGACAACTTGT
GTCCTAAAGAAAGTGTATTGAAGATAATTGAAATGATTACAATTCATTGCTGCCTGACTTTGAATATGAACAGTCACATAATGACATC
CCCGCCACACAACAACAGTTTAATTTCCATTATCCGACTTCGGGATCCTAGCGCCAGCCGC

>agCNE11_Homo-sapiens-hg19_GRCh37_11-31845331-31846404
CGTCTCCCTCCGCTCCTCAGCTTGGCTACTTGGTGCTCGGACCCCGCATCCTAGTCCTCGTCCTTGTGGGACAGAGGCCAACCTTGCT
GTCGAGGCCCCAATATGTCACCACAAACACTCCCAGAACAAGAGCATCAACCCCAAGCTCCAGAACTTCTGGTCTTAGCCTACTGGAG
CCTGCCACCAAATTAAACACCGCGAAGGCTTTGAATCCCTGCAAAGACGGAGCCNNNNNNNNNNNNNNNNNNNNNNNNNNNNNNNNNN
NNNCAAGTTACATGGAATTAGTTGGAAGCGGGCGCATTCCCCAGCGACCGCAGCCATTAACATTATAATCTCAAACGCCATTTTTTAT
GTAACACTATTGTTAGTAAATCAACTCCTCAGGTCCTCAGAGACGCTGACCCCTGGCAATTCAGTGAAAGTGTAATTATCTCCCCGAA
TCTTGGACAGATTTAAGAGATTACATTTCAACTAAATCTATATCAATGCTAATTCCCTCTCTCCCCCCAGCCTGCAGGTGCTAGGCAC
GGGAAATGGCGTCAGGCCCCAGCAGAACGCTTCACAAAGAGGAGAGGGCTAGCTGTGTACAGGGCCAAAAGAAATGAAGCCCCACATA
AAATTACACCNNNNNNNNNNNNGCTACAAAACCTGGTTCTTATATAGCAAAGTTAAACAAAGCAGCTATGCTAAATTAGCGTTACATAA
AGGCGAGTTTAATGCTCGGTTACAGTCAACTGGTGTATCTCTGATGGCTAATTGTCTATCAAAATTAAAGAGAAAAGAACAAATGATT
CGTTATAAGAAGGTGTTAAAAGCCTCAACAAAAGGACATTTCACAAATATAATATCGGCAGCTCCCGGATTGCCTGCGGGGTTGTTCA
GGCCCCGGGCCTGCACAGCTATGAGAAGTGGGGTGCAGGGTGCTATTTGCCCAGCAAAAGCTCATTTCCTCCCAAATGAGTATAATGA
AAACCCGAGAGGCGATATTAGTGTAAAGATCGGGATTAGTCCAAATAGGAGCCGCGGACTTGGGCTTCAAAGGCGACCCCTACTCTTT
TTGCCCAGTCTGGGGGCG

>agCNE12_Homo-sapiens-hg19_GRCh37_11-31843629-31844067
GGGAGATGAGGGGGTTTCAACTGAGTCCCCTCCCTTCCCCCAAAAGGTCCATGTGATCGAGTTGACTTTTTTACTTTAAATCTTAAAT
AAGATGTGAGTTGTAACAAGAGGATTGGCACTTACTCCGTGACCCTTATAATTGGATTAAATATAGACTGAGACGTACAAAAACACAC
CAAGATTGACACCTGCCAACTGGACTTCCTTATGATTGATTGTGATGCTTCGTGCTGGCAACAATAATGAAATAAATTGTACGGATAA
AACAGCACATTTGTCATTTCCGTGCCATGAGTGAGGAAGTCAACGAGTTAGGCAAGCTGTTAGCTGACAGGCGCGAGCGGGAGGCAAG
CTAAAGAGATAAATAAGGATTCCAGGCATTGACAGGACAAATTATAACTTGTCACAACCTTTTTAAATTCAGAAGTTTTCAATTAAC

>agCNE13_Homo-sapiens-hg19_GRCh37_11-31843215-31843530
CAACAATCAATTTGAGAAAACTCTGCTTTTTCTTTCCTTTTAAACATTTTCATATTAAGTCAGTTTGTGGCACCATGTTTGATTTCTT
TCTCTCCCTTGTCCGGGTAGAGATGGAGACATTAGCTGAATTCCCTGGGGGTGATTGGATGCAATTCACTGAAAGTAGACAGGCACGT
CCTGGATGTTAGAAATTCACTTTTTCTCAACGTGACAAGGCCGGCTTCGATCTACAATTGTATTTGAATGGGCAATGAGCGGAAAGAT
CTCTCATCATTAAGCTTGCTTCTACTTTAGCAAAATAAGGAGAGCCTTCATT

>P2-agCNE14_Homo-sapiens-hg19_GRCh37_11-31841310-31841722
AGACTCCGCCGTCCGCCTTGGCGTGTGGGCTGCGGATAGGCCTCGGACCTGTGTAGGCCTTCCTAGCTGGCCAGTTTCTGTCCGGGTC
TCGCTCCCCACACTGTACCAACCCTAGGCCGCCGAGAGGGCTGTTATGGGCTTGTTTGGAGTCCAGGCCATTAAGTGTGCGCAAATCC
CTGCAACCCAGCAGGGTGCTCGAGAGCGACTCTGGAAACGCCACGGCCCCCGCCTGCTTATCTGCCTGTCAATAAAGGGCGCGTTCGG
GCTGCGCGGGAGCGCCGGTGGTGCAGGCGAGGCCCGAACTAGGCCCTTCCGAGCTCCGGGCCAACGCACGAGCCGCTCTCCATTTGTC
CCGGGCAAGGAGGCTGGGAGGGAGGGAGCCGGCTCTGGGGTCGCCGAGGGAGAAGGTGCGG

>P0-agCNE14_Homo-sapiens-hg19_GRCh37_11-31839356-31839510
CCTGGGAGCGGAGGGCGGGGAGGCAGGTGAGCCTGGCAGCGCCGGTTCCCACACTTCTCACCGCCGCTCGGCAGGGGAAGTGGCAGAT
CTGACAGCCGCGTTCTACGCGAGGACCTGCCCCAGAGTTTAAATGTCAATGATAAGAAAAGAGGGTG

>cre149_Homo-sapiens-hg19_GRCh37_11-31825492-31826050
TCTCCAACCTGCAGCCCCGACTTAGATAGCAGCCCTCCCAGGAAAAGACGTGGCTCCCTGTTCAGNNNNNNNNNNNNNNNNNNNNNNN
NNNNTGGAGCCTACTGATAAATTGACGCCAGGAGCCTGAGCTTCTTAGCAATAAATAAGCTCCAAATATAGAAGAGGGGGGAAAATAT
GATGAGCCTCTCTGACATTTGTCTTTAAAATAAAACTAGCTGCACGTCAAGTTTGAGCTTAATTTCTGGAAATAGGCGGAAGTCGCTC
CGGATCATGCATGGAAGGCTAATTGAAAAGATCAGTCGGGGCGCTTGTGGCAGCCTTGTCACTTTGTGACAGTGCTCCGCTCCGGGAA
ATTGCATCGTCACGACAAACGGGACCGTGATAAAACGACCCTTTCCGTCCTTATTTGTAGATCACTCAGACGAGATTGAACTGCACTT
GTTTCCCCTTCGAGGGGAGCCGCGTTTTCAGGGTAGCCGAAGGCTTGGGGCTGAGGGGGGGCCCTCACCAAGGCGCGGGTGGGGGCCG
GAGCCTCAACTCGATGAGAAGTGACAGGCGT

>E+120_Homo-sapiens-hg19_GRCh37_11-31712679-31713314
AGATGAATTTCTTAATGTGTACATTATGTTTTCTGAGCCTGGAAATTAAGGGGAAAAATGATGAAGGCATGTTACACAAGTACTTAAN
NNNNNNNNAAATCTCGGATAGTACTATGGTAATTAATGCTTATTTTATGCAAACTAGACTATATCTGTTAACGGACACATTACTGTCA
AGAAAATATTACTTGTCTTAATTATCAGTAATGAAGCAGGGATAAGCTGCTCACTTAGATATAATATATCTAATGACCTTTTCAGTTA
GAGGTTGTGATGAAAGGTATCAGTTTCACAAATTATGCTTCATTCAGCTTCTGGCCTTTGTGTAGATTAATGTTTTGTGGATGAGTTT
TAAAGTCAGGCATAAATTGTGTTTGGTGTTTAAAATTGATTTAGTGTCTTGCTTTTAGTACATCATTTTCTCATTTATAAAGCCTCAG
TTTTATCTTCAAGCTTAAAAAAATAATCCATGGTAAATTTAGAATTGCCTTACTGGCAGTGGTTTTTTTAACCTTACCAAATAGAATG
GTACATATGTTTACACTTGTTTAACTAAAGGACATAGTTTATGACTGAAGTTTCTTTCTGATTTCTGAATTTAAAACAATAAGCACAA
TGTTTACAATAACAATTTTT

>Simo_Homo-sapiens_GRCh38_11-31664297-31664497
```

AGATAAACTTTCCCATTGTCATTAATGAGGATGGATAATACTGAGTGACAAGTGCATTAGGAATGACACAGAAAGAGCTGTTAGATTT
GTCAGAGCGTATaACTTCAGGCATTGACACTTGAGGACATTCTTTTTGTGGATATTTGGTTNNNNNNNNNAGTCTGATTTAAAGATACC
AAAGTTTGTGTTTGCTAATAGAATA

>cre21_Homo-sapiens-hg19_GRCh37_11-31676830-31677382
CTATCAAAAGTGCATACCACCTTAAGGAGAACACAGTTTTGAGAATCTGTCTAATCCTATTTCTTCCCACTTTGGAATCTTAATTAGA
AGAGATAAGNNNNNNNNNNNCATCATTTAACTAAATTATTGTCTTGAATCTGTAGCAAAAGGTATAATCCGCAATTGGGCCAATTAGT
GAACCCCATAGTTAGCTATGCTGCAGCAAACAATTTGATTAGATCTGACACCATCCTAGTTCCCACTGTTGTTTTGCCAGGTATGATG
GTCAGACTTCAAAGAGCTTGAGGCATCAGCAGGGCAAGGTTTGGAGGCCAGCTTAGGTCCGGTTGTTAATCTCTCTGCTAAAACCACA
GCAGCTCTCACACAAAACAAGCTTACACGACACAATCATGCGGTATTAAAGTTAGGGTTGAGTCATGCCAATTTCCCTTTGATTTTTT
GATGATTGACCAAAGATGGCTACATGACTAGACACATGCTAAAAGTATCCTGATTAGGTAAGTTCCCAAAATATTTGAAGTATTTTTT
AACTTCCTATATAAATGTTGTGAAA

(B) Teleost analysis

>zs1_Danio-rerio_ZebrafishStickeback_danRer10_7-15582383-15582467_PAX6B_ANCORA
AGAAGCTCTGCTATTGGGCTTGGGGCTGTCTGTAACTCGAGATGGGCGTGGACGCCACGTTGCAAGATGACACGCAGAAGCGCC

>zs2_Danio-rerio_ZebrafishStickeback_danRer10_7-15585839-15585912_PAX6B_ANCORA
TGATTCAAATTGACAGAGCAAGTATAATTTAAATACATTAACAATTCATTAGTAAAATAAAAGGCCATTTCCC

>zs3_Danio-rerio_ZebrafishStickeback_danRer10_7-15604980-15605028_PAX6B_ANCORA
ACAGCTGTCCCCGAATCAATAGATGTCAAAGAACTATTAGGGCAGTTA

>zs4_Danio-rerio_ZebrafishStickeback_danRer10_7-15608192-15608252_PAX6B_ANCORA
GAGTCTATTTTCCAGATGGTTTGCTGTTGTTATTGCCTGATTTAGTGTGAGTTGTCGAAC

>zs5_Danio-rerio_ZebrafishStickeback_danRer10_7-15615903-15616048_PAX6B_ANCORA
ATTTTGAACAGAGCAATACGTTTCCACTAAACGTTATTAAATCGCGTGGAGTGACGAGCTTTTTATTTGTGCTGTCAGAGCATCTGAC
AGCCTCGTTAGCTCCCATTTCGCCCTGTCGGCTCACAGTCTGCCACAGACACTGAGT

>zs6_Danio-rerio_ZebrafishStickeback_danRer10_7-15616468-15616614_PAX6B_ANCORA
ATTGTGGGTCGGATACTCAGGCTTGTCACATTGAGGTAAAGTGAGTTTCTAACATCCAGGGCTTTGCTGTCTGCGCCGGACAAATTGA
TTGCGATCAGCCCGAGGGAGTTCTGCTAATGTCTCTCTCCGGGGCCGGGGGTGCATGG

>zs7_Danio-rerio_ZebrafishStickeback_danRer10_7-15618162-15618289_PAX6B_ANCORA
GGCCGGCTGAACAGATTTCCCTTCACGCCTGGTCGGGTTCTTGAGAGCGGGGCGTCGTGGATCAGGGAACGGAGGCACCTGGTCCGAG
CGCCGCGCAGCGTCTTCTTTTATTGACCGCATGATAAGC

>zs8_Danio-rerio_ZebrafishStickeback_danRer10_7-15618289-15618355_PAX6B_ANCORA
CGTGCGGTTTCCAGCGATCACCTGGAGAACCCTGCAGCGTTGCACGGTTTCTGTAGCTCTTAATTG

>zs9_Danio-rerio_ZebrafishStickeback_danRer10_7-15618437-15618486_PAX6B_ANCORA
TCATTTCCTGAGAGACACACTGCAGAGCTGTTGCGCATTTACGCACCGT

>zs10_Danio-rerio_ZebrafishStickeback_danRer10_7-15620809-15620852_PAX6B_ANCORA
TCGTATTAGCAGTTACTAATCCCAATTGGGCTAATTGCTTTGC

>zs11_Danio-rerio_ZebrafishStickeback_danRer10_7-15621389-15621448_PAX6B_ANCORA
TCTACAGTCGAACACGGGCGCAGAGGGGCTGCTTTCGGGTGTACCGATGTGTGCATAAA

>zs12_Danio-rerio_ZebrafishStickeback_danRer10_7-15624379-15624496_PAX6B_ANCORA
AATGTTTATTTGTTAATGGGAAAGAGTTAAAAGTCATTAGGCCTGAACAGTTTTTTTGGAGCACGGGAATGCACTGACCCGTTGAGGA
AAGGTGTCAGTTTGATTCATCAGCCGAGT

>zs13_Danio-rerio_ZebrafishStickeback_danRer10_7-15625028-15625140_PAX6B_ANCORA
CACTGTTTCCTGAATAAGCATGTTTAATTAAAGCAATTGTAGTTAGGGATTACACAAACGAAAGAAATGCAGATGAGATCCTAGTTAA
CCTCATAAACAACAACAACAGGCC

>zs14_Danio-rerio_ZebrafishStickeback_danRer10_7-15626081-15626213_PAX6B_ANCORA
GGGAGAGAAAATGAAACGAAAGACTTTCCTTCCCTGGGCCCCTTTTTAGCAATGTCACTGAATAGATCCTTTATTGCAATTGTTTTTT
TCCAATTGAGCACCCTACTGGGCTGTTGCATCCTTTTACAACCC

>zs15_Danio-rerio_ZebrafishStickeback_danRer10_7-15626391-15626543_PAX6B_ANCORA
GGGGGCAGGGTGCCCTCGGCTCTTTGTATTCGGGCAAGTATTGGTAGGTTTGGTGGGAGGTTGTGTTGCTAGTTGCAGAGGCCGAGTT
TTGCTGGCTGAGTTTGGGTATAGAAGGTAAAAGAGGCGCATTGTTGAGCTCCAGCAGGCTTTGT

>zs16_Danio-rerio_ZebrafishStickeback_danRer10_7-15626550-15626588_PAX6B_ANCORA
CTCTCACCCCCTCCAGTTCTCACTTTTCAGAGCACTGA

>zs17_Danio-rerio_ZebrafishStickeback_danRer10_7-15628415-15628466_PAX6B_ANCORA

CACCATGGGGAATTATATGATTATTAAGACTTAACCTTTGAACGGTGAAAT

>zs18_Danio-rerio_ZebrafishStickeback_danRer10_7-15628927-15629102_PAX6B_ANCORA
GAGCAGAATCCAATTTGGTCTGATATCCAAATGCAGACAGAAAGGGTCGTTTTATCATGCTACTATTTGTCGTGACGATGCGTTTTTC
AAAAGCAGAGCAGTGTCATATAAAGTGACAGTCCTGCCACAAGTACTTCAGCTGATCTTTTCAATGAGCCTTCCATGCATGATCCGA

>zs19_Danio-rerio_ZebrafishStickeback_danRer10_7-15630841-15630888_PAX6B_ANCORA
TAGTCTTAAATAATGTATGTGAATTGTGTATATTGCTGTCCATTCAG

>zs20_Danio-rerio_ZebrafishStickeback_danRer10_7-15634188-15634224_PAX6B_ANCORA
GGCACAGCTCTGTCTCTCTGTAATTGCTCATTAGAG

>zs21_Danio-rerio_ZebrafishStickeback_danRer10_7-15636221-15636303_PAX6B_ANCORA
TGATTAATGACAGTGGTGTGAGGAGGTTAACACACTGAACTCAAAGCGCTGTTGACTAGATGCTTGTGCGGGACAAAGCTGA

>zs22_Danio-rerio_ZebrafishStickeback_danRer10_7-15636384-15636453_PAX6B_ANCORA
TCATGGAGAAAAGCAGCTCTCTATTCAAGTGTTGGTGGTCACCTCAATGGGTCCCGGGACACCCATATG

>zs23_Danio-rerio_ZebrafishStickeback_danRer10_7-15650282-15650336_PAX6B_ANCORA
GAAGCCATTGTGTGGCAGAATGAGTTATTTTGCTTGACTTGCTGCCTCAGCAGG

>zs24_Danio-rerio_ZebrafishStickeback_danRer10_7-15665054-15665107_PAX6B_ANCORA
AAGGAGCAGAGGACAAACACGTGTAAGTGCTGCTTTCATATCCGATCGGCCGA

>zs25_Danio-rerio_ZebrafishStickeback_danRer10_7-15668932-15669082_PAX6B_ANCORA
AATGAATTGGGGGCGTAATCCATCACAGGTTGTAACTCTTCACAAGAAAGAGTCCCTTTAAGCAAAGGACAGCACTTGTGGGGACAAA
GGCAGGCATAGTGTGGACTTCATACTGCAGAGCTGAAAAGGTGTTGCCGTTAGTAACCTGAT

>zs26_Danio-rerio_ZebrafishStickeback_danRer10_7-15673115-15673194_PAX6B_ANCORA
TGGCTACAGTTTGCGCTGTGCAATTGAATTTCACACAATATGAGGCAGCAAATGACATGTAAATCATGAACAGCGTCCG

>zs27_Danio-rerio_ZebrafishStickeback_danRer10_7-15673414-15673466_PAX6B_ANCORA
GTAATTGTGTTTGTCCACCTGGGTATAAAATAAGCATTCTGGCGATGTGATG

>zs28_Danio-rerio_ZebrafishStickeback_danRer10_7-15673495-15673626_PAX6B_ANCORA
GGAGGGGAGCAGCCATGAGTGGATACACTTCATTACCCCCTAGATTCTCCCTCCAATTACCCCTAAATAAATACACACAGCCTACTGG
CAACAACAATGACATATAGCCCGAAAGGAGAATGAAAGGAGAC

>zs29_Danio-rerio_ZebrafishStickeback_danRer10_7-15679156-15679281_PAX6B_ANCORA
CTTTTCAGCTGGCTTTGAAAGGGAAATTAATTATCTCAGATACATGTAGCTCTTCGGTGGGTCTGGGCTTATTTGCATTGAGGAATAT
TCAGCAGAAGTAATGGATGCACAATCGAAATACTTGG

>zs30_Danio-rerio_ZebrafishStickeback_danRer10_7-15679480-15679569_PAX6B_ANCORA
ATGTAATGACATGGCAACCTGACAGTTGACGTCTTTATCTAAAATGTAAGAATTTGCATTCTAATTATGGATGAATTTACATCTTAAT
C
